# Phytochemicals of *Euphorbia hirta* L. and Their Inhibitory Potential Against SARS-CoV-2 Main Protease

**DOI:** 10.3389/fmolb.2021.801401

**Published:** 2022-02-04

**Authors:** Ruel Cayona, Evelyn Creencia

**Affiliations:** Department of Chemistry, College of Science and Mathematics, Mindanao State University—Iligan Institute of Technology, Iligan, Philippines

**Keywords:** *Euphorbia hirta*, COVID-19, molecular docking, phytochemical mining, medicinal plant, Philippine medicinal plant, SARS-CoV-2 Mpro, virtual screening

## Abstract

*Euphorbia hirta L.* is a medicinal plant widely used in the Philippines and across tropical Asia against various diseases, including respiratory disorders. In this study, the phytochemical components of *E. hirta* were investigated *in silico* for their potential to inhibit the severe acute respiratory syndrome-coronavirus-2 main protease (SARS-CoV-2 Mpro), a coronavirus disease 2019 (COVID-19) drug target that plays a critical role in the infection process of SARS-CoV-2. Phytochemical mining in tandem with virtual screening (PM-VS) was the strategy implemented in this study, which allows efficient preliminary *in silico* assessment of the COVID-19 therapeutic potential of the reported phytochemicals from the plant. The main rationale for considering *E. hirta* in the investigation was its reported efficacy against respiratory disorders. It is very promising to investigate the phytochemicals of *E. hirta* for their potential efficacy against diseases, such as COVID-19, that also target the respiratory system. A total of 298 *E. hirta* phytochemicals were comprehensively collected from the scientific literature. One hundred seventy of these phytochemicals were computed through molecular docking and were shown to have comparable or better binding properties (promising inhibitors) toward SARS-CoV-2 Mpro than known *in vitro* inhibitors. In connection to our previous work considering different medicinal plants, antiviral compounds were also rediscovered from the phytochemical composition of *E. hirta*. This finding provides additional basis for the potential of the plant (or its phytochemicals) as a COVID-19 therapeutic directly targeting drug targets such as SARS-CoV-2 Mpro and/or addressing respiratory-system-related symptoms. The study also highlights the utility of PM-VS, which can be efficiently implemented in the preliminary steps of drug discovery and development.

## 1 Introduction

*Euphorbia hirta* L. (Euphorbiaceae) is a medicinal plant widely used in the Philippines and across tropical Asia, and it is commonly known by the following names: “asthma plant” (English), “tawa-tawa” (Filipino), and “mangagaw” (Cebuano). The extract of *E. hirta* is taken orally as an aqueous decoction for most of its folkloric uses. As its English common name suggests, the plant has been used for asthma and other respiratory difficulties (Ekpo and Pretorius, 2007; Ogunlesi et al., 2009; Rao et al., 2017). In addition, available studies conclusively suggest its potential against dengue (Guzman et al., 2016; Perera et al., 2018; Suganthi and Ravi, 2018); however, additional studies are required to validate the results (Perera et al., 2018). Nevertheless, the studies reveal *E. hirta* as a pool for compounds with interesting biological activities.

*E. hirta* is one of the medicinal plants currently being investigated in the Philippines for its potential against coronavirus (CoV) disease 2019 (COVID-19) (Luci-Atienza, 2021a, Luci-Atienza, 2021b; Tawa-Tawa Clinical Trial on COVID-19, 2021). The goal is to develop a formulation utilizing the plant as an adjuvant treatment for mild to moderate COVID-19. A recently published review article identified *E. hirta* as one of the Philippine medicinal plants with immunomodulatory effects and potential against severe acute respiratory syndrome-CoV-2 (SARS-CoV-2) (Dayrit et al., 2021), the virus responsible for COVID-19. In this connection, a parallel and complementary *in silico* study was conducted to investigate the potential of its phytochemicals against a specific COVID-19 drug target, SARS-CoV-2 main protease (Mpro). Mpro is seen as an important COVID-19 drug target because of the role it plays in the regulation of viral replication (Di Micco et al., 2021).

It was the reported activities of *E. hirta* or its phytochemicals against respiratory-related ailments that serve as the primary basis for considering it as a subject of the present investigation. This study was conducted in line with the ongoing effort to discover potential COVID-19 therapeutic chemicals from medicinal plants, starting first with those found in the Philippines (Philippine medicinal plants). A strategy called phytochemical mining in tandem with virtual screening (PM-VS) was implemented. PM-VS refers to the systematic and comprehensive collection of medicinal plant phytochemicals reported in the scientific literature (phytochemical mining) and subsequent *in silico* assessment of the potential efficacy of the phytochemicals against specific or multiple drug target(s) (virtual screening). PM-VS and its rationale have been elaborated elsewhere (Cayona and Creencia, 2021a; Cayona and Creencia, 2021b, Cayona and Creencia, 2022). Specifically focused in this study is *E. hirta* and automated targeted molecular docking as the medicinal plant and virtual screening tool, respectively. It is argued that PM-VS can be efficiently implemented in the preliminary steps of drug discovery and development.

## 2 Materials and Methods

### 2.1 Phytochemical Data Collection

The method implemented in this study is adapted from the method described in our previous papers (Cayona and Creencia, 2021a; Cayona and Creencia, 2021b) with slight modification. The Preferred Reporting Items for Systematic Reviews and Meta-analyses (PRISMA) (Moher et al., 2009) protocol was implemented throughout the systematic data collection process. The sources of phytochemicals were peer-reviewed research and review articles from scientific journals deposited in the MEDLINE database by the US National Institutes of Health National Library of Medicine (https://pubmed.ncbi.nlm.nih.gov/). The Google Scholar (https://scholar.google.com/) search engine was utilized to find additional literature but other search engines were also consulted (i.e., Microsoft Academic and Semantic Scholar), similarly applying relevant search keys and filters when applicable.

The identified sources were then compared against each other to check for multiple entries and reference-checked to retrieve additional sources unintentionally omitted in the first part of literature gathering. Articles deposited in restricted repositories and which were not written in English were not included. Thereafter, the phytochemicals reported in every literature reference were trimmed down to unique chemical identities only (because one compound may have multiple reported names). For simplicity, the common names of the compounds were taken in cases where ambiguity does not manifest; otherwise, the International Union of Pure and Applied Chemistry (IUPAC) nomenclature was adopted. The study strictly adhered to the data collection protocol described in PRISMA (see Supplementary Materials).

### 2.2 Phytochemical Classification

The collected phytochemicals from *E. hirta* were classified according to the ClassyFire (Djoumbou Feunang et al., 2016) algorithm of chemical classification. This was done to gain insight that might be helpful in assessing the basic structure–activity relationship. The hierarchy of chemical taxonomic classification can be found in the Supplementary Materials.

### 2.3 Preparation of Ligands

Three-dimensional (3D) structure-data files (SDFs) of phytochemicals included in the final list were either conveniently collected from PubChem or manually generated whenever they are unavailable in the database. Hydrogen atoms were explicitly added to the structures. In some cases, two-dimensional (2D) SDFs were used but only for 2D compounds (linear or flat). In preparation for virtual screening and for future convenience, the SDFs of all the structures of the phytochemicals (the ligands) were combined into a single SDF using OpenBabel 2.4.1 (O’Boyle et al., 2011) to facilitate automated importing of the multiple structures into the virtual screening tool. The same preparation was done for the control compounds.

### 2.4 Receptor Preparation

The crystal structure at 2.16 Å of the SARS-COV-2 Mpro (PDB ID: 6LU7) in complex with the *in vitro* inhibitor N3 (Jin et al., 2020) was downloaded from the Protein Data Bank (http://www.rcsb.org/) in a PDB file format. The noninteracting atoms (e.g., water and buffer molecules) were removed, and hydrogen atoms were explicitly added to the enzyme and the native ligand.

The active site was taken as the region of the SARS-CoV-2 Mpro volume where the *in vitro* inhibitor N3 was attached. From the SARS-CoV-2 Mpro–N3 complex, the search space for the targeted molecular docking was then assigned with the help of BIOVIA Discovery Studio Visualizer v20.1.0.19295 (DSV, 2020). The interacting and the pocket amino acids (AAs) that lie within the 3.5 Å distance from the closest N3 atom were identified by visual inspection. The residues found within this region totaled 25 AAs. The interacting AAs were H41, M49, F140, N142, G143, H164, M165, E166, L167, P168, H172, Q189, T190, and T191; and the pocket AAs were T24, T25, T26, L27, Y54, L141, S144, C145, H163, D187, R188, and Q192. From this list of AAs, the H41–C145 catalytic dyad can be found (Wang YC. et al., 2020; Hakmi et al., 2020; Ullrich and Nitsche, 2020).

### 2.5 Virtual Screening Through Automated Molecular Docking

#### 2.5.1 Molecular Docking Tools

The phytochemical ligands were virtually screened against SARS-CoV-2 Mpro (6LU7-neat) using PyRx0.8 (Dallakyan and Olson, 2015), a virtual screening tool that allows automated molecular docking of multiple ligands (or libraries) against target receptor (s). PyRx0.8 utilizes the enabling capabilities of AutoDock tools for receptor and ligand preparation just as in AutoDock 4 (Morris et al., 2009) and the earlier versions; AutoDock Vina for molecular docking (Trott and Olson, 2010); OpenBabel for file format interconversion (O’Boyle et al., 2011); and other open-source software. To save on computational cost, targeted molecular docking on the active site of Mpro was conducted.

#### 2.5.2 Control Parameters

To enhance the accuracy, control parameters were set in molecular docking against Mpro. In addition to the phytochemical ligands, control ligand samples were also tested. Ten known inhibitors with established *in vitro* half-maximal effective concentration (EC_50_) against SARS-CoV-2 or half-maximal inhibitory concentration (IC_50_) against SARS-CoV-2 Mpro were used as positive controls. On the other hand, 10 small molecules that do not possess interesting pharmacological properties were also used as negative controls. The positive controls were N3 (6LU7 native ligand), efonidipine, bedaquiline, tideglusib, manidipine, N3, lercanidipine, boceprevir, shikonin, ebselen, and carmofur, whose inhibitory properties were reported elsewhere (Ghahremanpour et al., 2020; Jin et al., 2020; Ma et al., 2020). The negative controls chosen were anthracene, naphthalene, glycerol, decane, hexanol, benzene, cyclohexane, hexane, ethanol, and water. The positive controls (inhibitors) were expected to give satisfactory binding free energy (BFE) values towards the receptor SARS-CoV-2 Mpro because they are empirically established inhibitors. On this basis, their BFEs were taken as a reference in assigning promising phytochemicals against SARS-CoV-2 Mpro. In contrast, the negative controls should have unsatisfactory computed BFE towards the receptor. The control ligands provide a simple means to assess the reliability and performance of the virtual screening tool.

#### 2.5.3 Automated Molecular Docking

The receptor (6LU7-neat) was loaded onto PyRx0.8 and set into the macromolecule (receptor) in the PDBQT format. The collective SDFs of the phytochemicals and positive and negative controls previously prepared using OpenBabel 2.4.1 were also loaded onto PyRx0.8 and subsequently extracted automatically to individual structures. The structures were then energy minimized by implementing suitable force fields. For most of the structures, MMFF94 was sufficient in energy minimization; however, UFF and/or Ghemical must be implemented for some ligands whose final structures were distorted under specified UFF minimization parameters. Thereafter, the ligands were converted into a docking-ready PDBQT file format.

Before docking, the search space for the targeted automated molecular docking was set. The interacting and the pocket AA residues of SARS-CoV-2 Mpro that were identified previously were selected, and the search space was adjusted manually in the PyRx0.8 interface so that all of the residues were included in the grid volume of the search space. The resulting grid dimensions are the following: center_x = −10.8864; center_y = 14.0407; center_z = 68.7458; size_x = 21.4856; size_y = 26.7715; size_z = 28.0882. The exhaustiveness of the most stable conformation search was set at 16. Finally, docking was commenced using the Vina (AutoDock Vina) tab in PyRx0.8.

#### 2.5.4 Receptor–Ligand Interaction Analysis

Interactions of the ligands which have BFEs comparable to or better than those of the positive controls were analyzed. Those ligands whose most stable binding conformation (docking RMSD = 0) established interactions with the H41–C145 catalytic dyad (Jin et al., 2020; Khan et al., 2020; Menéndez et al., 2020; Mirza and Froeyen, 2020; Shitrit et al., 2020) of the SARS-CoV-2 Mpro and those with reported antiviral properties were given emphasis. Favorable computed BFE and catalytic dyad interaction(s) were considered as major criteria in identifying promising SARS-CoV-2 Mpro inhibitors.

#### 2.5.5 Assessment of the Reliability of the Tools and Strategies

All the tools and strategies used in the study are well established throughout the scientific literature. The number of citations of the articles that report the tools and strategies partly establish their reputation in the field. For example, Google Scholar queries on PRISMA, ClassyFire, PyRx0.8, AutoDock Vina, and AutoDock 4 will reveal 7,156; 283; 873; 15,275; and 12,351 citations, respectively, as of July 17, 2021.

## 3 Results

### 3.1 Phytochemical Mining and Classification

Literature reports indicate that leaves, aerial parts, and whole plants are the sources of *E. hirta* phytochemicals. The relevant data collected from phytochemical mining (PM) *E. hirta* are presented in Table 1. Each phytochemical is provided with its molecular formula (MF), BFE value against SARS-CoV-2 Mpro, and chemical taxonomy grouping levels (ClassyFire Superclass, Class, and Subclass). The chemical structures of all *E. hirta* phytochemicals and the control samples (positive and negative) used in molecular docking can be found in the Supplementary Materials. In total, 298 phytochemical components of *E. hirta* were identified from verified sources. This is by far the most comprehensive data gathering for *E. hirta* phytochemicals. The phytochemicals gathered fall into 13 ClassyFire Superclass levels. Majority are lipids and lipid-like molecules (Lipids) (108, 36.2%); phenylpropanoids and polyketides (PPPKs) (82, 27.5%); organic oxygen compounds (OOCs) (29, 9.7%); lignan, neolignans, and related compounds (Lignans) (27, 9.1%); organic acids and derivatives (OADs) (16, 5.4%); benzenoids (15, 5.0%); and organoheterocyclic compounds (OHCCs) (13, 4.4%), comprising a total of 97.0%. The rest (*Miscellaneous) of the phytochemicals are hydrocarbons, organic 1,3-dipolar compounds, organic nitrogen, organohalogens, and organic salt.

**TABLE 1:** Phytochemicals from *E. hirta.*

ID	Phytochemical	MF^a^	BFE^b^
*Benzenoids/benzene and substituted derivatives*			
**1**	1-(3-aminophenyl)ethanol (Rautela et al.,^2020^)	C_8_H_11_NO	−4.6
**2**	1-*O*-butyl 2-*O*-tetradecyl benzene-1,2-dicarboxylate (Ogunlesi et al.,^2009^)	C_26_H_42_O_4_	−4.9
**3**	benzoic acid (Ali et al.,^2020^)	C_7_H_6_O_2_	−4.5
**4**	benzamide, 3-fluoro-*N*-butyl-*N*-ethyl (Rautela et al.,^2020^)	C_13_H_18_FNO	−4.9
**5**	gallic acid (Bach et al.,^2020^; Linfang et al., 2012; Mahomoodally et al.,^2020^; Mekam et al.,^2019^, Suganthi and Ravi, 2018; Wu et al.,^2012^)	C_7_H_6_O_5_	−5.5
**6**	ethyl gallate (Mekam et al.,^2019^)	C_9_H_10_O_5_	−5.7
**7**	methyl gallate (Mahomoodally et al.,^2020^)	C_8_H_8_O_5_	−5.6
**8**	protocatechuic acid (Mahomoodally et al.,^2020^)	C_7_H_6_O_4_	−5.4
**9**	1-(3-ethoxyphenyl)propan-2-one (Rautela et al.,^2020^)	C_11_H_14_O_2_	−5.0
**10**	methyl 3-(3,5-di-*tert*-butyl-4-hydroxyphenyl)propanoate (Perumal and Mahmud, 2013)	C_18_H_28_O_3_	−6.5
*Benzenoid/naphthalene*			
**11**	[6-(4-cyanophenyl)naphthalen-2-yl] hexanoate (Rautela et al.,^2020^)	C_23_H_21_NO_2_	−6.5
	*Benzenoids/phenols*		
**12**	benzene-1,2,3-triol (Karki et al.,^2019^)	C_6_H_6_O_3_	−4.9
**13**	2-*tert*-butyl-4-methoxyphenol (Rautela et al.,^2020^)	C_11_H_16_O_2_	−5.1
**14**	4-ethenyl-2-methoxyphenol (Rautela et al.,^2020^)	C_9_H_10_O_2_	−4.7
*Benzenoid*			
**15**	1,2-benzenedicarboxylic acid diisooctyl ester (Ogunlesi et al.,^2009^)	C_24_H_38_O_4_	−5.4
*Hydrocarbon/saturated hydrocarbon*			
**16**	Tetradecane (Ogunlesi et al.,^2009^)	C_14_H_30_	−4.2
*Hydrocarbon/unsaturated hydrocarbon*			
**17**	(*E*)-pentatriacont-17-ene (Rautela et al.,2020)	C_35_H_70_	−4.4
*Lignans, neolignans, and related compounds/aryltetralin lignans*			
**18**	Isolintetralin (Zhang et al.,^2020^)	C_23_H_28_O_6_	−7.1
**19**	Lintetralin (Zhang et al.,^2020^)	C_23_H_28_O_6_	−7.3
**20**	Phyltetralin (Zhang et al.,^2020^)	C_24_H_32_O_6_	−7.0
**21**	Hypophyllanthin (Zhang et al.,^2020^)	C_24_H_30_O_7_	−6.9
*Lignans, neolignans, and related compounds/dibenzylbutane lignans*			
**22**	Niranthin (Zhang et al.,^2020^)	C_24_H_32_O_7_	−6.3
**23**	5-demethoxyniranthin (Zhang et al.,^2020^)	C_23_H_30_O_6_	−6.3
**24**	Phyllanthin (Zhang et al.,^2020^)	C_24_H_34_O_6_	−5.9
*Lignans, neolignans, and related compounds/furanoid lignans*			
**25**	Virgatusin (Zhang et al.,^2020^)	C_23_H_28_O_7_	−6.4
**26**	Urinaligran (Zhang et al.,^2020^)	C_22_H_24_O_7_	−7.4
**27**	7-hydroxyhinokinin (Zhang et al.,^2020^)	C_20_H_18_O_8_	−8.2
**28**	(−)-pinoresinol (Li et al.,^2015^)	C_20_H_22_O_6_	−7.2
**29**	(+)-syringaresinol (Li et al.,^2015^)	C_22_H_26_O_8_	−7.6
*Lignans, neolignans, and related compounds/lignan glycosides*			
**30**	(+)-syringaresinol glucoside (Li et al.,^2015^)	C_28_H_36_O_13_	−7.0
**31**	(−)-pinoresinol glucoside (Li et al.,^2015^)	C_26_H_32_O_11_	−7.6
*Lignans*			
**32**	5-methoxyvirgatusin (Zhang et al.,^2020^)	C_24_H_30_O_8_	−7.2
**33**	7R-ethoxy-3-methoxyisolintetralin (Zhang et al.,^2020^)	C_26_H_34_O_8_	−6.7
**34**	7R-ethoxyisolintetralin (Zhang et al.,^2020^)	C_25_H_32_O_7_	−6.8
**35**	7S-ethoxyisolintetralin (Zhang et al.,^2020^)	C_25_H_32_O_7_	−7.6
**36**	chebulic acid triethyl ester (Yang et al.,^2020^)	C_20_H_24_O_11_	−6.2
**37**	euphorhirtin A (Yang et al.,^2020^; Zhang et al.,^2020^)	C_19_H_20_O_11_	−6.5
**38**	euphorhirtin B (Yang et al.,^2020^; Zhang et al.,^2020^)	C_19_H_20_O_11_	−6.6
**39**	euphorhirtin C (Yang et al.,^2020^; Zhang et al.,^2020^)	C_18_H_18_O_11_	−6.6
**40**	euphorhirtin D (Yang et al.,^2020^; Zhang et al.,^2020^)	C_18_H_18_O_11_	−6.8
**41**	hirtacoumaroflavonoside (Sheliya et al.,^2015^)	C_41_H_44_O_17_	−8.7
**42**	hirtacoumaroflavonoside B (Sheliya et al.,^2015^)	C_31_H_36_O_12_	−8.4
**43**	Neonirtetralin (Zhang et al.,^2020^)	C_20_H_22_O_7_	−6.7
**44**	3,5-*O*-dicaffeoylquinic acid (Mekam et al.,^2019^)	C_25_H_24_O_12_	−9.2
*Lipids and lipid-like molecules/fatty acyls*			
**45**	2-(dimethylamino)ethyl 3-cyclopentylpropanoate (Rautela et al.,^2020^)	C_12_H_23_NO_2_	−4.8
**46**	3-octadecoxypropyl (*Z*)-octadec-9-enoate (Karki et al.,^2019^)	C_39_H_76_O_3_	−4.2
**47**	ethyl hexadecanoate (Sharma et al.,^2014^)	C_18_H_36_O_2_	−4.4
**48**	ethyl octadecanoate (Sharma et al.,^2014^)	C_20_H_40_O_2_	−4.4
**49**	methyl (11*E*,14*E*,17*E*)-icosa-11,14,17-trienoate (Karki et al.,^2019^)	C_21_H_36_O_2_	−4.9
**50**	methyl 9-octadecanoate (Olaoluwa et al.,^2018^)	C_19_H_36_O_2_	−4.3
**51**	methyl hexadecanoate (Perumal and Mahmud, 2013; Olaoluwa et al.,^2018^; Karki et al.,^2019^; Rautela et al.,^2020^)	C_17_H_34_O_2_	−4.3
**52**	citronellyl palmitoleate (Rautela et al.,^2020^)	C_26_H_48_O_2_	−5.0
**53**	geranyl linoleate (Rautela et al.,^2020^)	C_28_H_48_O_2_	−5.2
**54**	(Z)-3,7-dimethylocta-2,6-dien-1-yl palmitate (Rautela et al.,^2020^)	C_26_H_48_O_2_	−5.4
**55**	oleic acid (Ogunlesi et al.,^2009^)	C_18_H_34_O_2_	−5.0
**56**	pentadecanoic acid (Sharma et al.,^2014^)	C_15_H_30_O_2_	−4.8
**57**	tetradecanoic acid (Sharma et al.,^2014^)	C_14_H_28_O_2_	−4.5
**58**	hexadecanoic acid (Ogunlesi et al.,^2009^; Perumal and Mahmud, 2013; Rautela et al.,^2020^)	C_16_H_32_O_2_	−4.4
**59**	methyl 3-hydroxyoctanoate *O*-beta-d-glucopyranoside (Nomoto et al.,^2013^)	C_15_H_28_O_8_	
**60**	*N*-butyl-1-*O*-alpha-l-rhamnopyranoside (Mallavadhani and Narasimhan, 2009)	C_10_H_20_O_5_	−5.2
**61**	*N*-butyl-1-*O*-beta-l-rhamnopyranoside (Mallavadhani and Narasimhan, 2009)	C_10_H_20_O_5_	−5.4
**62**	sodium beta-d-glucopyranosyl 12-hydroxyjasmonate (*acid form was used in docking) (Bach et al.,^2020^)	C_18_H_28_O_9_	−7.0
**63**	bumaldoside A (Nomoto et al.,^2013^)	C_19_H_36_O_10_	−7.2
**64**	byzantionoside B (Nomoto et al.,^2013^)	C_19_H_32_O_7_	−7.1
**65**	corchoionoside C (Nomoto et al.,^2013^)	C_19_H_30_O_8_	−7.2
**66**	Roseoside (Mekam et al.,^2019^)	C_19_H_30_O_8_	−7.0
**67**	(*Z*)-3-hexenyl-beta-d-glucopyranoside (Nomoto et al.,^2013^)	C_12_H_22_O_6_	−6.3
**68**	geranyl acetate (Rautela et al.,^2020^)	C_12_H_20_O_2_	−5.0
**69**	neryl acetate (Rautela et al.,^2020^)	C_12_H_20_O_2_	−4.9
**70**	(9*E*,12*E*,15*E*)-octadeca-9,12,15-trien-1-ol (Sharma et al.,^2014^)	C_18_H_32_O	−4.7
**71**	heptadec-13-yn-1-ol (Ogunlesi et al.,^2009^)	C_17_H_32_O	−4.4
**72**	(*Z*)-octadec-13-enal (Karki et al.,^2019^)	C_18_H_34_O	−4.1
**73**	(*Z*)-tetradec-9-enal (Karki et al.,^2019^)	C_14_H_26_O	−4.5
**74**	hexadecanal (Ogunlesi et al.,^2009^)	C_16_H_32_O	−4.2
**75**	(*Z*)-octadec-9-enamide (Olaoluwa et al.,^2018^)	C_18_H_35_NO	−4.2
**76**	tetradecanamide (Olaoluwa et al.,^2018^)	C_14_H_29_NO	−4.5
**77**	(1′,*R*,5′*R*)-5-(5′-carboxymethyl-2′-oxocyclopentyl)-3-*Z*-pentenyl acetate (Chi et al.,^2012^)	C_14_H_20_O_5_	
**78**	methyl linolenate (Perumal and Mahmud, 2013; Rautela et al.,^2020^)	C_19_H_32_O_2_	−5.2
**79**	methyl linoleate (Rautela et al.,^2020^; Sharma et al.,^2014^)	C_19_H_34_O_2_	−4.4
**80**	glyceryl monolinoleate (Rautela et al.,^2020^)	C_21_H_38_O_4_	−5.1
**81**	ethyl linoleate (Rautela et al.,^2020^; Sharma et al.,^2014^)	C_20_H_36_O_2_	−4.4
**82**	linolenic acid (Rautela et al.,^2020^)	C_18_H_30_O_2_	−4.9
**83**	linoleic acid (Perumal and Mahmud, 2013)	C_18_H_32_O_2_	−4.6
*Lipids and lipid-like molecules/glycerolipids*			
**84**	2,3-dihydroxypropyl octadecanoate (Rautela et al.,^2020^)	C_21_H_42_O_4_	−4.5
**85**	2-monopalmitin (Perumal and Mahmud, 2013; Rautela et al.,^2020^)	C_19_H_38_O_4_	−4.7
**86**	2-monostearin (Perumal and Mahmud, 2013)	C_21_H_42_O_4_	−4.7
**87**	triolein (Karki et al.,^2019^)	C_57_H_104_O_6_	−4.5
*Lipids and lipid-like molecules/prenol lipids/diterpenoids*			
**88**	(*E*)-3,7,11,15-tetramethylhexadec-2-en-1-ol (Ogunlesi et al.,^2009^)	C_20_H_40_O	−5.2
**89**	phytol (Ogunlesi et al.,^2009^; Perumal and Mahmud, 2013; Sharma et al.,^2014^; Olaoluwa et al.,^2018^; Karki et al.,^2019^)	C_20_H_40_O	−5.1
**90**	gibberellin (Mekam et al.,^2019^)	C_20_H_28_O_6_	−6.2
**91**	ponicidin (Mekam et al.,^2019^)	C_20_H_26_O_6_	−7.5
**92**	albopilosin H (Mekam et al.,^2019^)	C_20_H_28_O_4_	−6.5
**93**	kaur-16-ene (Olaoluwa et al.,^2018^)	C_20_H_32_	−6.6
*Lipids and lipid-like molecules/prenol lipids/monoterpenoids*			
**94**	(*E*)-3,7-dimethyl-2,6-octadienoic acid (Rautela et al.,^2020^)	C_10_H_16_O_2_	−4.7
**95**	citronellol (Rautela et al.,^2020^)	C_10_H_20_O	−4.5
**96**	camphol (Shah et al.,^2019^)	C_10_H_18_O	−4.3
**97**	*cis*-alpha-bergamotene (Rautela et al.,^2020^)	C_15_H_24_	−5.0
**98**	2,6,6-trimethylbicyclo[3.1.1]heptane-2,3-diol (Rautela et al.,^2020^)	C_10_H_18_O_2_	−4.9
**99**	*para*-menth-3-en-9-ol (Olaoluwa et al.,^2018^)	C_10_H_18_O	−4.9
**100**	tricyclo[4.2.2.01,5]decan-3-ol (Rautela et al.,^2020^)	C_10_H_16_O	−4.8
*Lipids and lipid-like molecules/prenol lipids/quinone and hydroquinone lipids*			
**101**	gamma-tocopherol (Rautela et al.,^2020^; Sharma et al.,^2014^)	C_28_H_48_O_2_	−6.2
**102**	vitamin E (Perumal and Mahmud, 2013; Rautela et al.,^2020^)	C_29_H_50_O_2_	−6.7
*Lipids and lipid-like molecules/prenol lipids/sesquiterpenoids*			
**103**	isospathulenol (Rautela et al.,^2020^)	C_15_H_24_O	−5.9
**104**	beta-elemene (Olaoluwa et al.,^2018^)	C_15_H_24_	−5.0
**105**	neointermedeol (Rautela et al.,^2020^)	C_15_H_26_O	−5.5
**106**	germacren d-4-ol (Rautela et al.,^2020^)	C_15_H_26_O	−5.6
**107**	beta-bisabolene (Rautela et al.,^2020^)	C_15_H_24_	−5.7
**108**	*cis*-nerolidol (Rautela et al.,^2020^)	C_15_H_26_O	−5.3
**109**	alpha-humulene (Rautela et al.,^2020^)	C_15_H_24_	−4.9
**110**	alpha-farnesene (Rautela et al.,^2020^)	C_15_H_24_	−5.2
**111**	beta-caryophyllene (Olaoluwa et al.,2018); Rautela et al.,^2020^)	C_15_H_24_	−5.1
**112**	farnesol 1 (Rautela et al.,^2020^)	C_15_H_26_O	−5.2
**113**	2,6,10-trimethyltetradecane (Ogunlesi et al.,^2009^)	C_17_H_36_	−4.3
**114**	neophytadiene (Perumal and Mahmud, 2013; Rautela et al.,^2020^)	C_20_H_38_	−4.6
**115**	6,10,14-trimethylpentadecan-2-one (Ogunlesi et al.,^2009^; Perumal and Mahmud, 2013)	C_18_H_36_O	−5.0
**116**	taraxerol acetate (Li et al.,^2015^)	C_32_H_52_O_2_	−7.5
**117**	taraxerone (Ragasa and Cornelio, 2013; Li et al.,^2015^; Tayone et al.,^2020^)	C_30_H_48_O	−8.0
**118**	taraxerol (Perumal and Mahmud, 2013; Prachi and Pradeep, 2014; Li et al.,^2015^; Amos Samkumar et al.,^2019^; Bach et al.,^2020^; Tayone et al.,^2020^)	C_30_H_50_O	−7.8
*Lipids and lipid-like molecule/terpene glycoside*			
**119**	citroside A (Nomoto et al.,^2013^)	C_19_H_30_O_8_	−6.8
*Lipids and lipid-like molecule/triterpenoids*			
**120**	friedelane-3beta,29-diol (Li et al.,^2015^)	C_30_H_52_O_2_	−7.6
**121**	psi-taraxastane-3,20-diol (Li et al.,^2015^)	C_30_H_52_O_2_	−7.4
**122**	squalene (Perumal and Mahmud, 2013; Sharma et al.,^2014^)	C_30_H_50_	−5.4
**123**	lanost-8-en-3beta-ol (Rautela et al.,^2020^)	C_30_H_52_O	−6.1
**124**	lupeol (Ragasa and Cornelio, 2013; Tayone et al.,^2020^)	C_30_H_50_O	−7.3
**125**	friedelan-3beta-ol (Li et al.,^2015^)	C_30_H_52_O	−7.9
**126**	friedelin (Linfang et al., 2012; Li et al.,^2015^)	C_30_H_50_O	−8.2
**127**	alpha-amyrin (Linfang et al., 2012; Perumal and Mahmud, 2013; Ragasa and Cornelio, 2013)	C_30_H_50_O	−7.9
**128**	beta-amyrin (Martínez-Vázquez et al.,^1999^; Perumal and Mahmud, 2013; Ragasa and Cornelio, 2013)	C_30_H_50_O	−7.2
*Lipids and lipid-like molecules/steroids and steroid derivatives/cycloartanols and derivatives*			
**129**	(23*E*)-cycloart-23-en-3beta,25-diol (Tayone et al.,^2020^)	C_30_H_50_O_2_	−7.0
**130**	cycloart-23-ene-3beta,25,28-triol (Li et al.,^2015^)	C_30_H_50_O_3_	−6.8
**131**	cyclolanostan-3beta-ol (Li et al.,^2015^)	C_30_H_52_O	−6.7
**132**	24-hydroperoxycycloart-25-en-3beta-ol (Ragasa and Cornelio, 2013; Tayone et al.,^2020^)	C_30_H_50_O_3_	−7.3
**133**	25-hydroperoxycycloart-23-en-3beta-ol (Ragasa and Cornelio, 2013; Tayone et al.,^2020^)	C_30_H_50_O_3_	−8.0
**134**	cycloart-23-ene-3beta,25-diol (Li et al.,^2015^)	C_30_H_50_O_2_	−7.1
**135**	cycloartenol (Perumal and Mahmud, 2013; Ragasa and Cornelio, 2013; Li et al.,^2015^)	C_30_H_50_O	−6.9
*Lipids and lipid-like molecule/steroids and steroid derivative/ergostane steroids*			
**136**	campesterol (Perumal and Mahmud, 2013; Bach et al.,^2020^; Rautela et al.,^2020^)	C_28_H_48_O	−6.9
*Lipids and lipid-like molecule/steroids and steroid derivative/stigmastanes and derivatives*			
**137**	stigmasterol (Rautela et al.,^2020^)	C_29_H_48_O	−7.1
**138**	gamma-sitosterol (Perumal and Mahmud, 2013; Rautela et al.,^2020^)	C_29_H_50_O	−6.8
**139**	beta-sitosterol (Martínez-Vázquez et al.,^1999^; Mallavadhani and Narasimhan, 2009; Tayone et al.,^2020^)	C_29_H_50_O	−6.8
**140**	16alpha,17-dihydroxy-ent-kaurane-3-one (Li et al.,^2015^)	C_20_H_32_O_3_	−7.9
**141**	16alpha,17,19-trihydroxy-ent-kaurane (Li et al.,^2015^)	C_20_H_34_O_3_	−6.5
**142**	16alpha*,*19-dihydroxy-ent-kaurane (Yan et al.,^2011^)	C_20_H_34_O_2_	−6.1
**143**	16beta,17-dihydroxy-ent-kaurane-3-one (Li et al.,^2015^)	C_20_H_32_O_3_	−7.0
**144**	23(*E*)-25-methoxycycloart-23-en-3beta-ol (Li et al.,^2015^)	C_31_H_52_O_2_	−7.7
**145**	24-methylencycloartenol (Martínez-Vázquez et al.,^1999^)	C_29_H_50_O	−7.1
**146**	28-hydroxyfriedelin (Li et al.,^2015^)	C_30_H_50_O_2_	−7.7
**147**	2beta,16alpha,19-trihydroxy-ent-kaurane (Li et al.,^2015^; Yan et al.,^2011^)	C_20_H_34_O_3_	−6.3
**148**	3beta,16alpha,17-trihydroxy-ent-kaurane (Li et al.,^2015^)	C_20_H_34_O_3_	−6.9
**149**	3beta-hydroxy-cycloart-25-ene-24-one (Li et al.,^2015^)	C_30_H_48_O_2_	−6.5
**150**	3beta-hydroxyurs-12-ene (Mallavadhani and Narasimhan, 2009)	C_29_H_48_O	−7.7
**151**	ent-kaur-16-ene-3beta-ol (Li et al.,^2015^)	C_21_H_34_	−6.4
**152**	isojaponin A (Mekam et al.,^2019^)	C_21_H_30_O_6_	−7.5
*Organic 1,3-dipolar compound/allyl-type 1,3-dipolar organic compound*			
**153**	azidocyclohexane (Rautela et al.,^2020^)	C_6_H_11_N_3_	−4.3
*Organic acids and derivatives/carboxylic acids and derivatives*			
**154**	ethyl 1-ethylpyrrolidine-2-carboxylate (Rautela et al.,^2020^)	C_9_H_17_NO_2_	−4.4
**155**	phenylalanine (Mekam et al.,^2019^)	C_9_H_11_NO_2_	−5.3
**156**	tyrosine (Mekam et al.,^2019^)	C_9_H_11_NO_3_	−5.5
**157**	2-[[2-amino-3-(4-hydroxyphenyl)propanoyl]amino]pentanedioic acid (Mekam et al.,^2019^)	C_14_H_18_N_2_O_6_	−6.8
**158**	maleic acid (Linfang et al., 2012)	C_4_H_4_O_4_	−4.3
**159**	dehydrochebulic acid triethyl ester (Yang et al.,^2020^)	C_20_H_22_O_11_	−6.7
**160**	hydroxycitric acid (Mekam et al.,^2019^)	C_6_H_8_O_8_	−5.1
**161**	citric acid (Mekam et al.,^2019^)	C_6_H_8_O_7_	−5.1
*Organic acids and derivatives/hydroxy acids and derivatives*			
**162**	malic acid (Mekam et al.,^2019^)	C_4_H_6_O_5_	−4.8
*Organic acids and derivative/organic phosphoric acid and derivative*			
**163**	methyl bis(trimethylsilyl) phosphate (Karki et al.,^2019^)	C_7_H_21_O_4_PSi_2_	NA
**164**	1,4-digalloylquinic acid (Mahomoodally et al.,^2020^)	C_21_H_20_O_14_	−7.8
**165**	3,5-digalloylquinic acid (Mekam et al.,^2019^)	C_21_H_20_O_14_	−8.1
**166**	3-hydroxyoctanoic acid *O*-beta-d-glucopyranoside (Nomoto et al.,^2013^)	C_14_H_26_O_8_	−6.1
**167**	hirtionoside A (Nomoto et al.,^2013^)	C_26_H_34_O_12_	−8.7
**168**	hirtionoside B (Nomoto et al.,^2013^)	C_26_H_34_O_11_	−8.8
**169**	hirtionoside C (Nomoto et al.,^2013^)	C_26_H_36_O_11_	−8.4
Organohalogen compound/organobromide			
**170**	1,5-dibromo-3-methylpentane (Rautela et al.,^2020^)	C_6_H_12_Br_2_	−3.4
*Organohalogen compound/organochloride*			
**171**	1-bromo-6-chlorohexane (Rautela et al.,^2020^)	C_6_H_12_BrCl	−3.2
*Organoheterocyclic compound/benzofuran*			
**172**	3,6-dimethyl-5,6,7,7*a*-tetrahydro-4*H*-1-benzofuran-2-one (Rautela et al.,^2020^)	C_10_H_14_O_2_	−5.2
*Organoheterocyclic compound/coumaran*			
**173**	2,3-dihydrobenzofuran (Rautela et al.,^2020^)	C_8_H_8_O	−4.3
*Organoheterocyclic compound/epoxide*			
**174**	13-oxabicyclo[10.1.0]tridecane (Karki et al.,^2019^)	C_12_H_22_O	−4.7
*Organoheterocyclic compounds/indoles and derivatives*			
**175**	1,2,3,4-tetrahydrocyclopenta[*b*]indole (Rautela et al.,^2020^)	C_11_H_11_N	−5.4
**176**	tryptophan (Mekam et al.,^2019^)	C_11_H_12_N_2_O_2_	−6.1
*Organoheterocyclic compound/oxane*			
**177**	1,3,3-trimethyl-2-oxabicyclo[2.2.2]octan-6-ol (Rautela et al.,^2020^)	C_10_H_18_O_2_	−5.2
*Organoheterocyclic compound/oxepane*			
**178**	3,4-epoxycyclohexylmethyl 3,4-epoxycyclohexanecarboxylate (Karki et al.,^2019^)	C_14_H_20_O_4_	−6.2
*Organoheterocyclic compound/piperidine*			
**179**	1-(2-piperidin-4-ylethyl)pyrrolidin-2-one (Rautela et al.,^2020^)	C_11_H_20_N_2_O	−5.2
*Organoheterocyclic compound/pyran*			
**180**	3,5-dihydroxy-6-methyl-2,3-dihydropyran-4-one (Sharma et al.,^2014^; Rautela et al.,^2020^)	C_6_H_8_O_4_	−4.9
**181**	chelidonic acid (Mekam et al.,^2019^)	C_7_H_4_O_6_	−5.8
*Organoheterocyclic compounds/pyrrolidines*			
**182**	1-(3-methyl-3-butenyl)pyrrolidine (Rautela et al.,^2020^)	C_9_H_17_N	−4.1
**183**	2,2-bis(but-3-en-2-yl)pyrrolidine (Karki et al.,^2019^)	C_12_H_21_N	−4.4
**184**	1-(1-cyclohexen-1-yl)pyrrolidine (Rautela et al.,^2020^)	C_10_H_17_N	−4.6
*Organometallic compound/organometalloid compound*			
**185**	diethyl-hexoxy-(3-methylbutoxy)silane (Rautela et al.,^2020^)	C_15_H_34_O_2_Si	NA
*Organic nitrogen compound/organonitrogen compound*			
**186**	nonanenitrile (Rautela et al.,^2020^)	C_9_H_17_N	−3.8
*Organic oxygen compounds/organooxygen compounds*			
**187**	*cis*-5-*O*-(4-coumaroyl)-d-quinic acid (Mekam et al.,^2019^)	C_16_H_18_O_8_	−7.5
**188**	trigalloylquinic acid (Mekam et al.,^2019^)	C_28_H_24_O_18_	−9.0
**189**	cryptochlorogenic acid (Mekam et al.,^2019^; Mahomoodally et al.,^2020^)	C_16_H_18_O_9_	−7.2
**190**	*trans*-5-*O*-(4-coumaroyl)-d-quinic acid (Mekam et al.,^2019^)	C_16_H_18_O_8_	−7.0
**191**	chlorogenic acid (Mekam et al.,^2019^; Mahomoodally et al.,^2020^)	C_16_H_18_O_9_	−7.2
**192**	*cis*-chlorogenic acid (Mekam et al.,^2019^)	C_16_H_18_O_9_	−6.6
**193**	quinic acid (Mekam et al.,^2019^; Mahomoodally et al.,^2020^)	C_7_H_12_O_6_	−5.4
**194**	shikimic acid (Mekam et al.,^2019^)	C_7_H_10_O_5_	−5.2
**195**	[2,6,6-trimethyl-4-(3-methylbut-2-enyl)cyclohexen-1-yl]methanol (Olaoluwa et al.,^2018^)	C_15_H_26_O	−5.8
**196**	2-pentylcyclohexane-1,4-diol (Karki et al.,^2019^)	C_11_H_22_O_2_	−4.7
**197**	quercitol(Linfang et al., 2012; Shah et al.,^2019^)	C_6_H_12_O_5_	−5.4
**198**	(*R*)-lotaustralin (Nomoto et al.,^2013^)	C_11_H_19_NO_6_	−6.1
**199**	benzyl-beta-d-glucopyranoside (Nomoto et al.,^2013^)	C_13_H_18_O_6_	−6.7
**200**	rutinoside (Nomoto et al.,^2013^)	C_12_H_22_O_10_	−6.8
**201**	(2*R*,3*S*,4*S*,5*R*,6*R*)-2-(hydroxymethyl)-6-methoxyoxane-3,4,5-triol (Rautela et al.,^2020^)	C_7_H_14_O_6_	−5.3
**202**	ternatoside C (Mekam et al.,^2019^)	C_24_H_23_N_3_O_7_	−8.6
**203**	linocinnamarin (Nomoto et al.,^2013^)	C_16_H_20_O_8_	−6.5
**204**	6′-*O*-galloylsalicin (Mekam et al.,^2019^)	C_20_H_22_O_11_	−8.3
**205**	syringin (Nomoto et al.,^2013^)	C_17_H_24_O_9_	−7.0
**206**	gluconic acid (Mekam et al.,^2019^)	C_6_H_12_O_7_	−5.3
**207**	tartaric acid (Linfang et al., 2012)	C_4_H_6_O_6_	−4.8
**208**	5-hydroxymethyl-2-furancarboxaldehyde (Sharma et al.,^2014^)	C_6_H_6_O_3_	−4.4
**209**	2-hydroxy-1-(1′-pyrrolidiyl)-1-buten-3-one (Rautela et al.,^2020^)	C_8_H_13_NO_2_	−4.4
**210**	xanthoxylin (Yang et al.,^2020^)	C_10_H_12_O_4_	−5.3
**211**	megastigmatrienone A (Perumal and Mahmud, 2013)	C_13_H_18_O	−5.7
**212**	2-(4,4,4-trichlorobutyl)cyclohexan-1-one (Rautela et al.,^2020^)	C_10_H_15_Cl_3_O	−4.8
**213**	2-butoxyethanol (Ogunlesi et al.,^2009^)	C_6_H_14_O_2_	−3.7
**214**	2*E*,6*E*-dimethyl-2,6-octadiene-1,8-diol (Rautela et al.,^2020^)	C_10_H_18_O_2_	−4.8
**215**	2-methylhexadecanol (Ogunlesi et al.,^2009^)	C_17_H_36_O	−4.8
*Organic salt/organic metal salt*			
**216**	3,5-dipropyl-1,2,4,3,5-triselenadiborolane (Karki et al.,^2019^)	C_6_H_14_B_2_Se_3_	NA
*Phenylpropanoids and polyketide/cinnamic acids and derivative*			
**217**	feruloyl malate (Mekam et al.,^2019^)	C_14_H_14_O_8_	−7.0
**218**	*trans*-*para*-coumaric acid (Mekam et al.,^2019^)	C_9_H_8_O_3_	−5.1
**219**	caffeic acid (Perumal et al.,2015); Mekam et al.,^2019^)	C_9_H_8_O_4_	−5.6
**220**	ferulic acid (Mekam et al.,^2019^)	C_10_H_10_O_4_	−5.7
*Phenylpropanoids and polyketides/coumarins and derivatives*			
**221**	4-methoxyfuro[3,2-g]chromen-7-one (Rautela et al.,^2020^)	C_12_H_8_O_4_	−5.8
**222**	isopimpinellin (Rautela et al.,^2020^)	C_13_H_10_O_5_	−5.9
**223**	xanthotoxin (Rautela et al.,^2020^)	C_12_H_8_O_4_	−5.9
**224**	esculetin (Li et al.,^2015^)	C_9_H_6_O_4_	−6.2
**225**	phyllanthusiin E methyl ester (Yang et al.,^2020^)	C_14_H_10_O_8_	−7.2
**226**	phyllanthusiin E (Yang et al.,^2020^)	C_13_H_8_O_8_	−7.2
**227**	umbelliferone (Li et al.,^2015^)	C_9_H_6_O_3_	−5.5
**228**	daphnoretin Li et al. (2015)	C_19_H_12_O_7_	−8.4
**229**	scopoletin (Wu et al.,^2012^; Li et al.,^2015^; Shah et al.,^2019^)	C_10_H_8_O_4_	−5.8
**230**	isoscopoletin (Wu et al.,^2012^; Li et al.,^2015^)	C_10_H_8_O_4_	−5.7
**231**	6,7,8-trimethoxycoumarin (Sharma et al.,^2014^)	C_12_H_12_O_5_	−5.6
**232**	scoparone (Wu et al.,^2012^; Li et al.,^2015^; Shah et al.,^2019^)	C_11_H_10_O_4_	−5.7
**233**	citropten (Rautela et al.,^2020^)	C_11_H_10_O_4_	−5.7
*Phenylpropanoids and polyketides/depsides and depsidones*			
**234**	trigallic acid (Mekam et al.,^2019^)	C_21_H_14_O_13_	−9.2
**235**	digallic acid (Mekam et al.,^2019^)	C_14_H_10_O_9_	−8.3
*Phenylpropanoids and polyketides/diarylheptanoids*			
**236**	tetragalloyl glucose (Mahomoodally et al.,^2020^)	C_34_H_28_O_22_	−8.8
*Phenylpropanoids and polyketides/flavonoids*			
**237**	epicatechin 3-gallate (Perumal et al.,^2015^; Perumal et al., 2018)	C_22_H_18_O_10_	−8.2
**238**	leucocyanidol (Shah et al.,^2019^)	C_15_H_14_O_7_	−7.2
**239**	epicatechin (Mekam et al.,^2019^)	C_15_H_14_O_6_	−7.0
**240**	pinocembrin (Wu et al.,^2012^; Shah et al.,^2019^)	C_15_H_12_O_4_	−7.2
**241**	chrysin (Mekam et al.,^2019^)	C_15_H_10_O_4_	−7.3
**242**	luteolin (Wu et al.,^2012^)	C_15_H_10_O_6_	−7.5
**243**	dimethoxyquercetin (Sheliya et al.,^2015^)	C_17_H_14_O_9_	−7.3
**244**	kaempferol (Linfang et al., 2012; Wu et al.,^2012^; Rao et al.,^2017^; Ali et al.,^2020^)	C_15_H_10_O_6_	−7.8
**245**	quercetin (Liu et al.,^2007^; Linfang et al., 2012; Wu et al.,^2012^; Sheliya et al.,^2015^; Selin-Rani et al.,^2016^; Bach et al.,^2017^; Rao et al.,^2017^; Suganthi and Ravi, 2018; Mekam et al.,^2019^; Nugroho et al.,^2019^; Shah et al.,^2019^; Ali et al.,^2020^; Tayone et al.,^2020^)	C_15_H_10_O_7_	−7.5
**246**	isovitexin (Nomoto et al.,^2013^)	C_21_H_20_O_10_	−8.0
**247**	kaempferol-3-*O*-glucuronide (Mekam et al.,^2019^)	C_21_H_18_O_12_	−8.7
**248**	quercetin-3-*O*-glucuronide (Mekam et al.,^2019^)	C_21_H_18_O_13_	−8.0
**249**	euphorbianin (Shah et al.,^2019^)	C_29_H_32_O_18_	−8.2
**250**	myricetin-3-*O*-pentoside (Mekam et al.,^2019^)	C_20_H_18_O_12_	−8.4
**251**	myricetin-3-*O*-hexoside (Mekam et al.,^2019^)	C_21_H_20_O_13_	−7.3
**252**	quercetin 3-*O*-alpha-l-arabinofuranoside (Bach et al.,^2020^)	C_20_H_18_O_11_	−8.5
**253**	quercetin-3-*O*-pentoside (Mekam et al.,^2019^)	C_20_H_18_O_11_	−8.4
**254**	kaempferol-3-*O*-rhamnoside (Mekam et al.,^2019^)	C_21_H_20_O_10_	−7.7
**255**	narcissin (Mekam et al.,^2019^)	C_28_H_32_O_16_	−8.9
**256**	nicotiflorin (Mekam et al.,^2019^)	C_27_H_30_O_15_	−8.7
**257**	afzelin (Liu et al.,^2007^; Nomoto et al.,^2013^; Shah et al.,^2019^; Bach et al.,^2020^; Mahomoodally et al.,^2020^)	C_21_H_20_O_10_	−8.8
**258**	astragalin (Bach et al.,^2020^; Mahomoodally et al.,^2020^)	C_21_H_20_O_11_	−8.3
**259**	myricetin-3-*O*-rhamnoside (Liu et al.,^2007^; Linfang et al., 2012; Nugroho et al.,^2019^; Shah et al.,^2019^; Bach et al.,^2020^; Mahomoodally et al.,^2020^; Tayone et al.,^2020^)	C_21_H_20_O_12_	−9.0
**260**	isorhamnetin (Wu et al.,^2012^; Shah et al.,^2019^)	C_21_H_20_O_12_	−7.3
**261**	hyperoside (Mekam et al.,^2019^)	C_21_H_20_O_12_	−8.5
**262**	rutin (Linfang et al., 2012; Bach et al.,^2017^; Rao et al.,^2017^; Suganthi and Ravi, 2018; Mekam et al.,^2019^; Ali et al.,^2020^; Mahomoodally et al.,^2020^)	C_27_H_30_O_16_	−8.8
**263**	isoquercitrin (Mekam et al.,^2019^; Mahomoodally et al.,^2020^)	C_21_H_20_O_12_	−8.0
**264**	quercetin-3-*O*-rhamnoside (Gopi et al.,^2016^; Mekam et al.,^2019^; Mahomoodally et al.,^2020^)	C_21_H_20_O_11_	−9.0
**265**	luteolin-7-*O*-beta-d-glucopyranoside (Bach et al.,^2020^)	C_21_H_20_O_11_	−7.9
**266**	cosmosiin (Mahomoodally et al.,^2020^)	C_21_H_20_O_10_	−7.8
**267**	scutellarein 6-glucoside (Mekam et al.,^2019^)	C_21_H_20_O_11_	−7.8
**268**	hymenoxin (Bach et al.,^2020^)	C_19_H_18_O_8_	−7.0
*Phenylpropanoids and polyketides/isocoumarins and derivatives*			
**269**	brevifolin (Yang et al.,^2020^)	C_12_H_8_O_6_	−7.2
**270**	ethyl brevifolin carboxylate (Yang et al.,^2020^)	C_15_H_12_O_8_	−7.0
**271**	brevifolin carboxylic acid (Mahomoodally et al.,^2020^; Yang et al.,^2020^)	C_13_H_8_O_8_	−7.2
**272**	methyl brevifolin carboxylate (Yang et al.,^2020^)	C_14_H_10_O_8_	−6.4
*Phenylpropanoids and polyketides/tannins*			
**273**	tannic acid (Yang et al.,^2020^)	C_76_H_52_O_46_	−7.1
**274**	ellagitannin (Yang et al.,^2020^)	C_44_H_32_O_27_	−8.5
**275**	ellagic acid (Linfang et al., 2012; Mekam et al.,^2019^; Mahomoodally et al.,^2020^)	C_14_H_6_O_8_	−7.3
**276**	pedunculagin II (Mekam et al.,^2019^)	C_34_H_26_O_22_	−8.9
**277**	pedunculagin (Mekam et al.,^2019^)	C_34_H_24_O_22_	−8.0
**278**	corilagin (Mekam et al.,^2019^; Mahomoodally et al.,^2020^)	C_27_H_22_O_18_	−8.7
**279**	penta-*O*-galloylglucose (Mekam et al.,^2019^; Mahomoodally et al.,^2020^)	C_41_H_32_O_26_	−8.0
*Phenylpropanoids and polyketides*			
**280**	(*R*)-euphorhirtin H (Yang et al.,^2020^)	C_16_H_12_O_10_	−7.7
**281**	(*R*)-euphorhirtin I (Yang et al.,^2020^)	C_15_H_10_O_10_	−7.5
**282**	(*R*)-euphorhirtin J (Yang et al.,^2020^)	C_17_H_14_O_10_	−7.6
**283**	(*R*)-euphorhirtin K (Yang et al.,^2020^)	C_18_H_16_O_10_	−7.5
**284**	(*R*)-euphorhirtin L (Yang et al.,^2020^)	C_18_H_16_O_10_	−6.4
**285**	(*R*)-euphorhirtin M (Yang et al.,^2020^)	C_17_H_16_O_9_	−6.4
**286**	(*S*)-euphorhirtin H (Yang et al.,^2020^)	C_16_H_12_O_10_	−7.0
**287**	(*S*)-euphorhirtin I (Yang et al.,^2020^)	C_15_H_10_O_10_	−7.0
**288**	(*S*)-euphorhirtin J (Yang et al.,^2020^)	C_17_H_14_O_10_	−6.9
**289**	(*S*)-euphorhirtin K (Yang et al.,^2020^)	C_18_H_16_O_10_	−6.6
**290**	(*S*)-euphorhirtin L (Yang et al.,^2020^)	C_18_H_16_O_10_	−7.2
**291**	(*S*)-euphorhirtin M (Yang et al.,^2020^)	C_17_H_16_O_9_	−6.6
**292**	5-*O*-feruloylquinic acid (Mekam et al.,^2019^)	C_17_H_20_O_8_	−7.2
**293**	chebulic acid-14,15-diethyl ester (Yang et al.,^2020^)	C_18_H_20_O_11_	−6.5
**294**	euphorhirtin E (Yang et al.,^2020^)	C_18_H_18_O_11_	−6.7
**295**	euphorhirtin F (Yang et al.,^2020^)	C_18_H_20_O_11_	−6.1
**296**	euphorhirtin G (Yang et al.,^2020^)	C_15_H_12_O_8_	−6.9
**297**	euphorhirtin N (Yang et al.,^2020^)	C_20_H_21_NO_9_	−7.5
**298**	feruloylconiferin (Mekam et al.,^2019^)	C_26_H_28_O_12_	−8.5
*Negative controls*			
**N1**	anthracene	C_14_H_10_	−5.8
**N2**	naphthalene	C_10_H_8_	−4.8
**N3**	glycerol	C_3_H_8_O_3_	−3.9
**N4**	decane	C_10_H_22_	−3.7
**N5**	hexanol	C_6_H_12_O	−3.5
**N6**	benzene	C_6_H_6_	−3.3
**N7**	cyclohexane	C_6_H_12_	−3.3
**N8**	hexane	C_6_H_14_	−3.1
**N9**	ethanol	C_2_H_6_	−2.4
**N10**	water	H_2_O	−1.8
*Positive controls*			
**P1**	efonidipine	C_34_H_38_N_3O7_P	−8.2
**P2**	bedaquiline	C_32_H_31_BrN_2_O_2_	−8.0
**P3**	tideglusib	C_19_H_14_N_2_O_2_S	−7.9
**P4**	manidipine	C_35_H_38_N_4_O_6_	−7.6
**P5**	N3	C_35_H_48_N_6O8_	−7.5
**P6**	lercanidipine	C_36_H_41_N_3_O_6_	−7.4
**P7**	boceprevir	C_27_H_45_N_5_O_5_	−7.2
**P8**	shikonin	C_16_H_16_O_5_	−6.8
**P9**	ebselen	C_13_H_9_NOSe	−6.6
**P10**	carmofur	C_11_H_16_FN_3_O_3_	−6.0

Notes: a, molecular formula; b, computed BFE in kcal/mol using AutoDock Vina implemented in PyRx0.8. Phytochemicals with NA, indicated for their BFE, contain atoms that are not well parameterized for molecular docking using PyRx0.8. Benzenoids contain the benzene ring; hydrocarbons are composed of H and C atoms only; lignans contain dimeric phenylpropanoids; lipids contain isoprene moiety (terpene or terpenoids), fatty acyls, and derivatives; OADs contain the acyl group; OOCs contain oxygen atoms (e.g., alcohols and esters); OHCC, heterocyclic ring; PPPK, Ph-C3- and alternating-(C = O)-CH_2-_. * Miscellaneous groups are composed of the least abundant phytochemicals.

### 3.2 Virtual Screening Through Automated Molecular Docking

**FIGURE 1:**
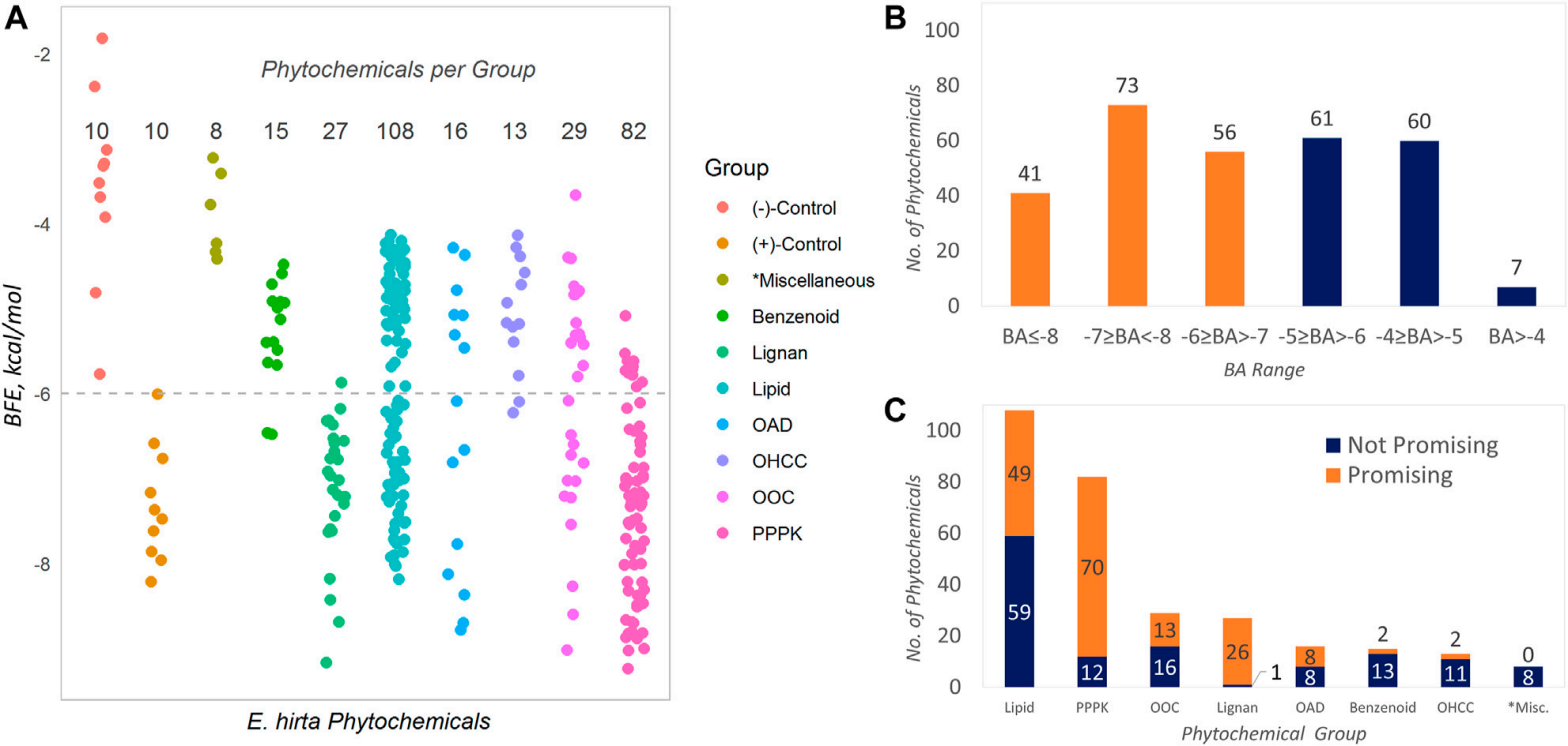
Binding properties of the phytochemicals from *E. hirta* against SARS-CoV-2 main protease. Benzenoids contain the benzene ring; hydrocarbons are composed of H and C atoms only; lignans contain dimeric phenylpropanoids; lipids contain isoprene moiety (terpene or terpenoids), fatty acyls, and derivatives; OADs contain the acyl group; OOCs contain oxygen atoms (e.g., alcohols and esters); OHCC, heterocyclic ring; PPPK, Ph-C3- and alternating—(C=O)-CH_2_-. *Miscellaneous groups are composed of the least abundant phytochemicals.

The data obtained in Table 1 are graphically presented in Figure 1. The BFE values of the phytochemicals are described in **1A**, and these are compared to the control compounds (positive and negative). It can be observed that the positive controls obtained more highly negative BFE values (thermodynamically stable receptor–ligand interaction) against SARS-CoV-2 Mpro than the negative controls (see entries in Table 1). The least negative in the group is that of carmofur with −6.0 kcal/mol computed BFE based on the AutoDock Vina docking algorithm. This value (−6.0 kcal/mol) was taken as the threshold for assigning promising inhibitors considering the fact that carmofur and the rest of the positive control compounds are actual *in vitro* inhibitors against SARS-CoV-2 Mpro. Phytochemicals having BFE values of ≤−6.0 kcal/mol qualify as promising inhibitors. In Figure 1A, these phytochemicals are represented by the points on and below the dashed horizontal line. Over this line are the non-promising inhibitors and the negative controls with less satisfactory BFE values. Overall, 170 (57.0%) of the phytochemicals found in *E. hirta* were identified as promising inhibitors against SARS-CoV-2 Mpro from a total of 298 phytochemicals.

The distribution of the BFEs is shown in Figure 1B and that of the promising inhibitors is highlighted as orange bars. The BFE range with the most abundant phytochemicals is −7 ≥ BFE < −8 with 73 (24.5%) promising inhibitors. It can be seen in both Figure 1A and Figure 1C that the two most abundant groups are lipids (108, 36.2%) and PPPKs (82, 27.5%), collectively comprising 63.7% of the total. Interestingly, PPPKs have the most number of promising phytochemicals per group. There are 70 out of 82 (85.4%) PPPKs that are promising inhibitors. This value is 23.5% of the total number of *E. hirta* phytochemicals. This behavior by the PPPKs has been previously noted (Cayona and Creencia, 2021a, Cayona and Creencia, 2022). The relative numbers of promising and non-promising inhibitors with respect to chemical grouping are given in Figure 1C.

### 3.3 Antiviral Phytochemicals From *E. hirta*

**FIGURE 2:**
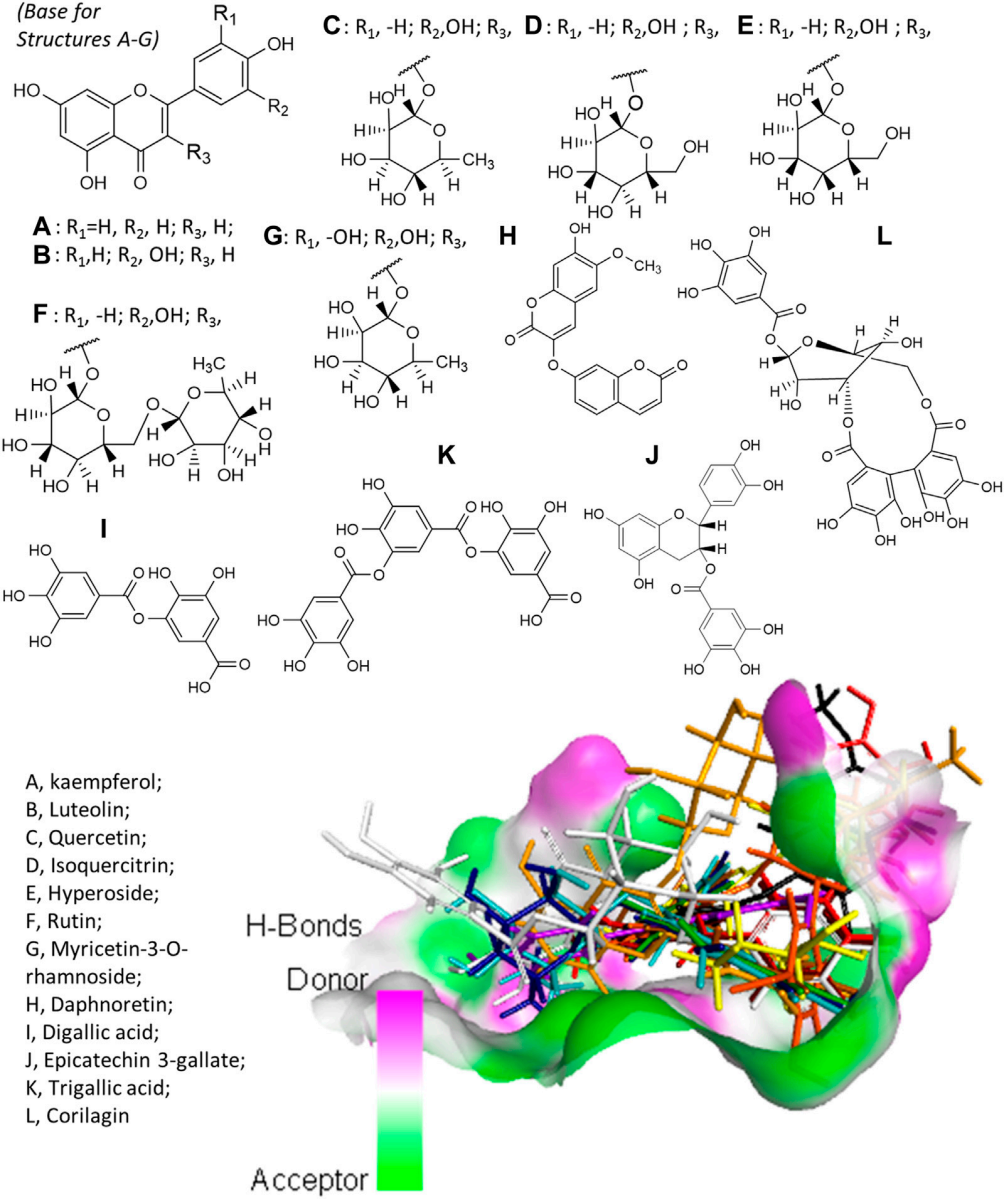
Structures of the antiviral compounds found in *E. hirta* and overlain stick representation of the ligands in complex with SARS-CoV-2 Mpro. **(A)** Brown; **(B)** purple; **(C)** mint green; **(D)** cyan; **(E)** blue; **(F)** gold; **(G)** yellow; **(H)** black; **(I)** maroon; **(J)** orange; **(K)** red; and **(L)** gray.

Virtual screening revealed that *E. hirta* is an abundant source of promising inhibitors of SARS-CoV-2 Mpro. The list of promising inhibitors includes notable compounds with interesting biological and pharmacological properties. At least 12 of the promising inhibitors were established *in vitro* or *in vivo* antiviral compounds against various viruses. These are kaempferol (A), luteolin (B), quercetin (C), isoquercitrin (D), hyperoside (E), rutin (F), myricetin-3-*O*-rhamnoside (G), daphnoretin (H), digallic acid (I), epicatechin-3-gallate (J), trigallic acid (K), and corilagin (L). The chemical structures of the aforementioned compounds and their overlain conformations on the active site of SARS-CoV-2 Mpro represented by an H-bonding surface are shown in Figure 2. A–G all have a common molecular skeleton, of which A is the only one without an attached sugar moiety. The skeleton of H is an isomer of A–G skeleton, and I–L are gallic acid derivatives.

The viruses susceptible to compounds A–L are listed in Table 2 along with relevant details obtained from virtual screening (i.e., BFEs and interacting AAs). The susceptible viruses include herpes simplex virus (HSV), hepatitis, enterovirus, human immunodeficiency virus (HIV), and influenza. Interestingly, specific antivirals are effective against viruses that affect the respiratory tract, such as CoVs and respiratory syncytial viruses (RSVs). This property is clearly relevant when considering chemical therapy against respiratory tract-related diseases like COVID-19. Kaempferol is active against CoVs (Schwarz et al., 2014), and luteolin (Wang S. et al., 2020) and daphnoretin (Wang S. et al., 2020) are active against RSVs.

**TABLE 2:** *In vitro* antiviral phytochemicals rediscovered from the medicinal plants used in this study.

***	Phytochemical	BFE^a^	Interacting AAs^b^	Susceptible viruses^c^
A	Kaempferol	−7.8	**H41**, M49, L141, **C145**, H163, E166, M165, R188	HSV-1 (Zhu et al.,^2018^); **CoV** (Schwarz et al.,^2014^)
B	Luteolin	−7.5	N142, **C145**, M165, R188, T190	**RSV** (Wang S et al.,2020); HSV (Béládi et al.,^1977^; Fan et al.,^2016^; Xu et al.,^2000^)
C	Quercetin	−7.5	M49, L141, **C145**, M165, E166, Q189	HSVs (Gaudry et al.,^2018^; Kim et al.,^2020^; Xu et al.,^2000^)
D	Isoquercitrin	−8.0	**H41**, M49, L141, **C145**, M165, E166, P168, D187, Q189, T190	HSV (Gaudry et al.,^2018^; Kim et al.,^2020^; Xu et al.,^2000^)
E	Hyperoside	−8.5	M49, L141, **C145**, M165, E166, R188, Q189, T190, Q192	Hepa B (Wu et al.,2007)
F	Rutin	−8.8	T26, L141, N142, G143, **C145**, H163, M165, E166, R188, Q189, T190	HSVs (Béládi et al.,^1977^); HIV-1 (Xu et al.,^2000^); enterovirus (Lin et al.,^2012^)
G	Myricetin-3-*O*-rhamnoside	−9.0	M49, L141, N142, S144, **C145**, E166	Hepa B (Parvez et al.,^2020^); influenza A (Motlhatlego et al.,^2021^); HIV-1 (Ortega et al.,^2017^)
H	Daphnoretin	−8.4	**H41,** G143, **C145,** M165	**RSV** (Ho et al.,^2010^; Hu et al.,^2000^)
I	Digallic acid	−8.3	L141, G143, S144, **C145**, H163, H164, M165, E166, R188	HIV (Nakane et al.,^1990^)
J	Epicatechin 3-gallate	−8.2	**H41**, F140, L141, N142, **C145**, M165, E166, H172	HSV-2 (Alvarez et al.,^2012^)
K	Trigallic acid	−9.2	T26, L141, G143, S144, **C145**, M165, E166, H163, Q189	HIV (Nakane et al.,^1990^)
***L	Corilagin	−8.7	L141, N142, G143, S144, **C145**, H163, E166, P168, T190, Q192	HSV-1 (Guo et al.,^2015^); Hepa C (Reddy et al.,^2018^)

Notes: a*,* computed binding affinity towards SARS-CoV-2, Mpro in kcal/mol; b, interacting AA residues of the most stable conformation of the docked ligands; c, based on reported *in vitro* antiviral activity (HSV, herpes simplex virus; RSV, respiratory syncytial virus; HIV, human immunodeficiency virus; Hepa, hepatitis).

**FIGURE 3:**
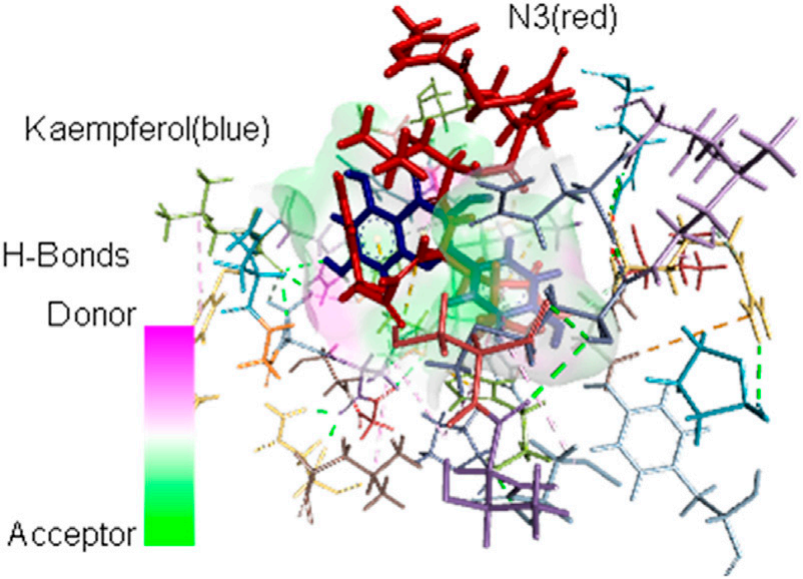
Most stable conformations of the docked kaempferol (blue) and the inhibitor N3 (red) into the active site of SARS-CoV-2 Mpro showing the AA residues near the ligands.

The interacting AAs are obtained from the most stable molecular docking conformation. These AAs are located at least 3.5 Å from the nearest atom of the docked ligands. It can be observed in Table 2 that H41 and/or C145 (in boldface) catalytic dyad residues in the active site of SARS-CoV-2 Mpro can interact with the promising inhibitors (identified using DSV 2020); however, molecular dynamics (MD) simulations are necessary to assess the stability of the receptor–ligand complex that can be formed. As stated previously, MD simulations are not covered in the scope of the present study and are reserved for future analyses. Nevertheless, the identification of these dyad residues in close proximity to the docked ligands provides a rationale for further studies. Figure 3 shows how the most stable docked conformations of kaempferol (one of the promising inhibitors) and N3 (known inhibitor) fit into the active site of SARS-CoV-2 Mpro. The AAs that are in close proximity to the ligands are also shown.

## 4 Discussion

This study provides the most comprehensive phytochemical gathering for *E. hirta* at present. It is argued in this the study that organized phytochemical composition will generate new information and enable meaningful analyses that may aid in understanding phytochemistry and plant metabolism. It was quite unexpected to discover an abundant cocktail of potential SARS-CoV-2 Mpro inhibitors from a single plant species. Clearly, lipids and PPPKs are among the most diverse groups of phytochemicals in *E. hirta*. These groups are also observed to be significantly more abundant in quantity than other phytochemical groups in *E. hirta* extracts (Sharma et al., 2014; Rao et al., 2017).

The molecular surface representation of the receptor reveals abundant hydrogen donor (purple) and acceptor (green) sites. This partly explains the observation that the ligands that can effectively establish H-bonding generally possess more negative BFEs than those that do not. Careful examination of the individual structures of the phytochemicals tested revealed that the capacity for H-bonding signifies direct correlation to favorable BFE. The ligands represented by the points below the dashed horizontal line in Figure 1A can H-bond more effectively and have more negative BFE towards SARS-CoV-2 Mpro than the ones above the line. Hydrocarbons (*Miscellaneous group), benzenoids, and OHCCs are obviously represented in the latter because they cannot (or can poorly) establish H-bonding with the receptor.

The molecular docking behavior of most PPPKs is interesting and deserves further investigation. Their docked conformations like the one presented in Figure 2 indicate the molecular skeleton that deeply buried and extended through the active site cavity of SARS-CoV-2 Mpro. The phenylpropanoids and their structurally related groups, the lignans, may feature pharmacophoric moieties.

Available preclinical information conclusively reveals that *E. hirta* possesses antiviral properties (Perera et al., 2018). In this study, some of these antiviral phytochemicals with established antiviral properties against various viruses, including those that affect the respiratory tract, were rediscovered through the PM-VS strategy. These properties are relevant in the effort to address a respiratory disease like COVID-19. More importantly, the strategy allowed the identification of many other promising inhibitors of SARS-CoV-2 Mpro despite its simplicity. Further studies are definitely necessary, but preliminary results gathered on the demonstration of the proof-of-principle for PM-VS provide a basis for exhaustive *in silico* investigations and future *in vitro* experiments. PM-VS can be efficiently implemented in the preliminary stages of drug discovery and development with minimal computational cost. Moving forward, other drug targets, not only COVID-19 drug targets, can also be investigated with PM-VS using different medicinal plants.

## 5 Conclusion

A method described as phytochemical mining allowed the systematic collection and organization of phytochemical components from *E. hirta*. A total of 298 *E. hirta* phytochemicals collected from the literature represent the most comprehensive phytochemical data collection for the plant. Virtual screening through molecular docking of the phytochemicals revealed an abundant cocktail of 170 promising inhibitors against SARS-CoV-2 Mpro. Twelve of the promising inhibitors are also prominent natural products with reported antiviral property against diverse viruses including respiratory CoV and RSVs. Finally, PM-VS was successfully implemented in this study, and the preliminary results obtained so far suggest further investigations.

## Data Availability

The raw data supporting the conclusion of this article will be made available by the authors, without undue reservation.
