# Supplementary material for: Phytochemicals of Euphorbia hirta L. and Their Inhibitory Potential Against SARS-CoV-2 Main Protease
Source: Front Mol Biosci. 2022 Feb 4;8:801401. doi: 10.3389/fmolb.2021.801401 (PMC8855059; doi:10.3389/fmolb.2021.801401)

SUPPLEMENTARY MATERIALS

Chemical Structures of Phytochemicals Gathered from *Euphorbia hirta* L.

1-14 are Benzenoids

(1-10 are Benzene and substituted derivatives)

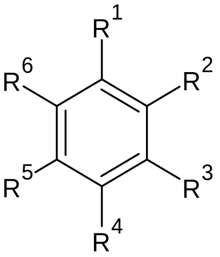

	**R¹**	**R²**	**R³**	**R⁴**	**R⁵**	**R⁶**
**1**	$-NH_2$	-H	$-CH(OH)CH_3$	-H	-H	-H
**2**	$-COO(CH_2)_{13}CH_3$	$-COO(CH_2)_2CH_3$	-H	-H	-H	-H
**3**	-COOH		-H	-H	-H	-H
**4**	-F	-CON(Et)(n-But)	-H	-H	-H	-H
**5**	-COOH	-H	-OH	-OH	-OH	-H
**6**	-COOEt	-H	-OH	-OH	-OH	-H
**7**	-COOMe	-H	-OH	-OH	-OH	-H
**8**	-COOH	-H	-OH	-OH	-H	-H
**9**	$-CH_2COCH_3$	-H	-OEt	-H	-H	-H
**10**	$-CH_2CH_2COOMe$	-H	-*t*-But	-OH	-*t*-But	-H

(11 is a Naphthalene)

11) 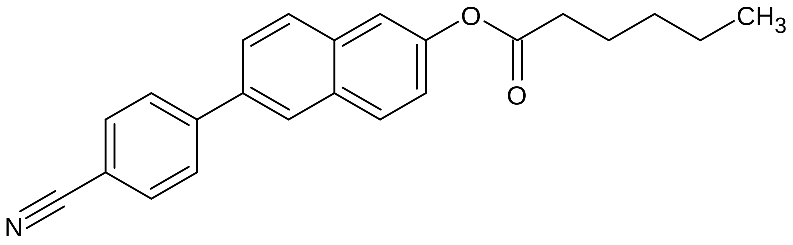

(12-14 are Phenols)

12 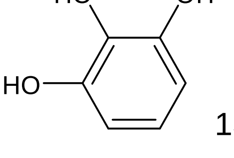

13 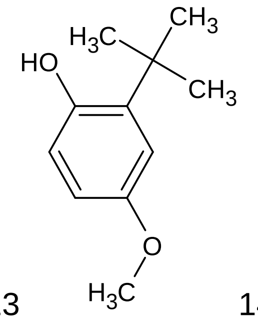

14 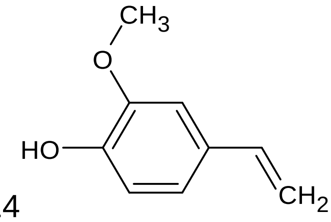

15 is a Benzenoid

15) 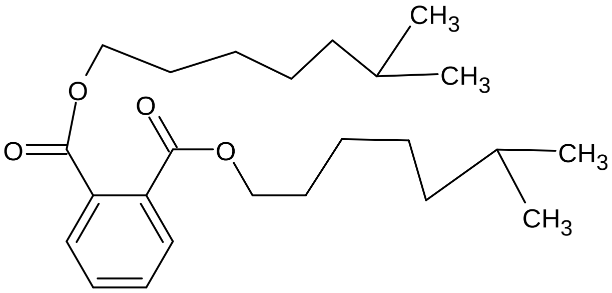

15-16 are Hydrocarbons

16 is a Saturated hydrocarbon, $C_{14}H_{30}$

17 is an Unsaturated hydrocarbon, 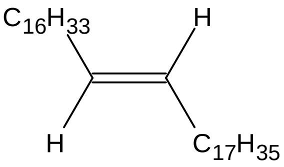

17-30 are Lignans, neolignans and related compounds

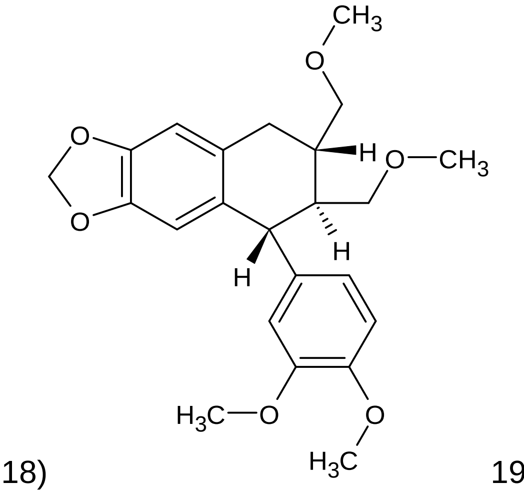

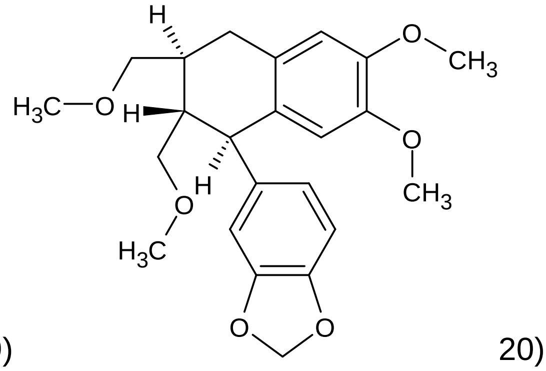

18) 19) 20)

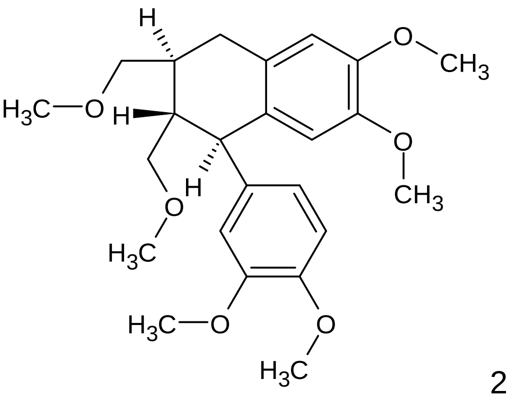

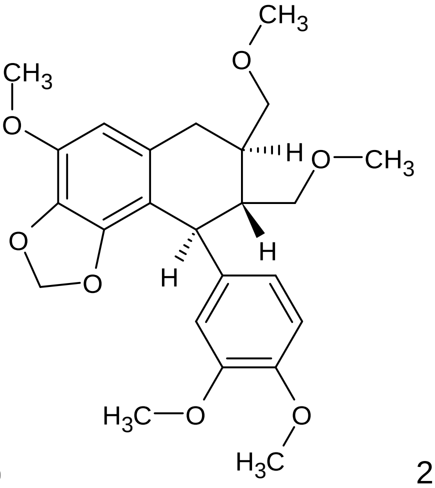

21) 22)

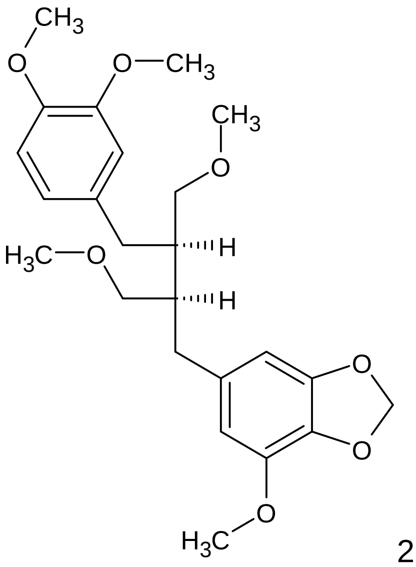

23)

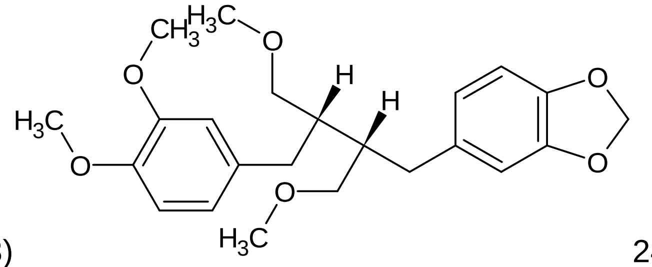

24)

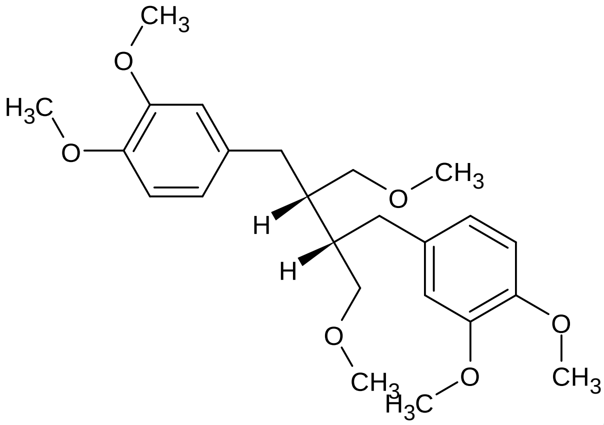

25)

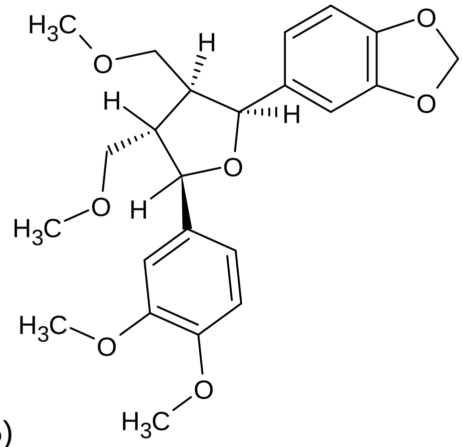

26)

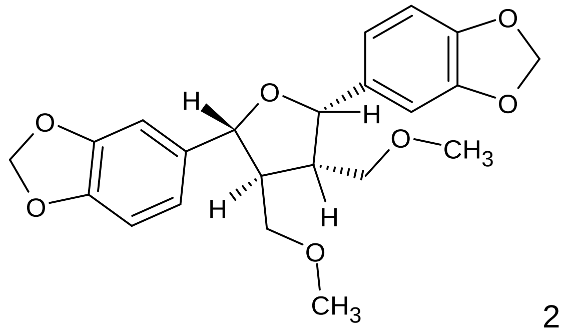

27)

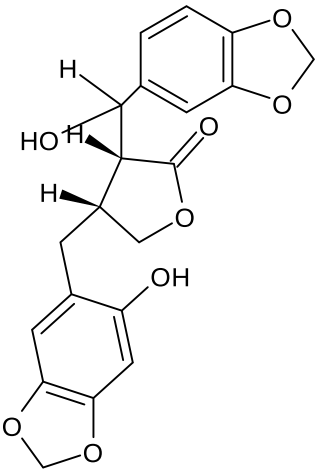

28)

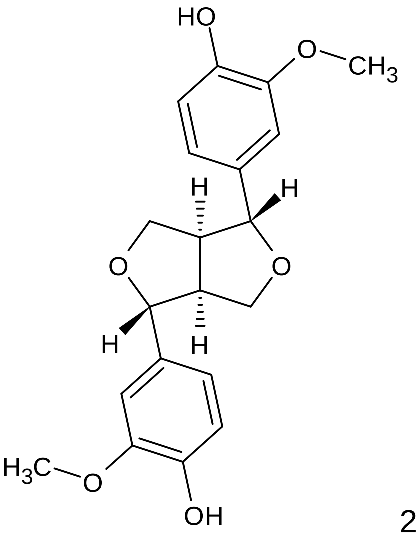

29)

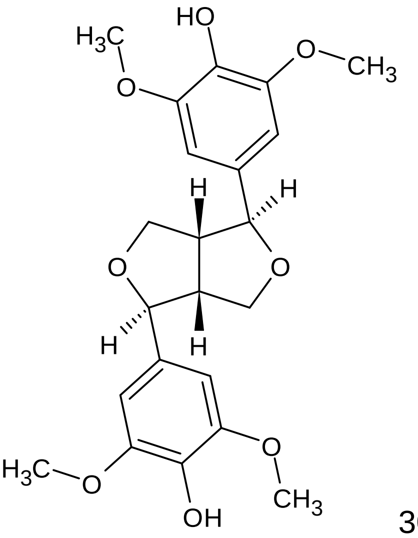

30)

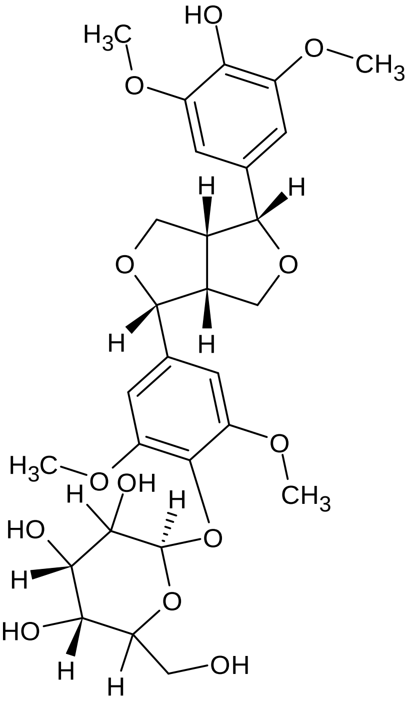

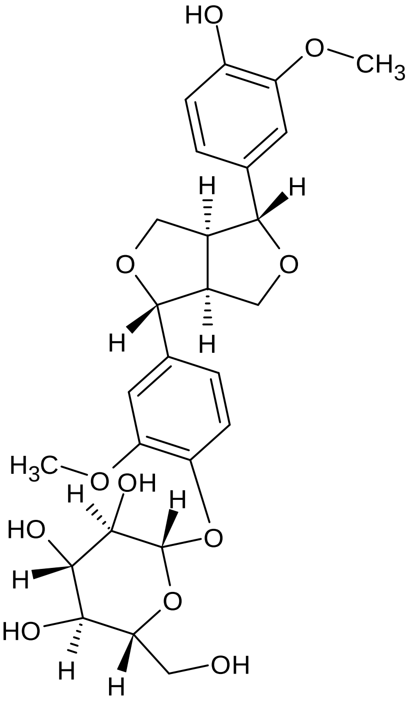

31)

32)

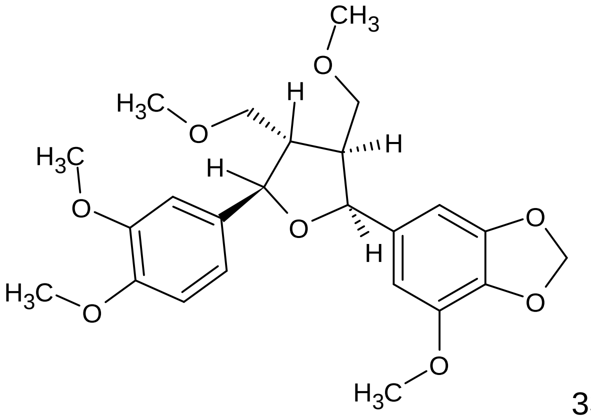

33)

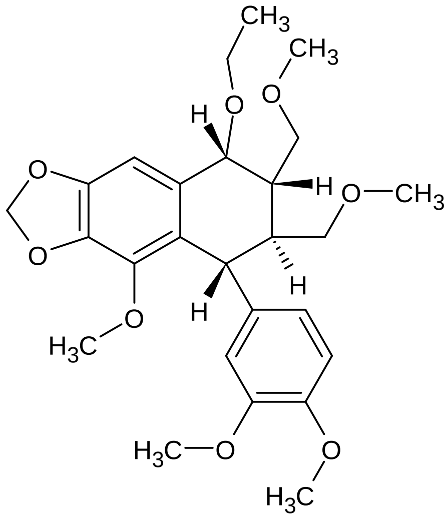

34)

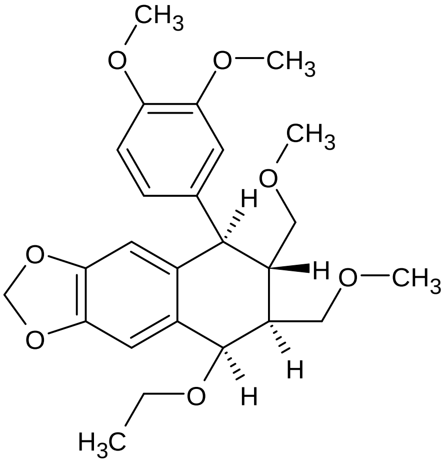

35)

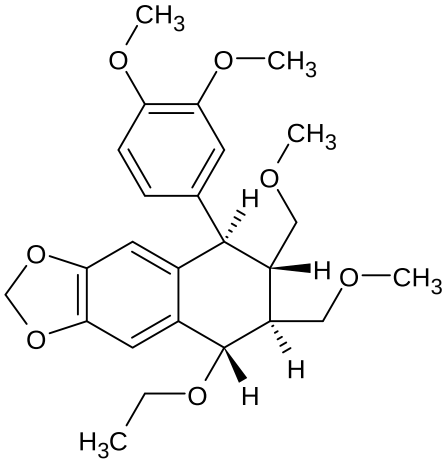

36)

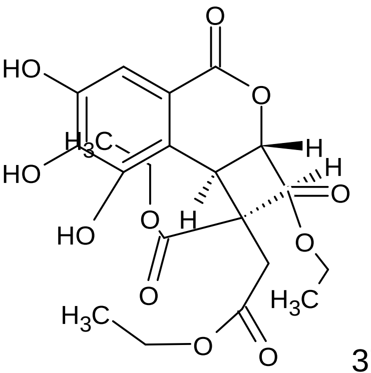

37)

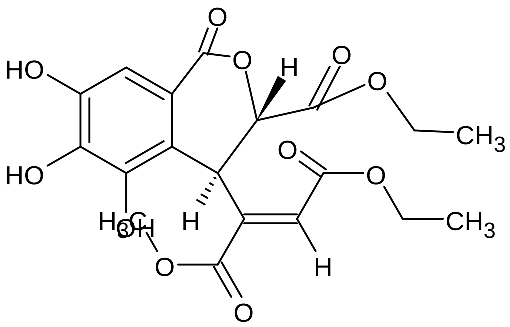

38)

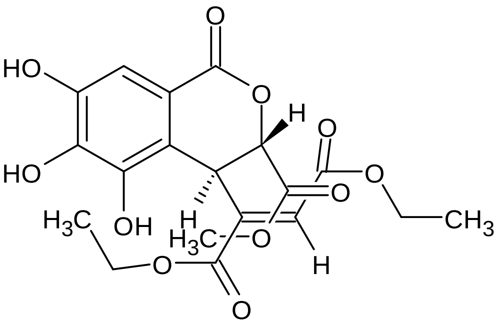

39)

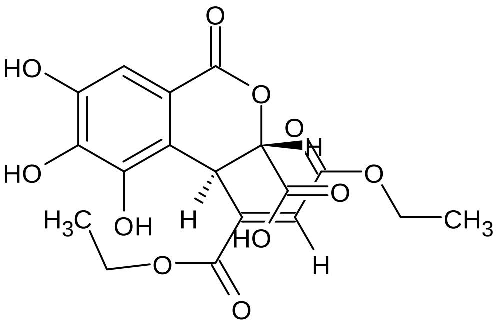

40)

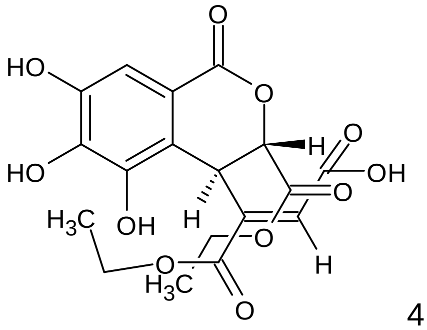

41)

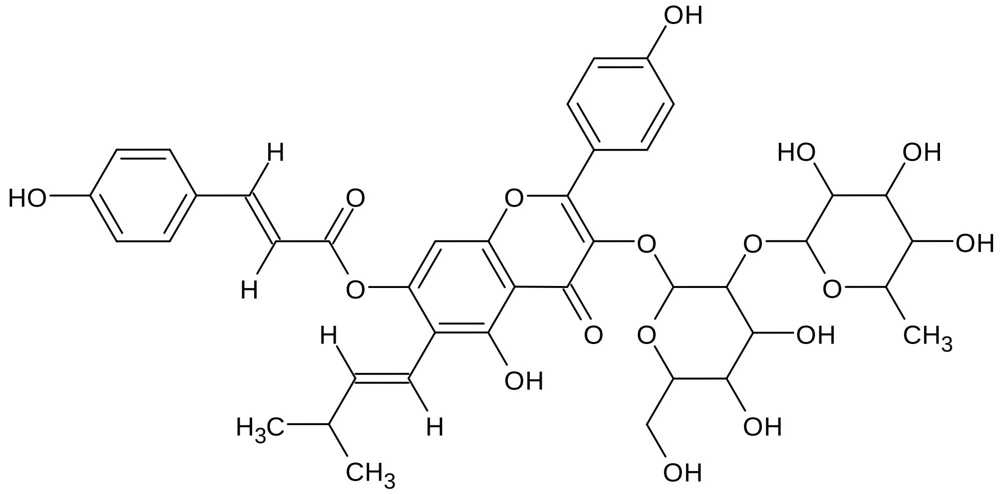

42)

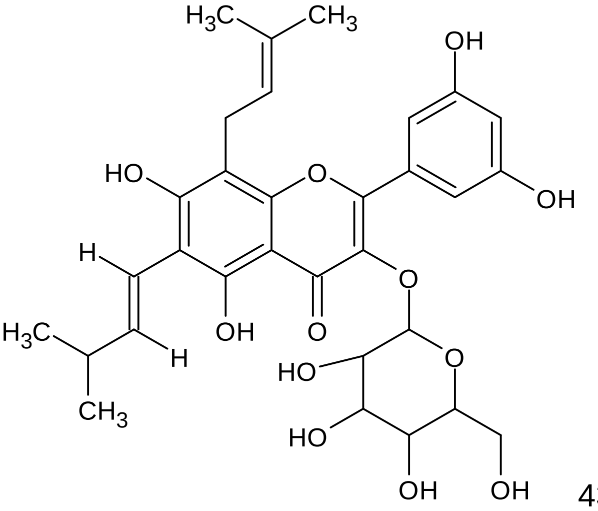

43)

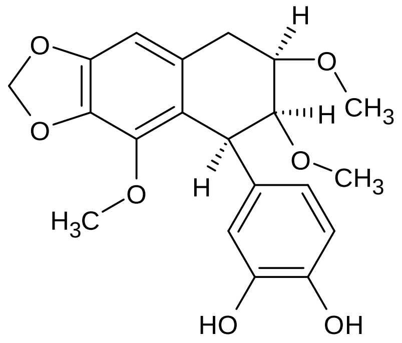

44)

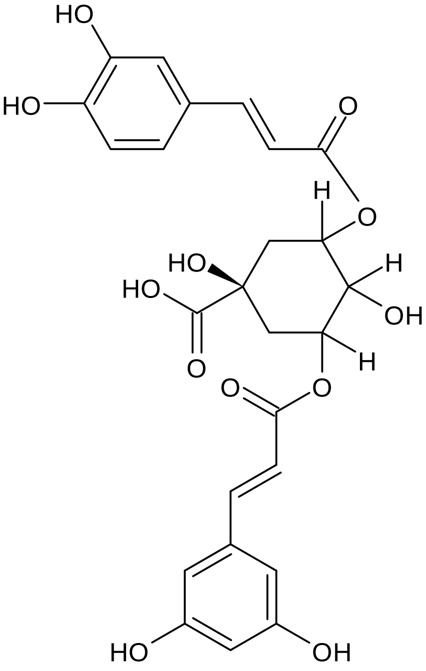

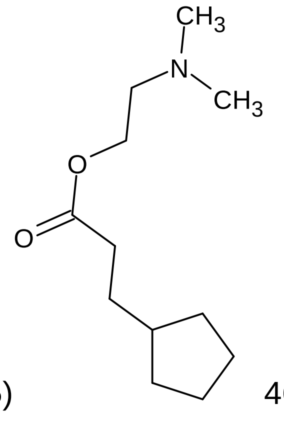

45)

46) *cis*-$CH_3(CH_2)_{16}CH_2O(CH_2)_3OCO(CH_2)_7CH{=}CH(CH_2)_7CH_3$

47) $CH_3(CH_2)_{14}COOEt$ 48) $CH_3(CH_2)_{16}COOEt$

49) 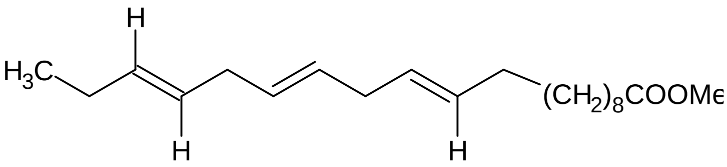

50) trans-$CH_3(CH_2)_7CH{=}CH(CH_2)_7COOMe$ 51) $CH_3(CH_2)_{14}COOMe$

52) cis-$(CH_3)_2C{=}CHCH_2CH_2CH(CH_3)CH_2CH_2OOC(CH_2)_7CH{=}CH(CH_2)_5CH_3$

53) 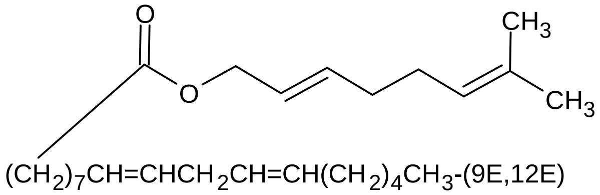

54) cis-$CH_3(CH_2)_{14}COOCH{=}CH(CH_3)(CH_2)_2CH{=}C(CH_3)_2$

55) cis-$CH_3(CH_2)_7CH{=}CH(CH_2)_7COOH$

56) $CH_3(CH_2)_{13}COOH$

57) $CH_3(CH_2)_{12}COOH$

58) $CH_3(CH_2)_{14}COOH$

59) 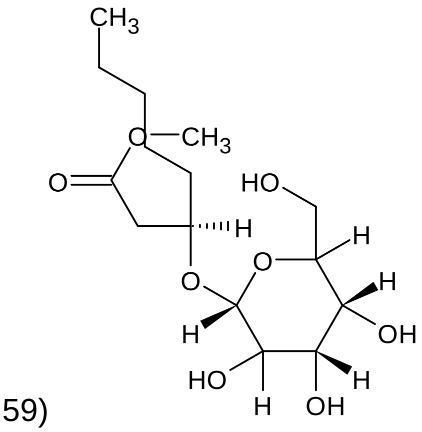

60) 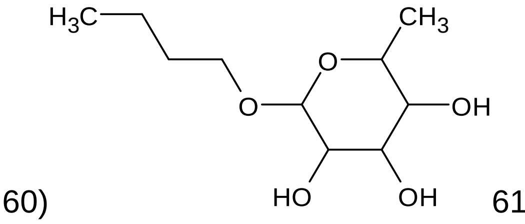

61) 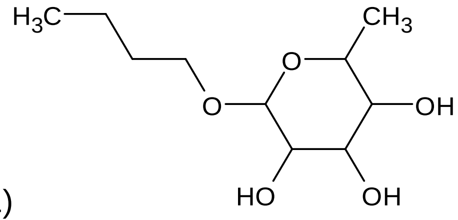

62) 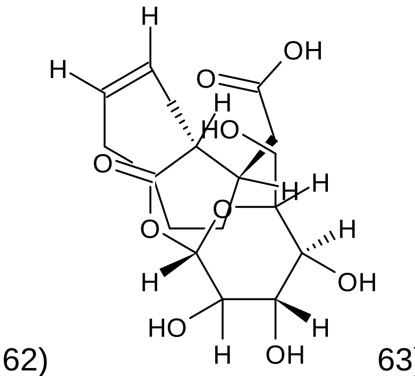

63) 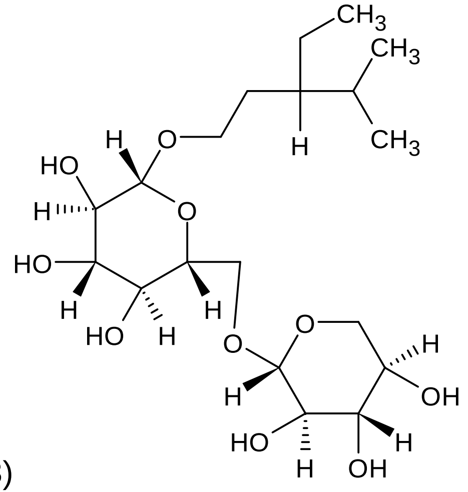

64) 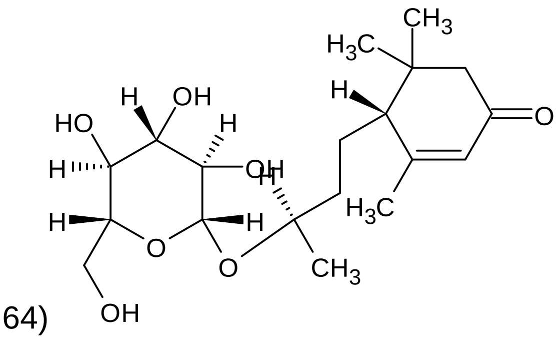

65) 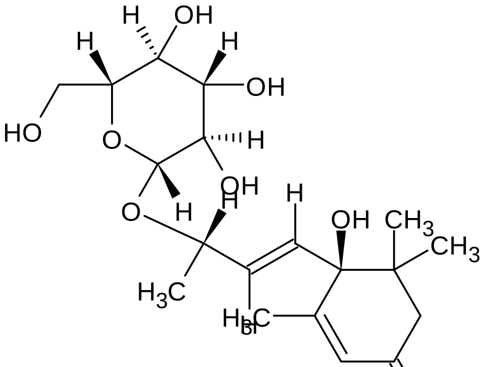

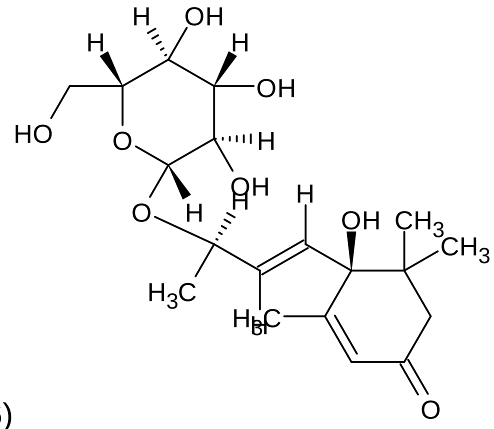

66)

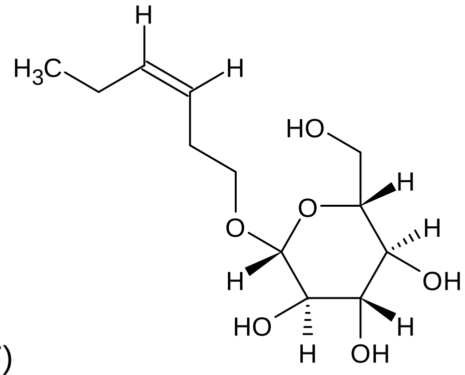

67)

68) trans-$(CH_3)C{=}CHCH_2CH_2C(CH_3){=}CHCH_2OAc$

69) cis-$(CH_3)C{=}CHCH_2CH_2C(CH_3){=}CHCH_2OAc$

70) (9E,12E,15E)-
$CH_3CH_2CH{=}CHCH_2CH{=}CHCH_2CH{=}CH(CH_2)_7CH_2OH$

71) $CH_3(CH_2)_2C{\equiv}C(CH_2)_{11}CH_2OH$

72) cis-$CH_3(CH_2)_3CH{=}CH(CH_2)_{11}CHO$

73) cis-$CH_3(CH_2)_3CH{=}CH(CH_2)_7CHO$

74) $CH_3(CH_2)_{14}CHO$

75) cis-$CH_3(CH_2)_8CH{=}CH(CH_2)_7C{=}NH(OH)$

76) cis-$CH_3(CH_2)_{12}C{=}NH(OH)$

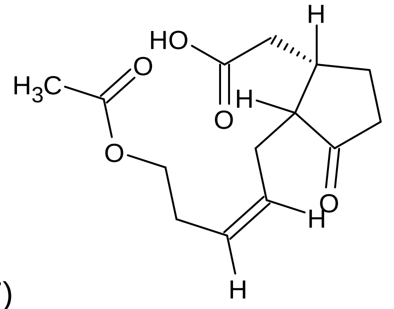

77)

78) (9Z,12Z,15Z)-
$CH_3CH_2CH=CHCH_2CH=CHCH_2CH=CH(CH_2)_7COOMe$

79) (9Z,12Z)-$CH_3(CH_2)_4CH=CHCH_2CH=CH(CH_2)_7COOMe$

80) (9Z,12Z)-$CH_3(CH_2)_4CH=CHCH_2CH=CH(CH_2)_7COOCH_2CH(OH)CH_2OH$

81) (9Z,12Z)-$CH_3(CH_2)_4CH=CHCH_2CH=CH(CH_2)_7COOEt$

82) (9Z,12Z,15Z)-$CH_3CH_2CH=CHCH_2CH=CHCH_2CH=CH(CH_2)_7COOH$

83) (9Z,12Z)-$CH_3(CH_2)_4CH=CHCH_2CH=CH(CH_2)_7COOH$

84) $CH_3(CH_2)_{16}COOCH_2CH(OH)CH_2OH$

85) $CH_3(CH_2)_{14}COOCH_2CH(OH)CH_2OH$

86) $CH_3(CH_2)_{16}COOCH_2CH(OH)CH_2OH$

87) $ROCH_2CH(OR)CH_2OR$; cis-[$CH_3(CH_2)_7CH=CH(CH_2)_7CO$-, R

88) $(CH_3)_2CH(CH_2)_3CH(CH_3)(CH_2)_3CH(CH_3)(CH_2)_3C(CH_3)=CHCH_2OH$

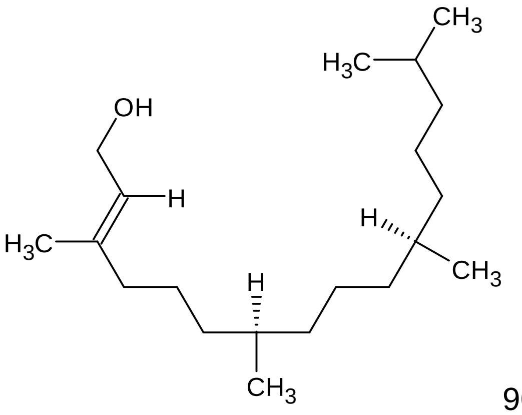

89)

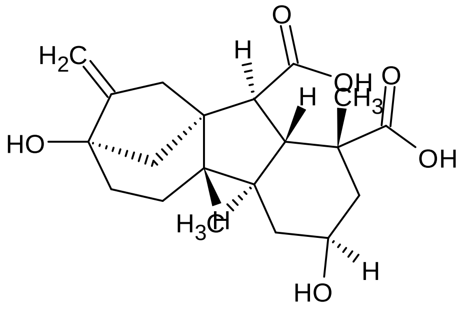

90)

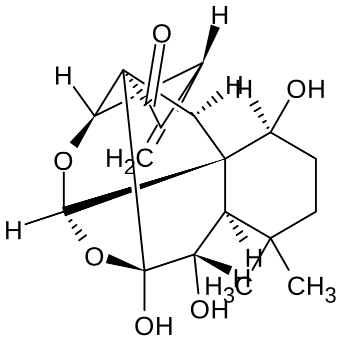

91)

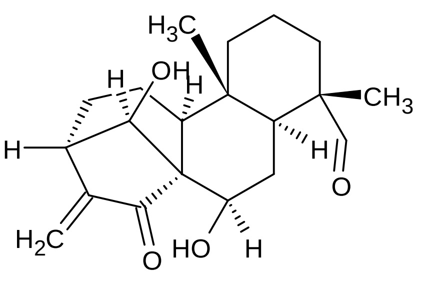

92)

93) 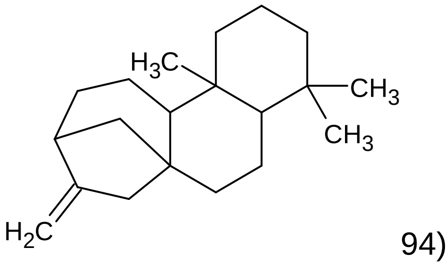

94) 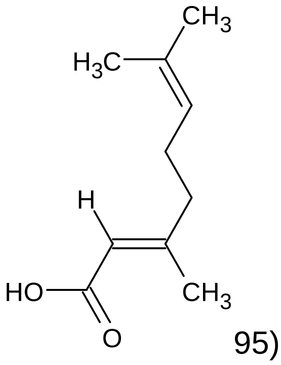

95) 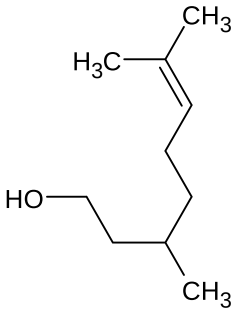

96) 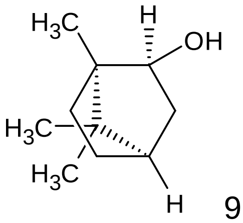

97) 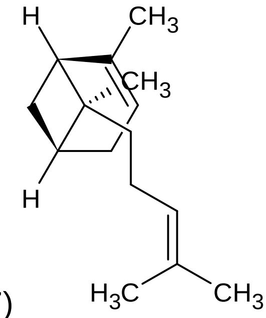

98) 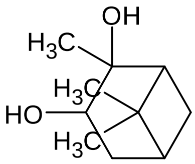

99) 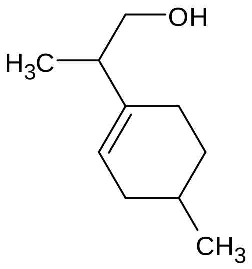

100) 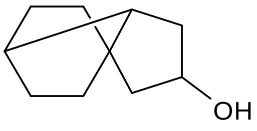

101) 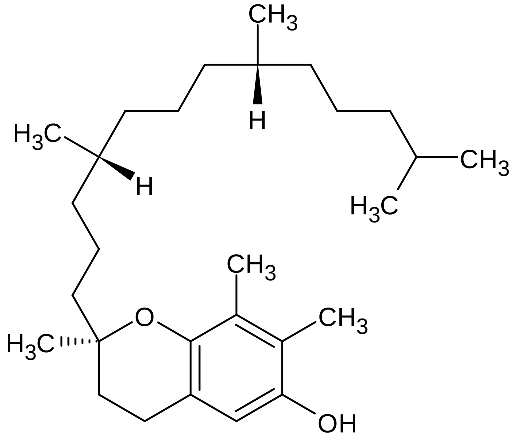

102) 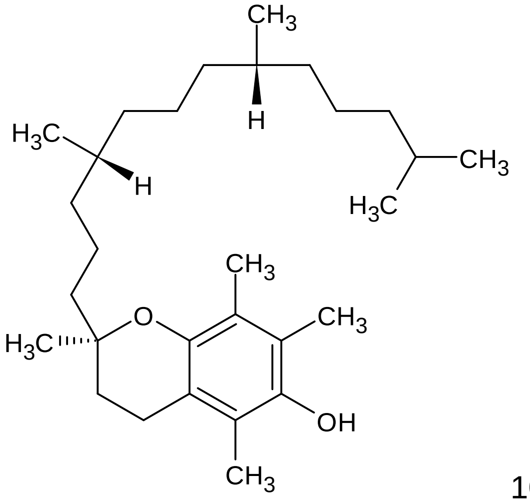

103) 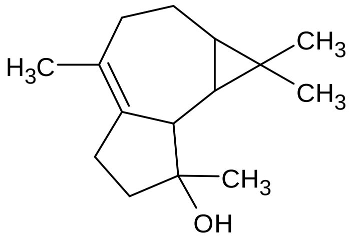

104) 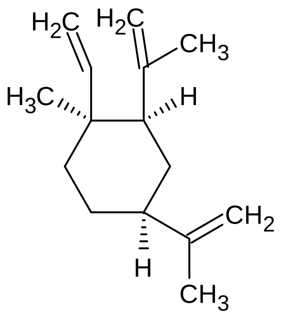

105) 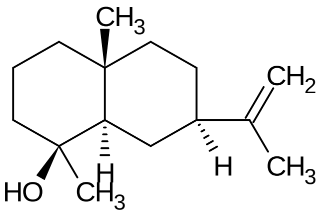

106) 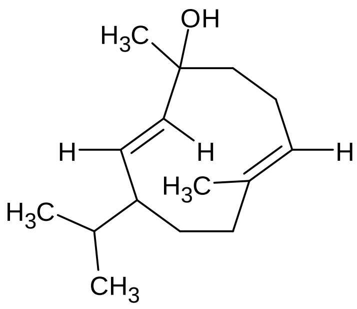

107) 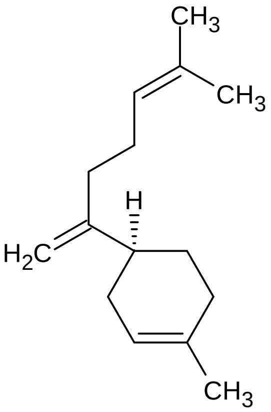

108) 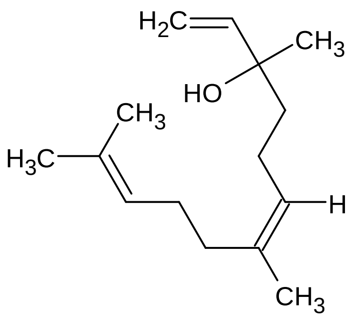

109) 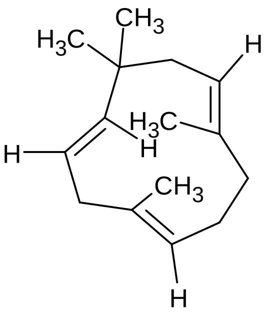

110) 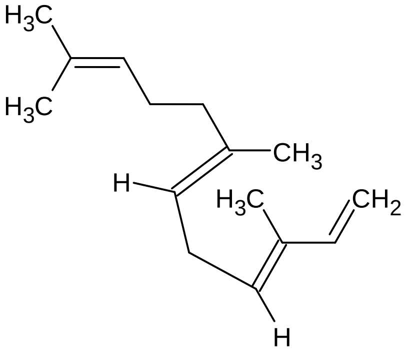

111) 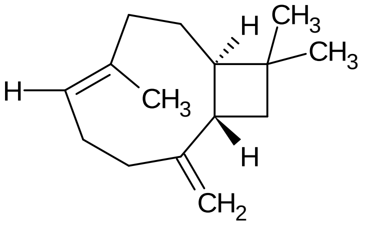

112) 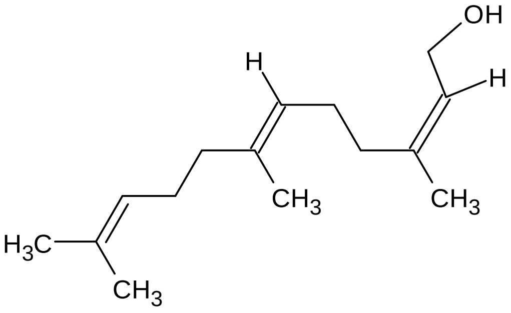

113) $(CH_3)_2CH(CH_2)_3CH(CH_3)(CH_2)_3CH(CH_3)(CH_2)_3CH_3$

114) $(CH_3)_2CH(CH_2)_3CH(CH_3)(CH_2)_3CH(CH_3)(CH_2)_3C(=CH_2)CH=CH_2$

115) $(CH_3)_2CH(CH_2)_3CH(CH_3)(CH_2)_3CH(CH_3)(CH_2)_3C(=o)CH_3$

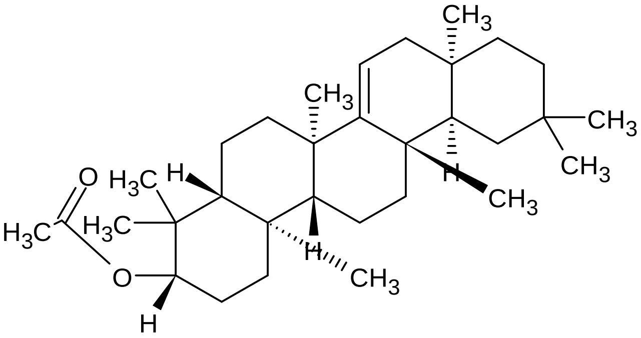

116)

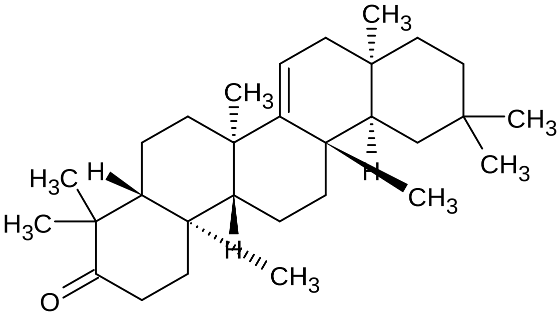

117)

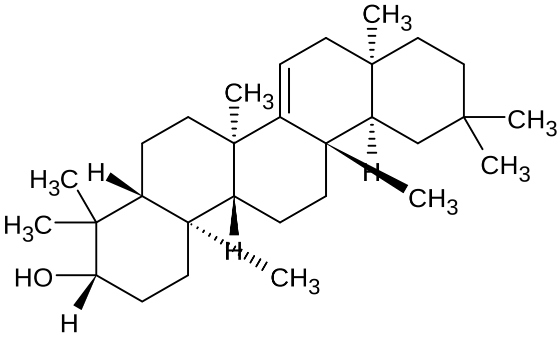

118)

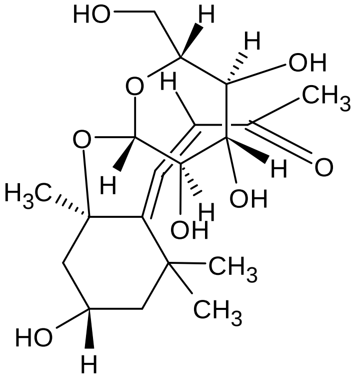

119)

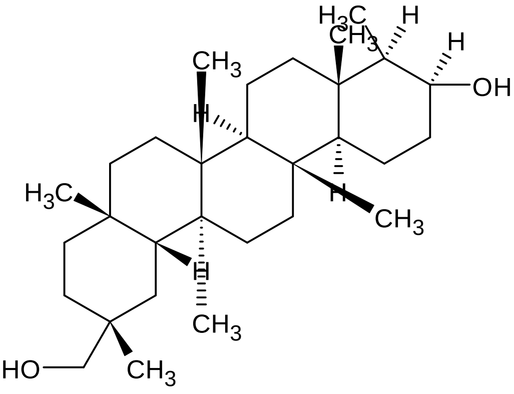

120)

121)

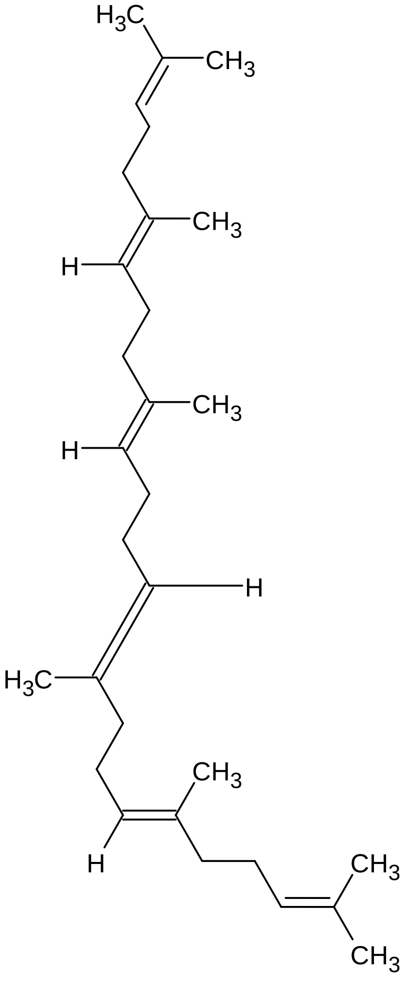

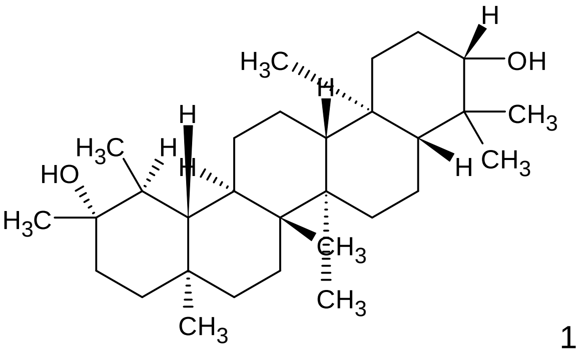

122)

123)

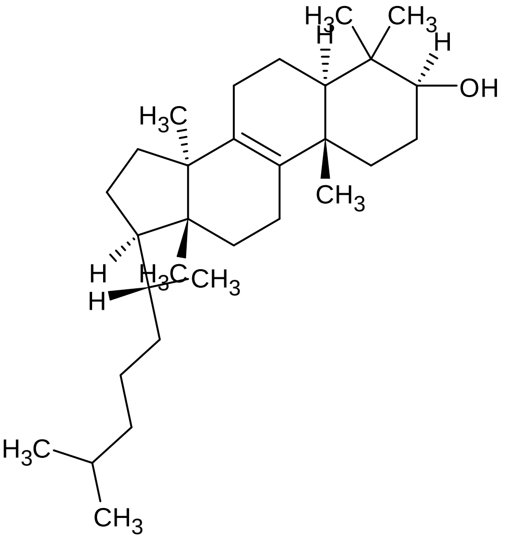

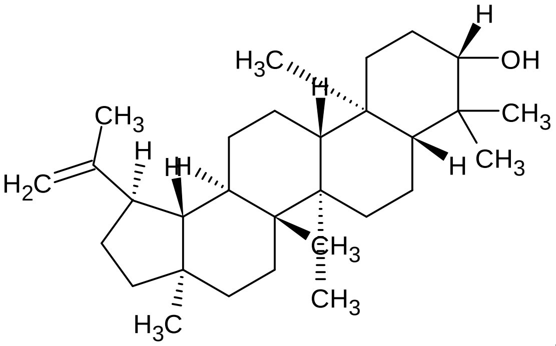

124)

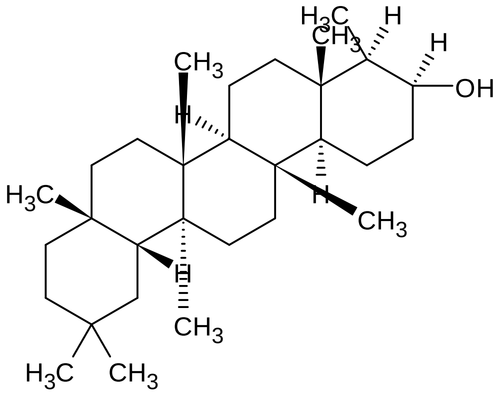

125)

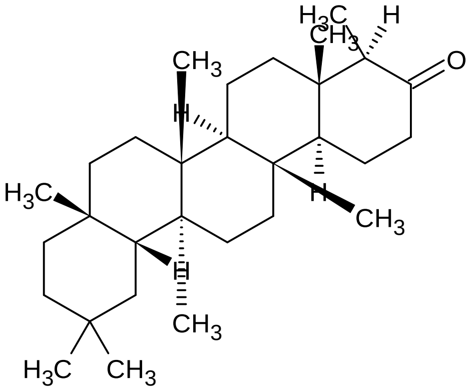

126)

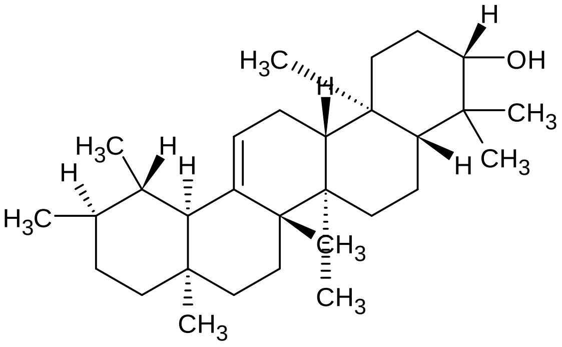

127)

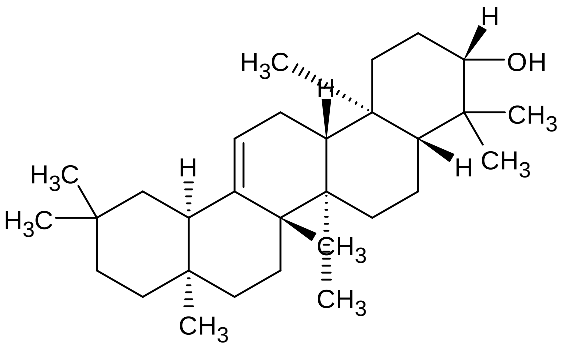

128)

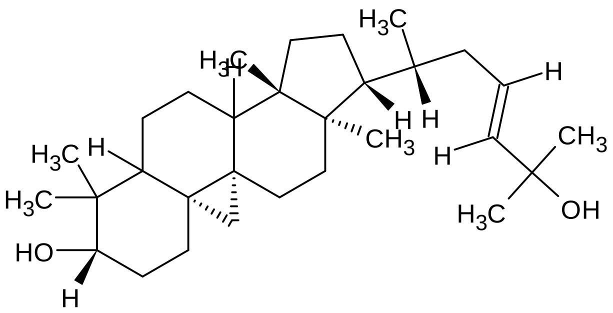

129)

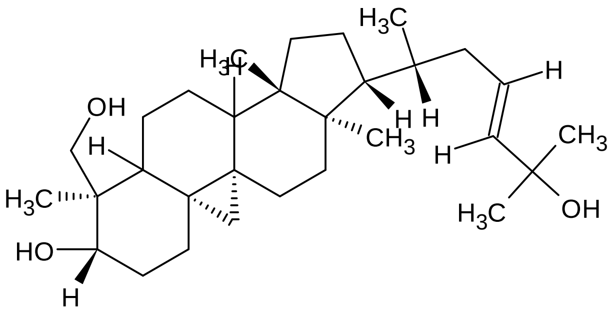

130)

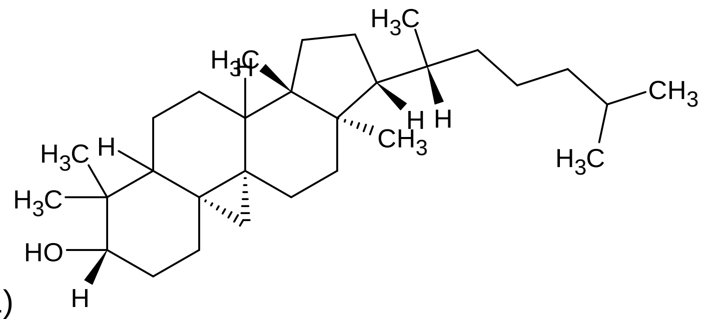

131)

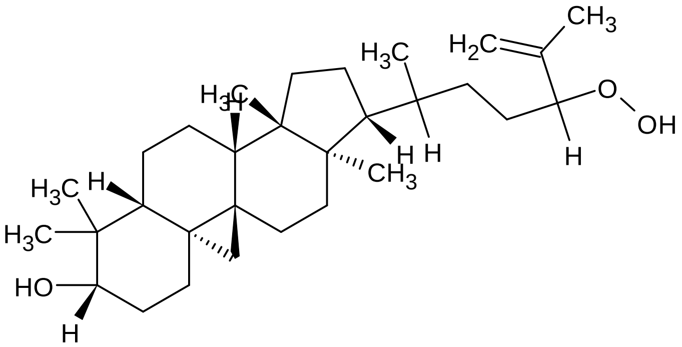

132)

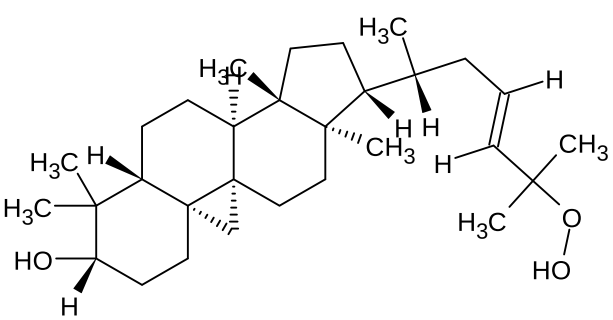

133)

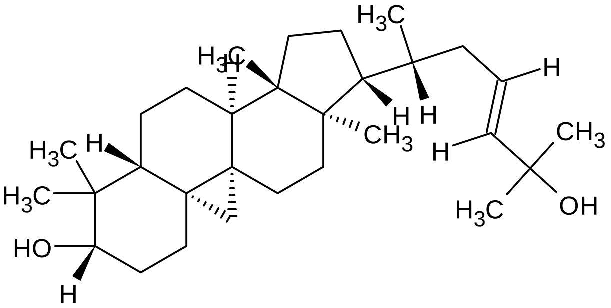

134)

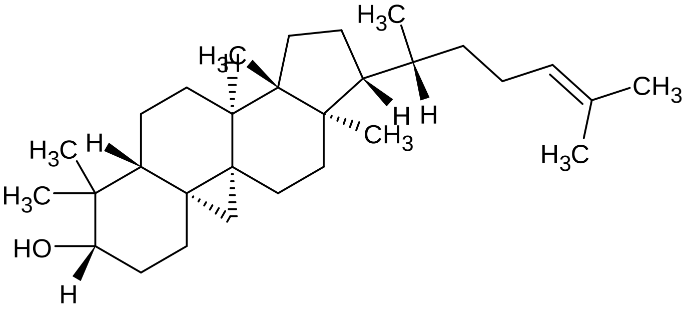

135)

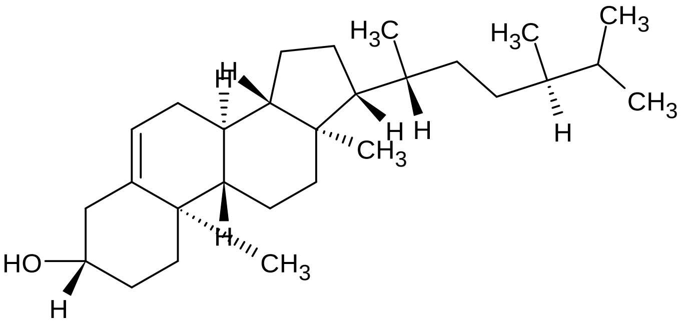

136)

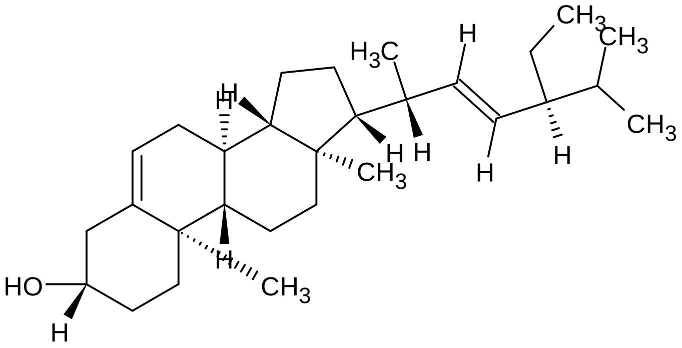

137)

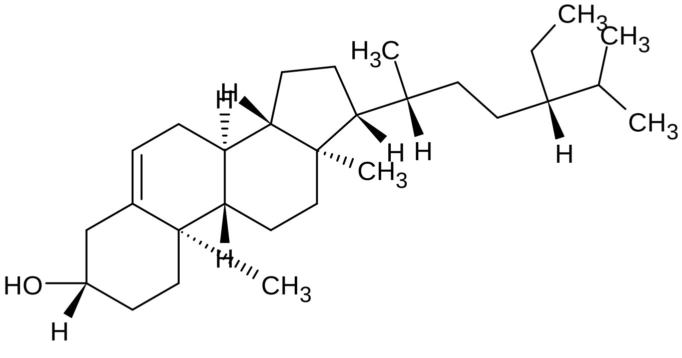

138)

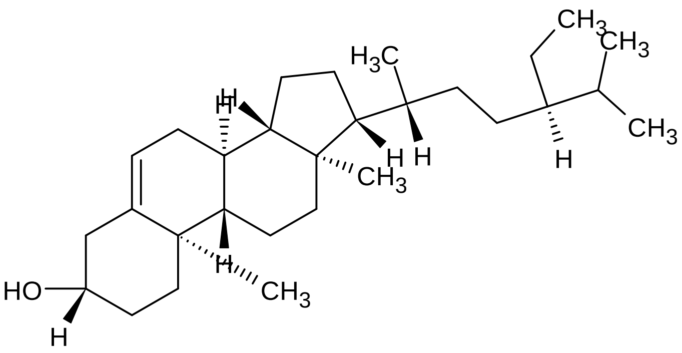

139)

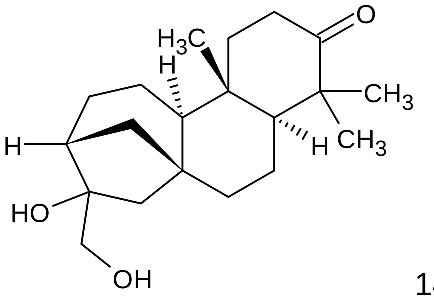

140)

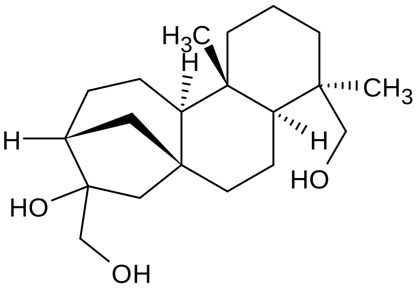

141)

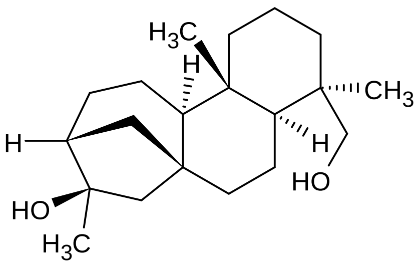

142)

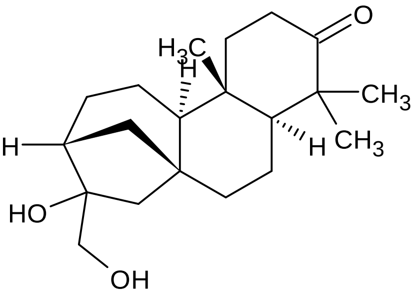

143)

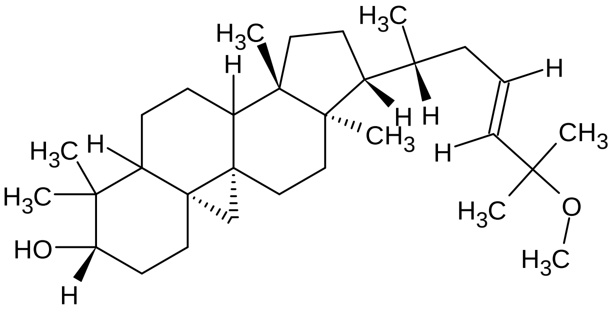

144)

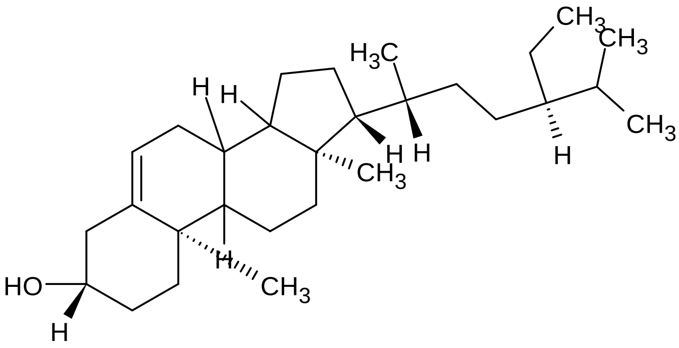

145)

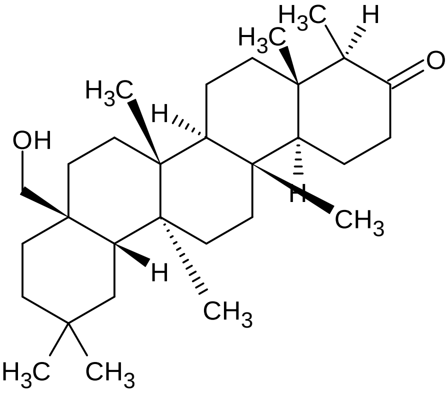

146)

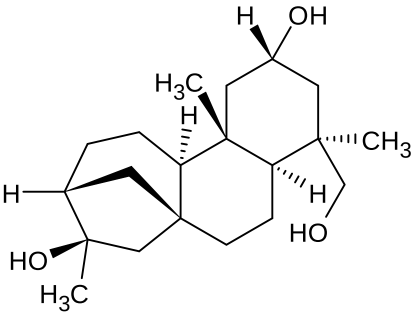

147)

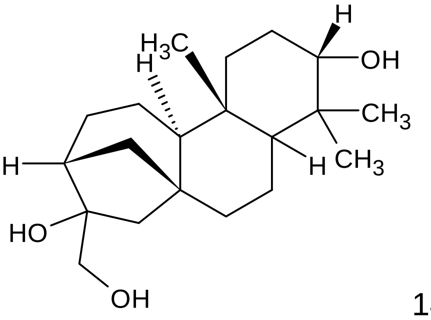

148)

149)

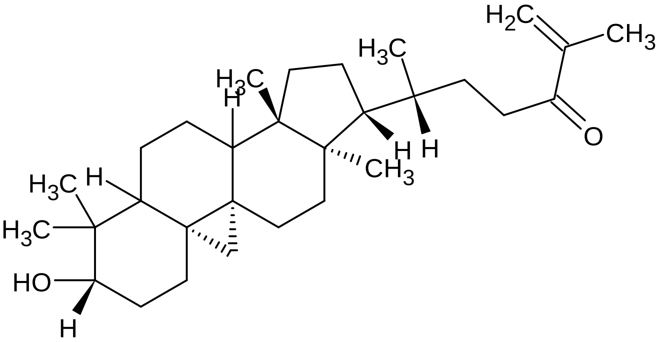

150)

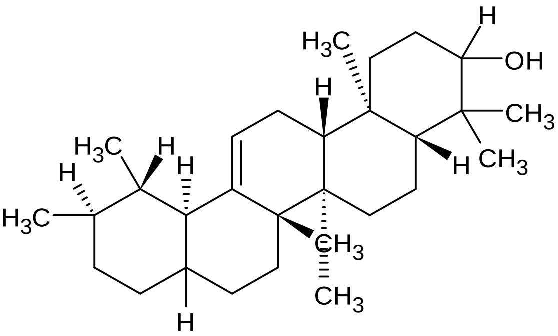

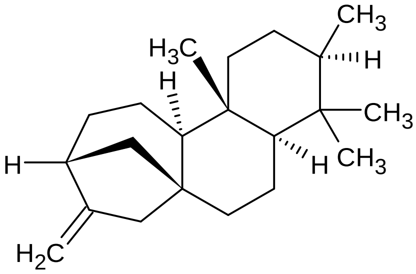

151)

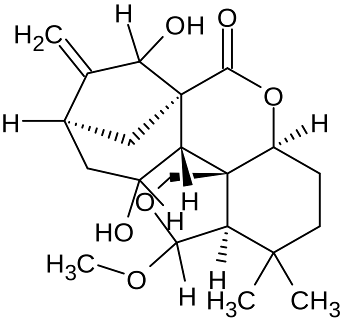

152)

153) is an Organic 1,3-dipolar compound

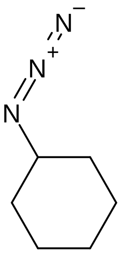

154-169 are Organic acids and derivatives

154) 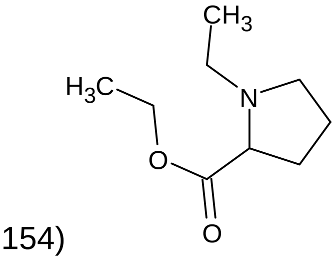

155) 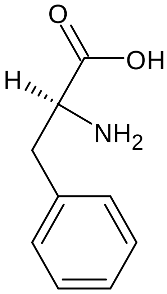

156) 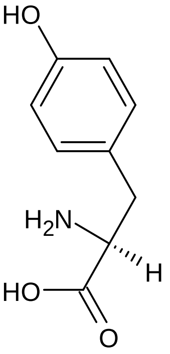

157) 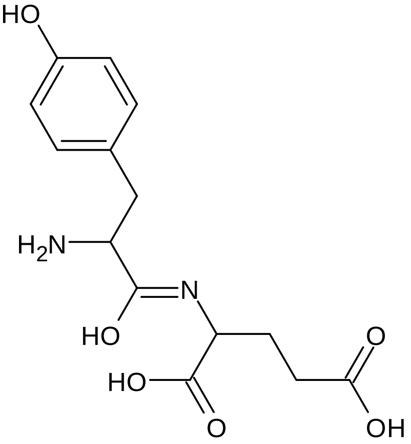

158) 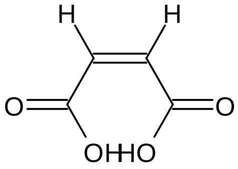

159) 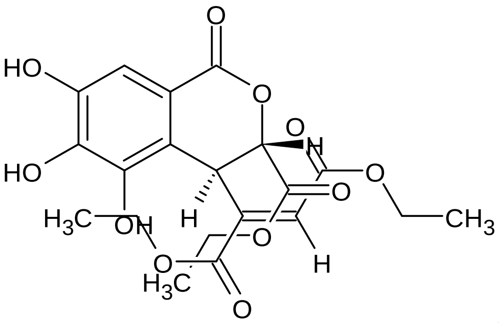

160) 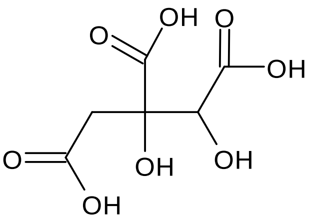

161) 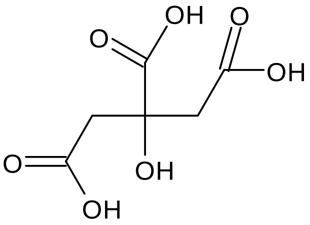

162) 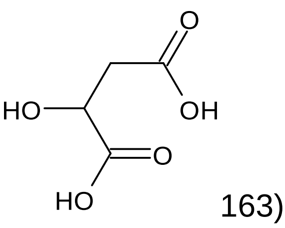

163) 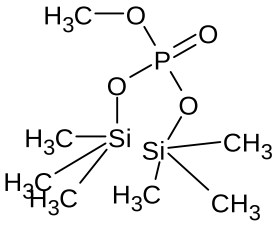

164) 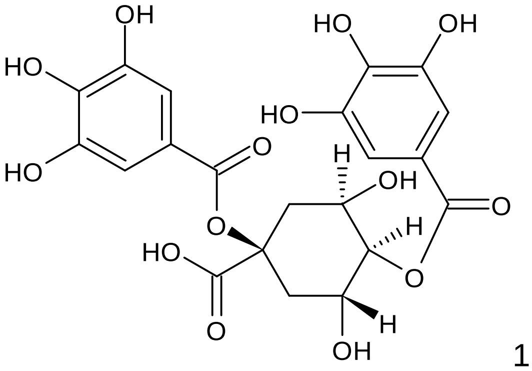

165) 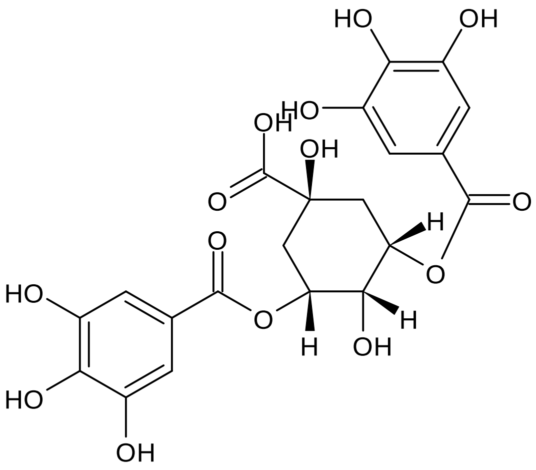

166) 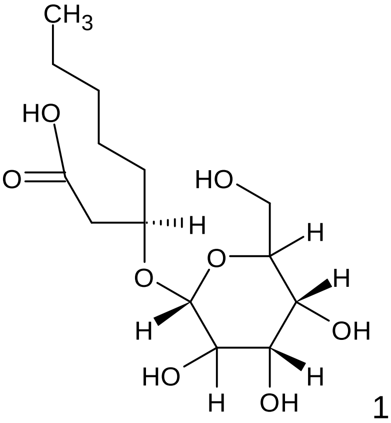

167)

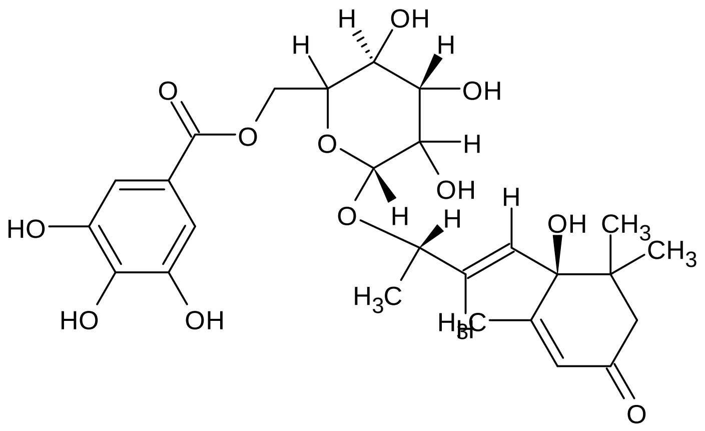

168)

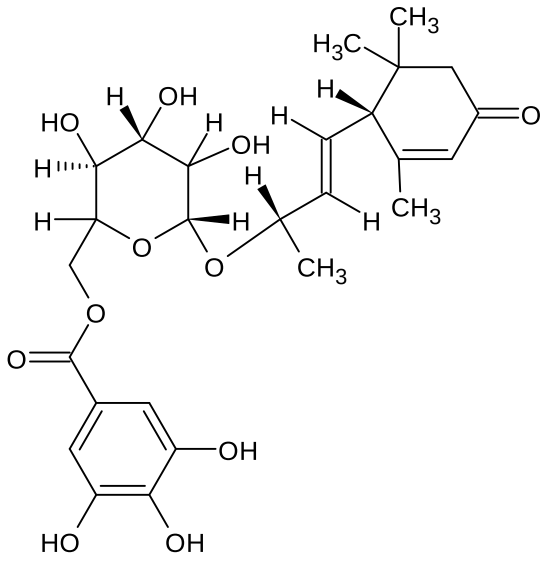

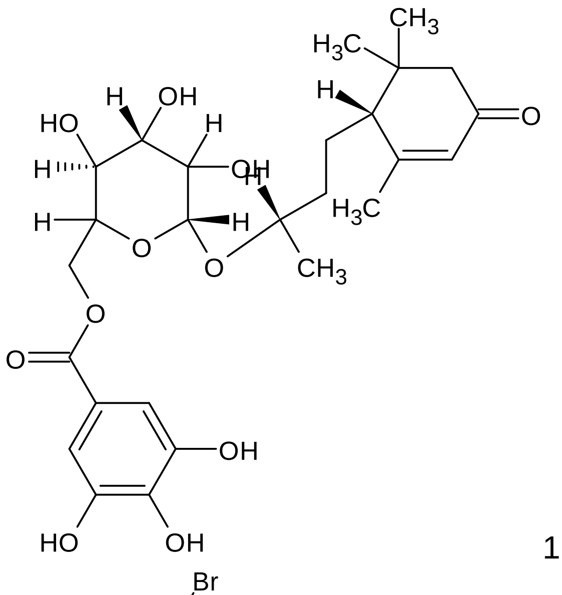

169)

170-171 are Organohalogen compounds

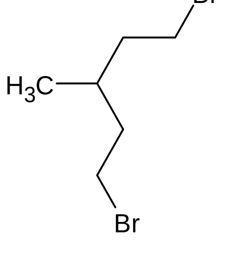

170)

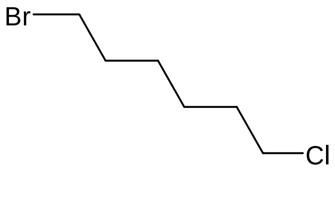

171)

172-184 are Organoheterocyclic compounds

172) 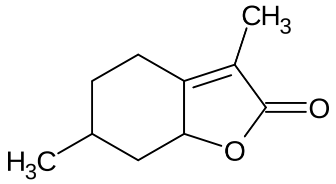

173) 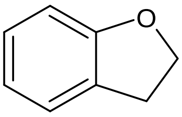

174) 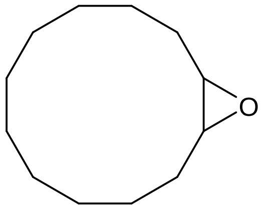

175) 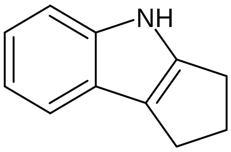

176) 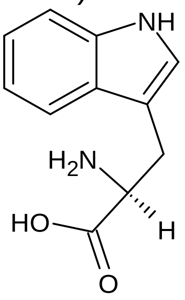

176) 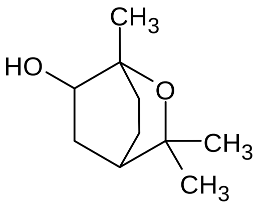

178) 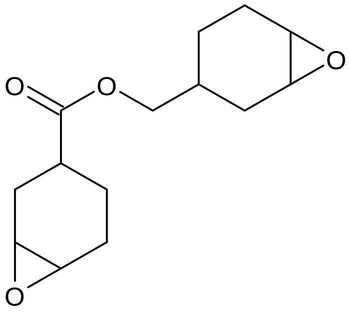

179) 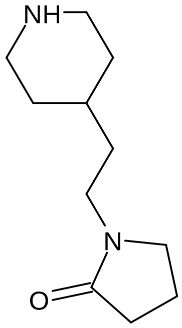

180) 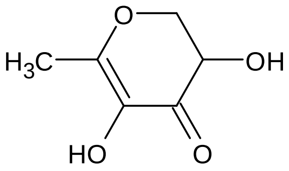

181) 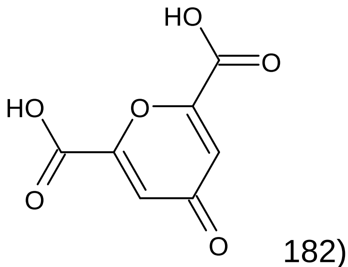

182) 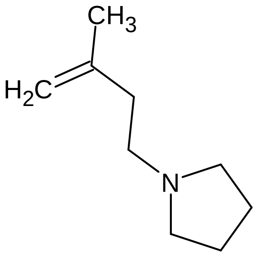

183) 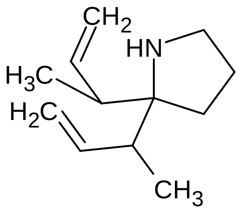

184) 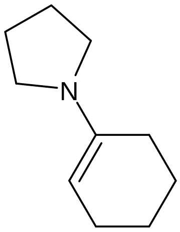

185 is an Organometallic compound

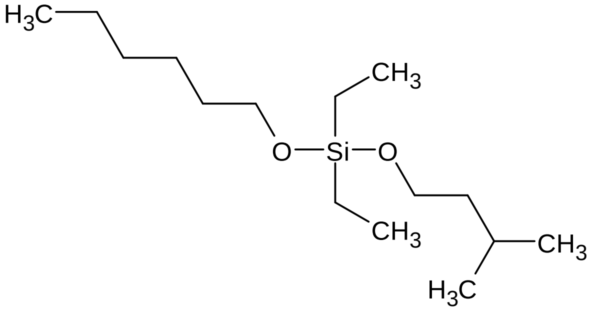

186) is an Organic nitrogen compound

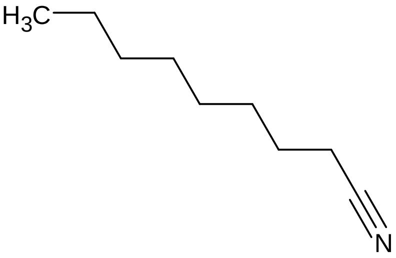

187-215 are Organic oxygen compounds

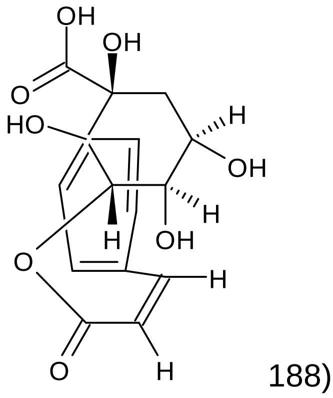

187)

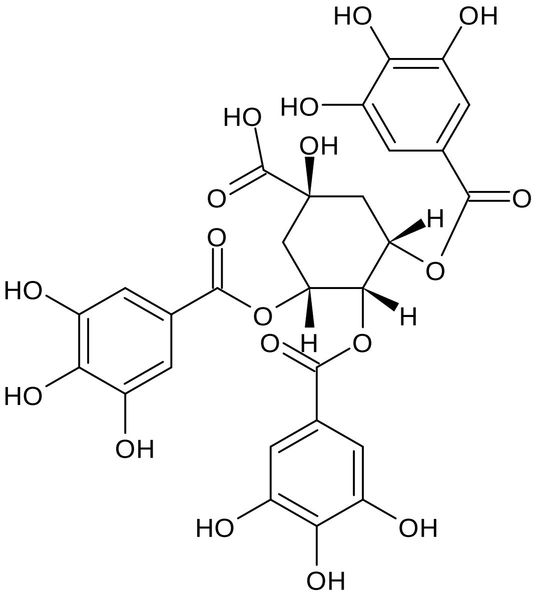

188)

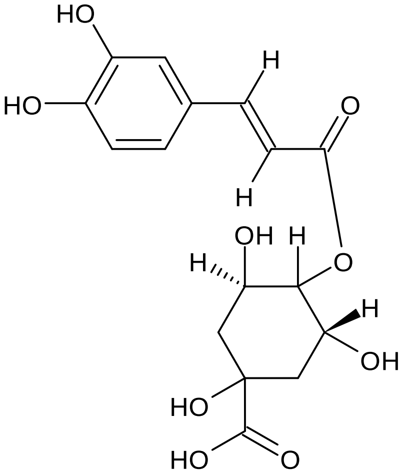

189)

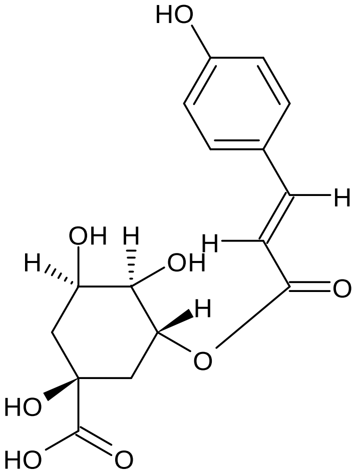

190)

191)

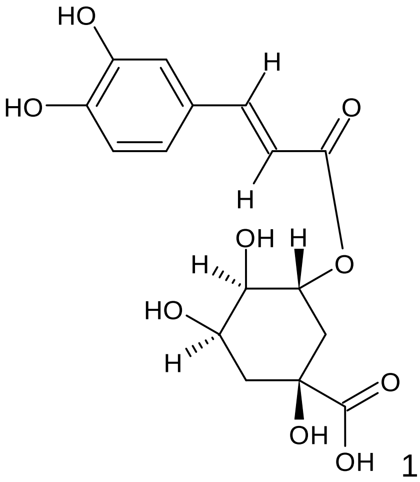

192)

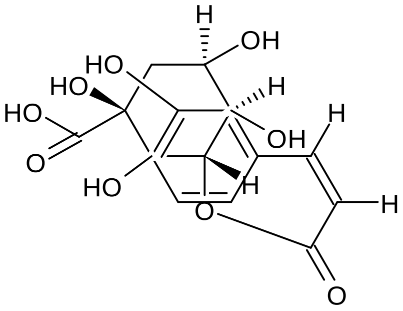

193)

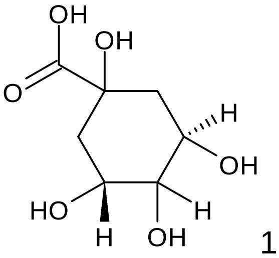

194)

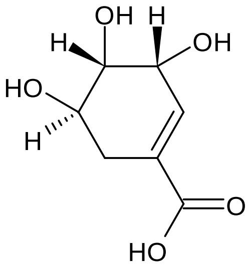

195)

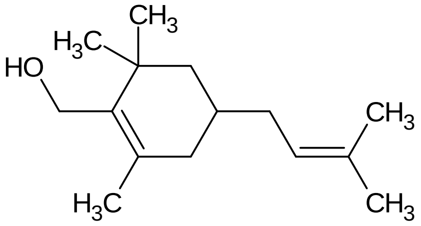

196)

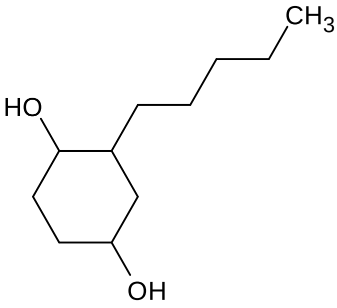

197)

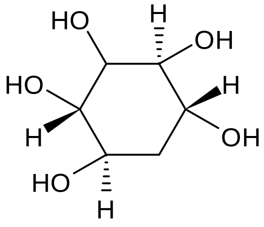

198)

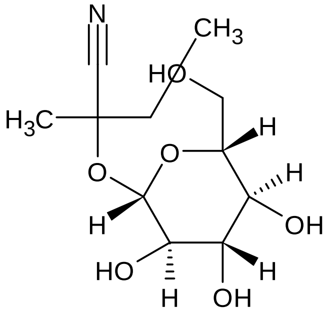

199)

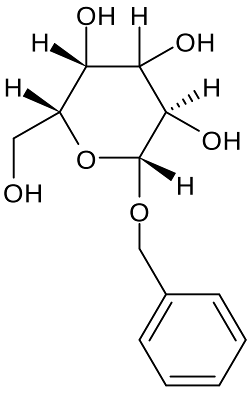

200)

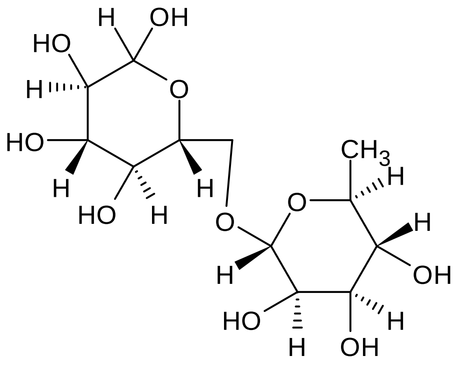

201)

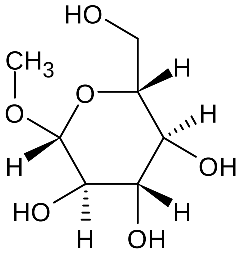

202)

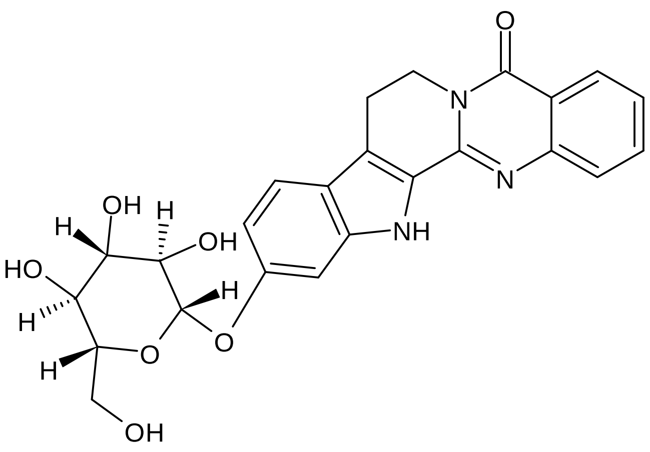

203)

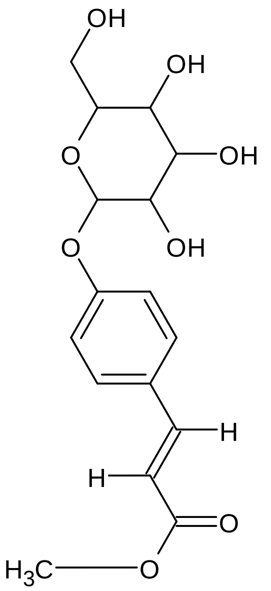

204)

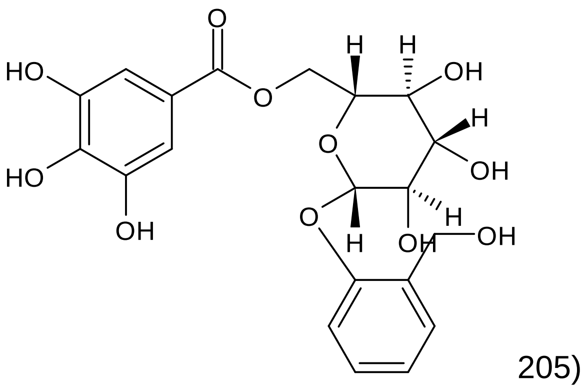

205)

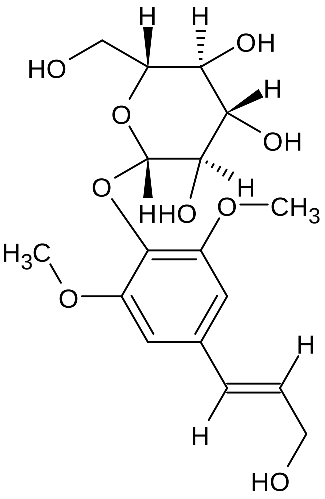

206)

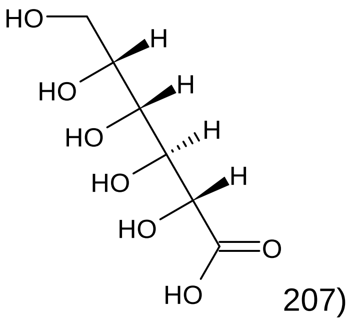

207)

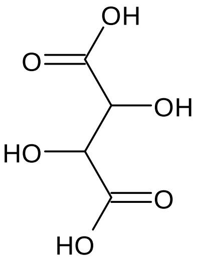

208)

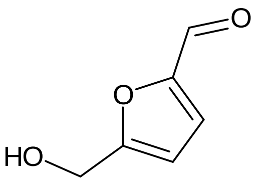

209)

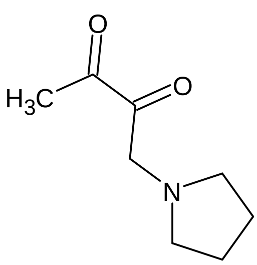

210)

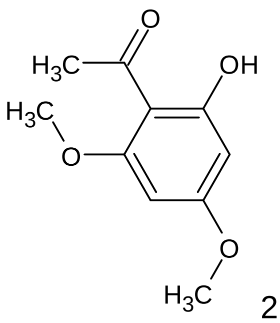

211)

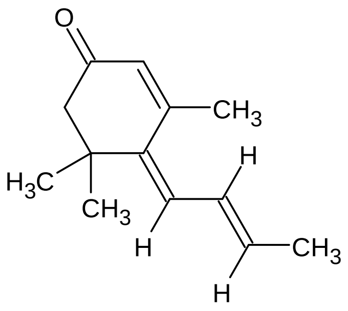

212) 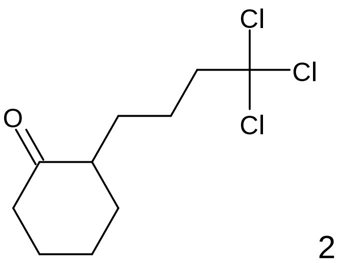

213) 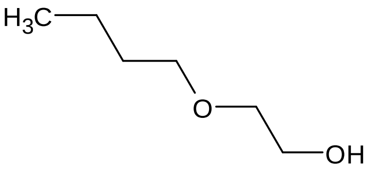

214) 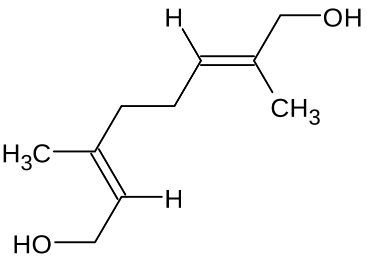

215) $CH_3(CH_2)_{13}CH(CH_3)CH_2OH$

216) is an Organic salt

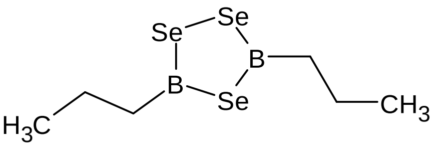

217-298 are Phenylpropanoids and polyketides

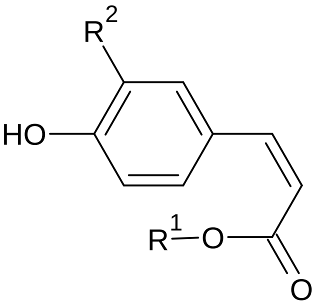

217) R1, -OCH(CH2COOH)COOH; R2, -OMe

218) R1, -H; R2, -H

219) R1, -H; R2, -OH

220) R1, -H; R2, -OMe

221) 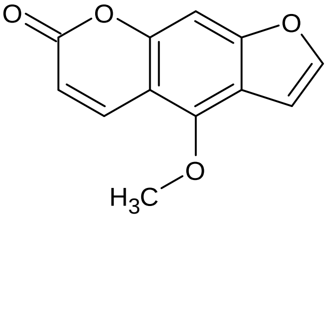

222) 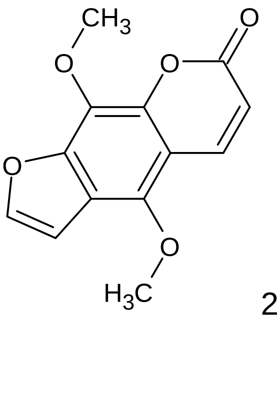

223) 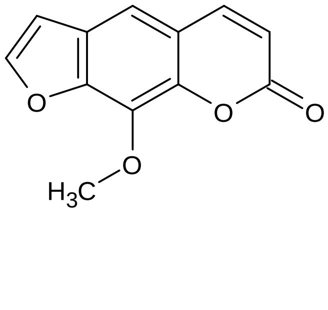

224) 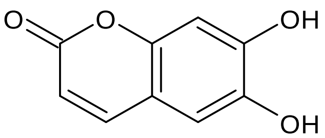

225) 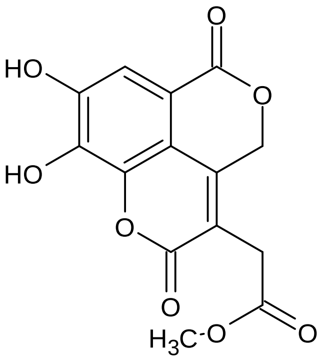

226) 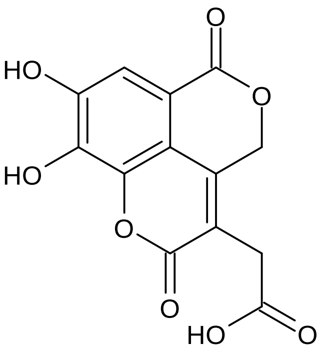

227) 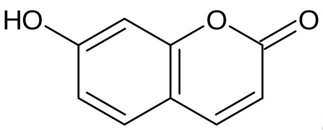

228) 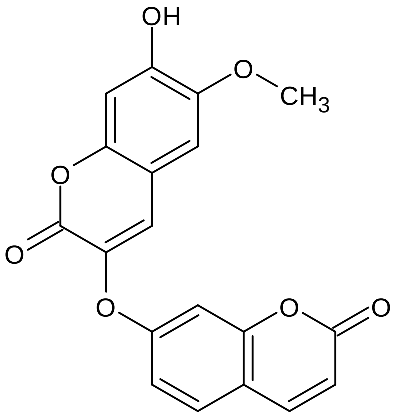

229) 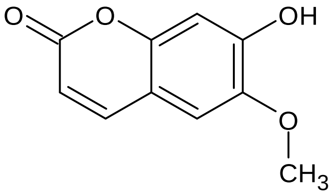

230) 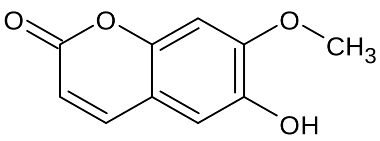

231) 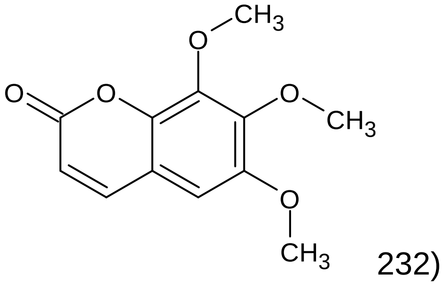

232) 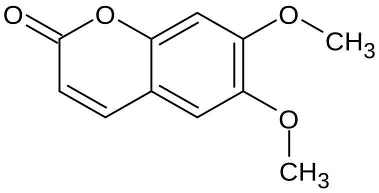

233) 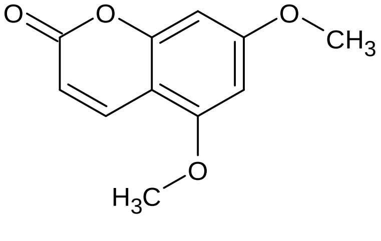

234) 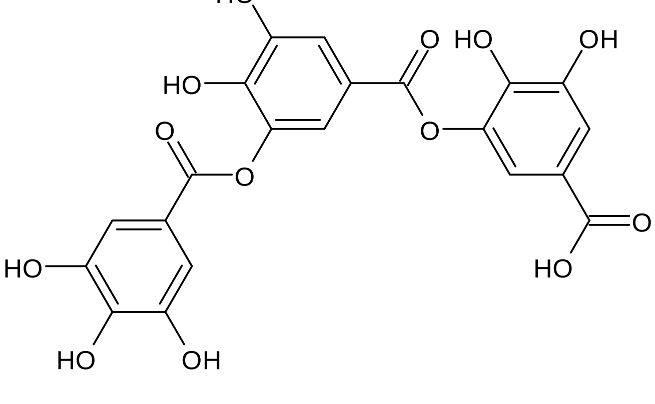

235) 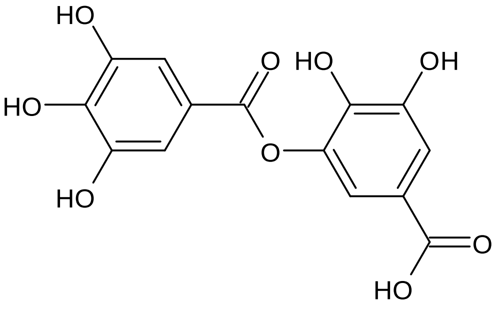

236) 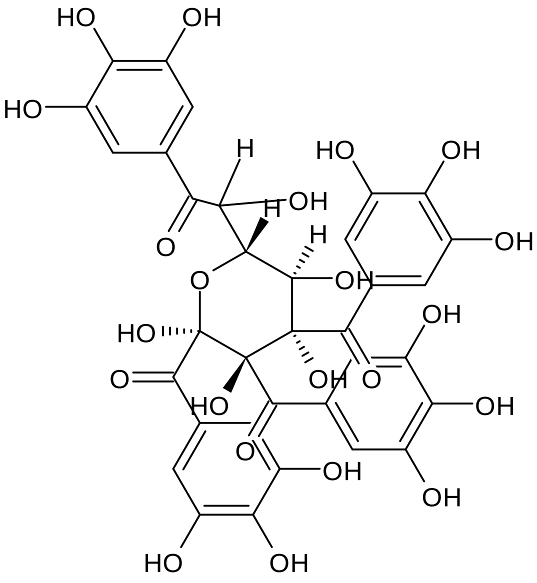

237) 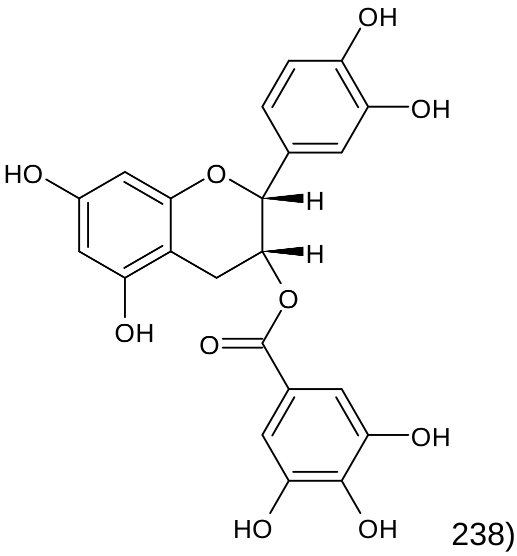

238) 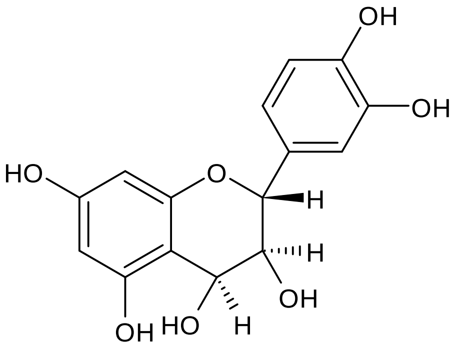

239) 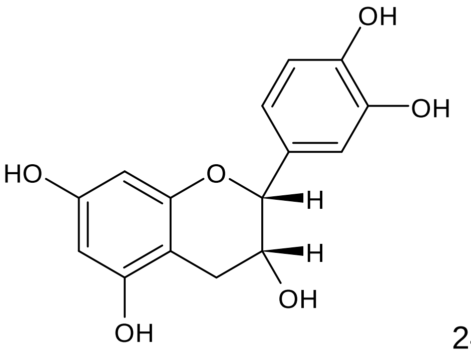

240) 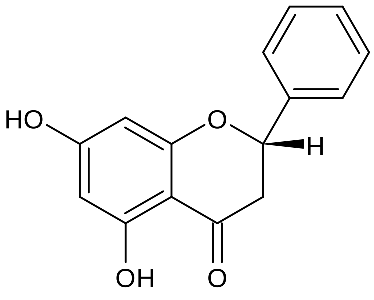

241) 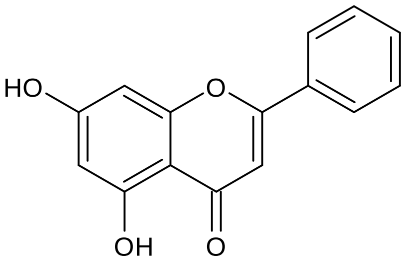

242) 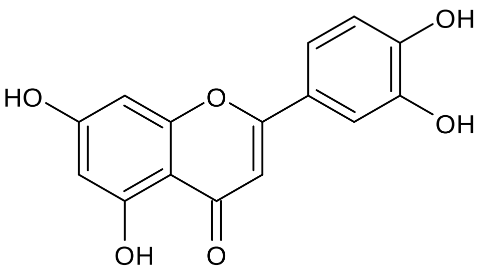

243) 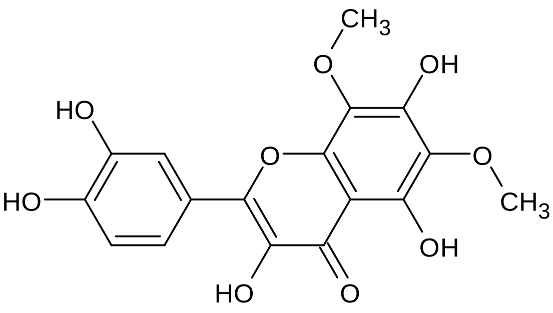

244) 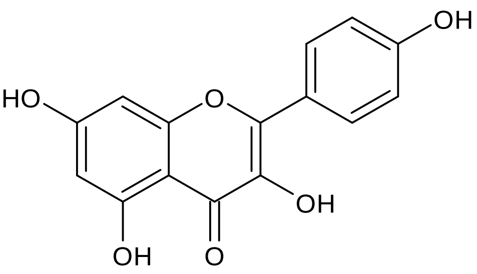

245) 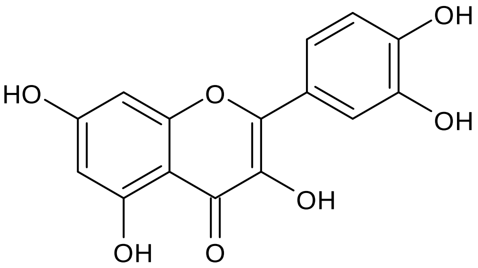

246) 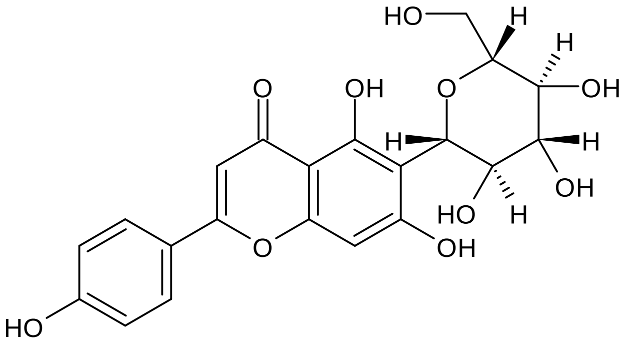

247) 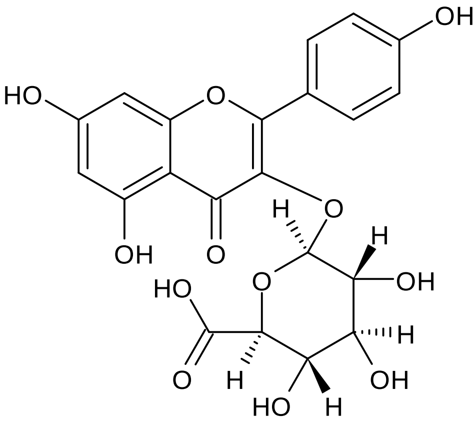

248) 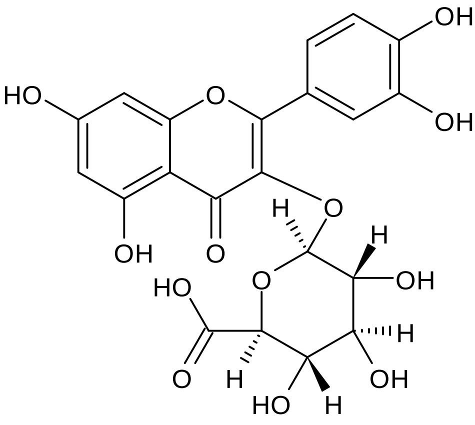

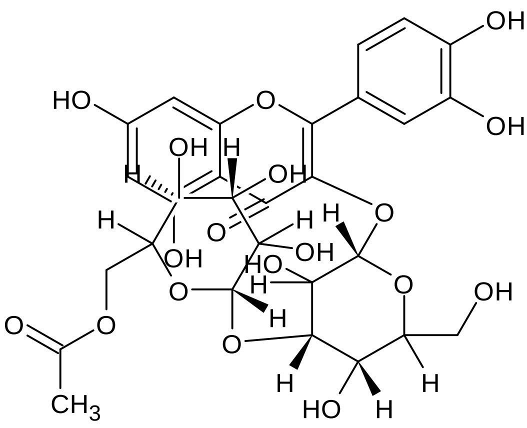

249)

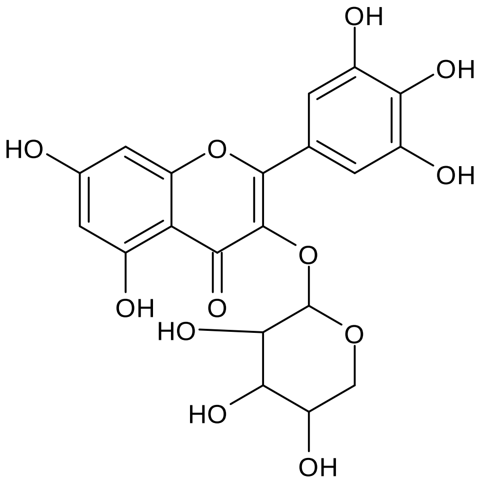

250)

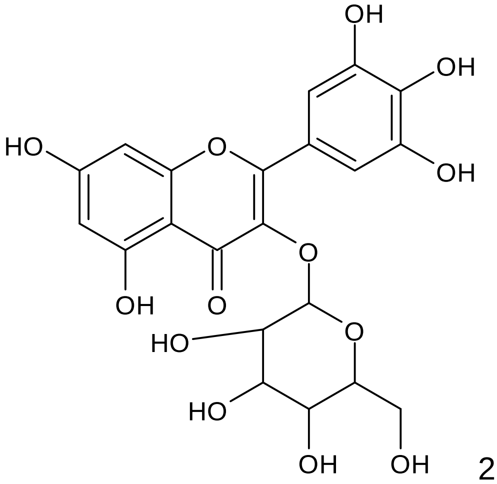

251)

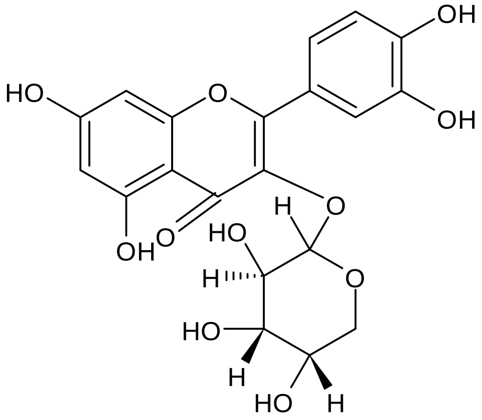

252)

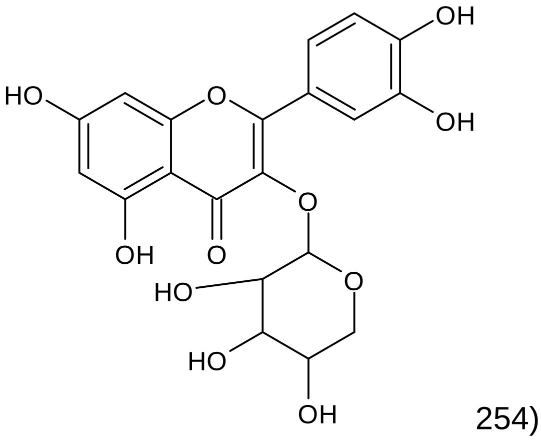

253)

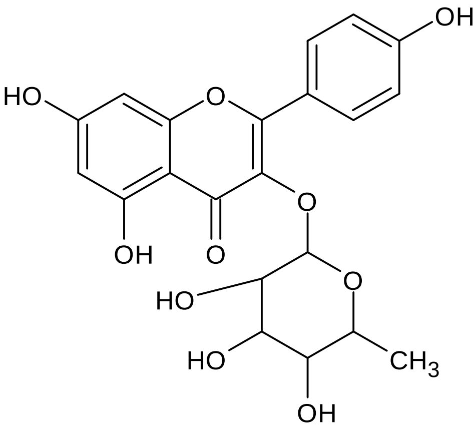

254)

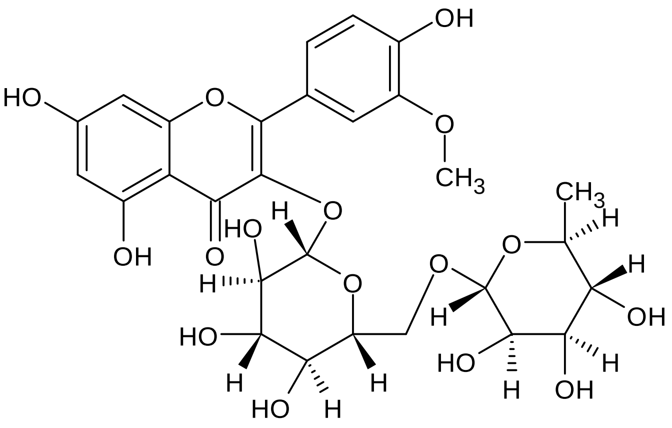

255)

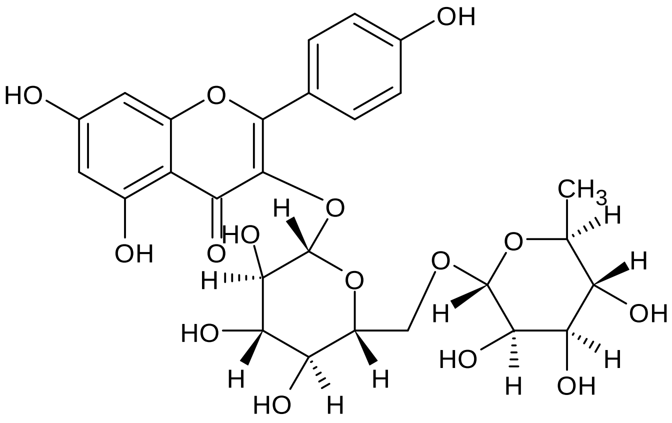

256)

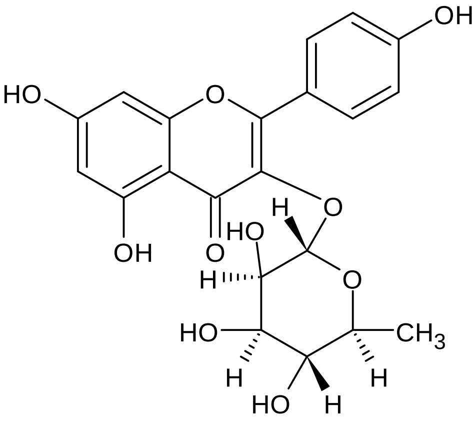

257)

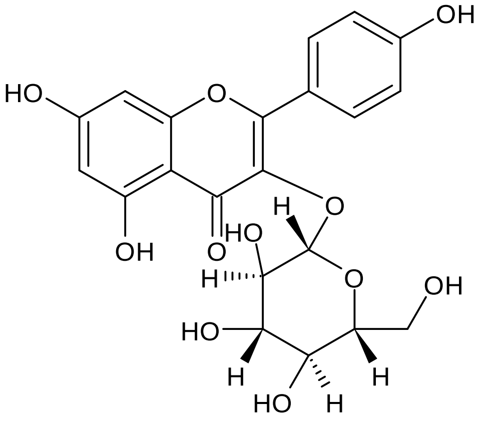

258)

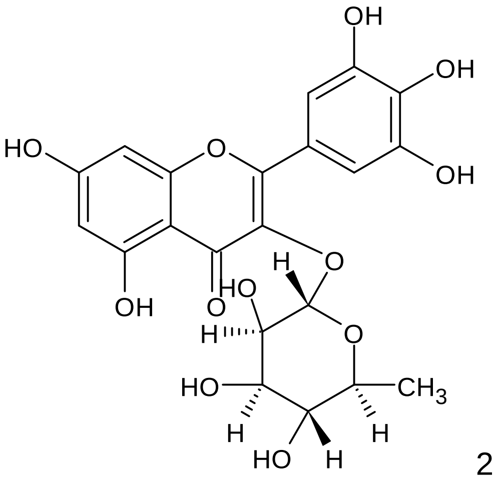

259)

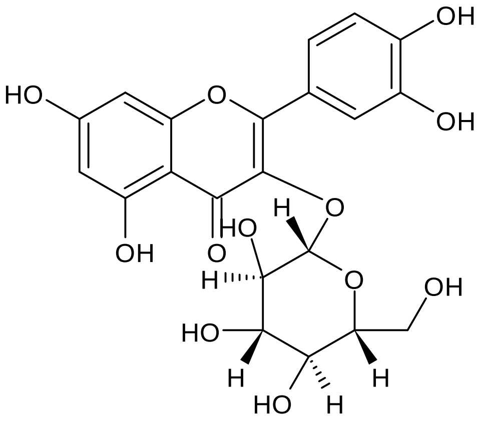

260)

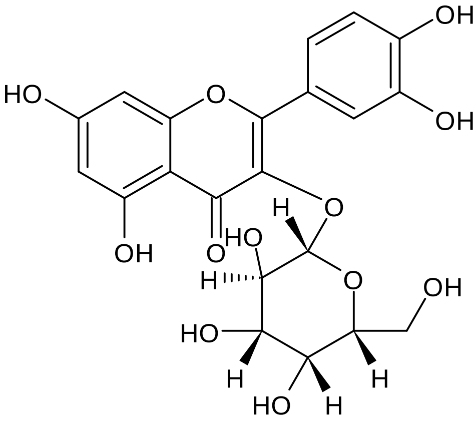

261)

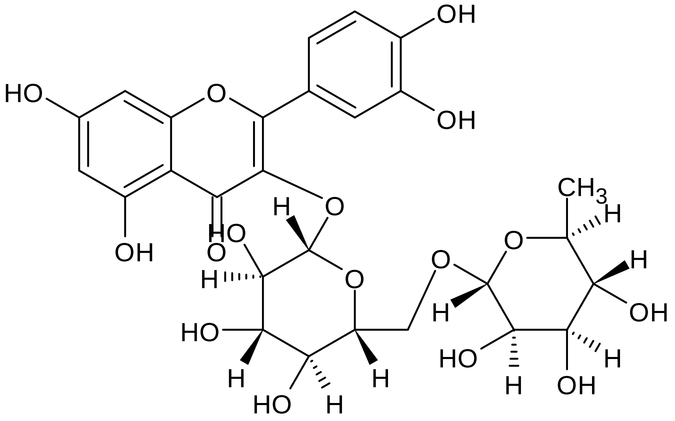

262)

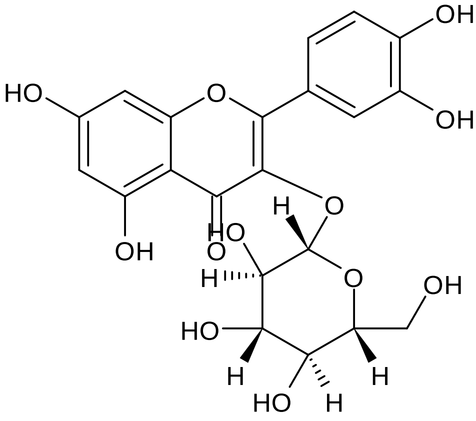

263)

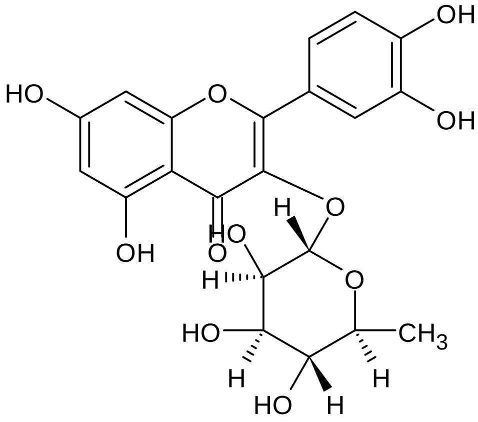

264)

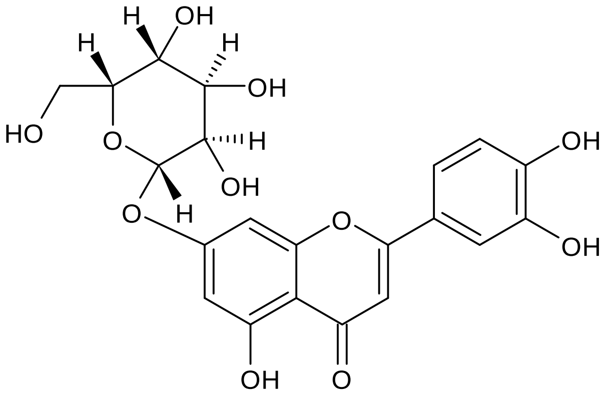

265)

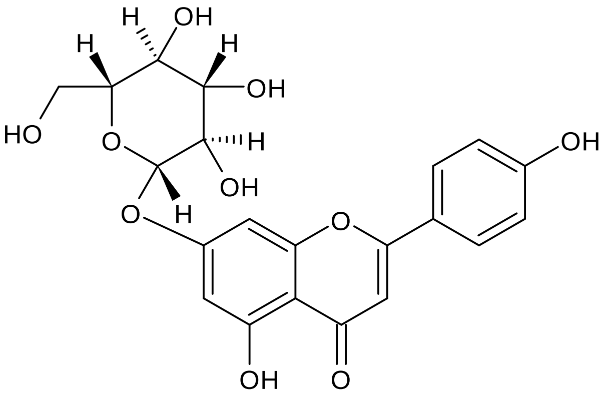

266)

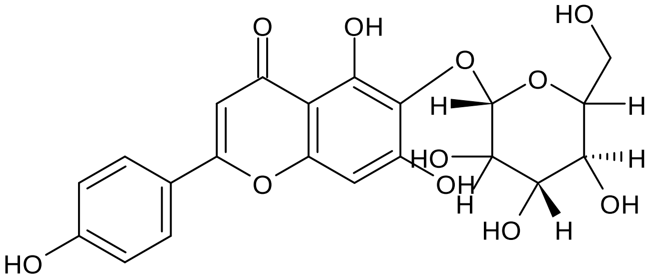

267)

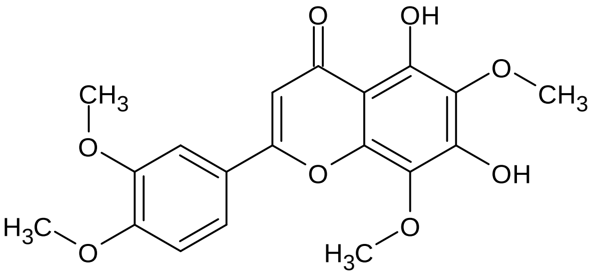

268)

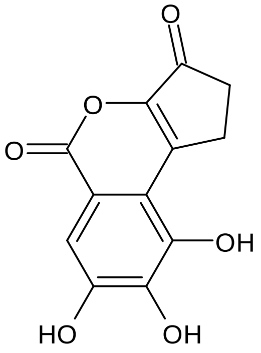

269)

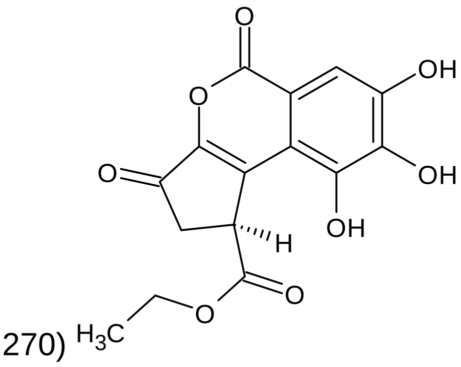

270)

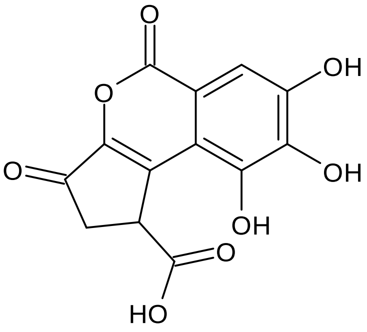

271)

272) 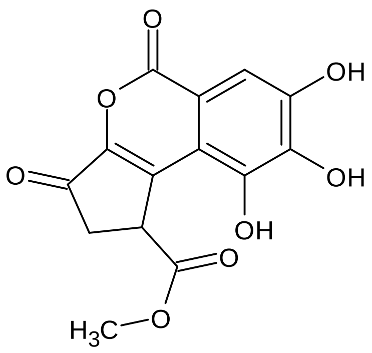

273) 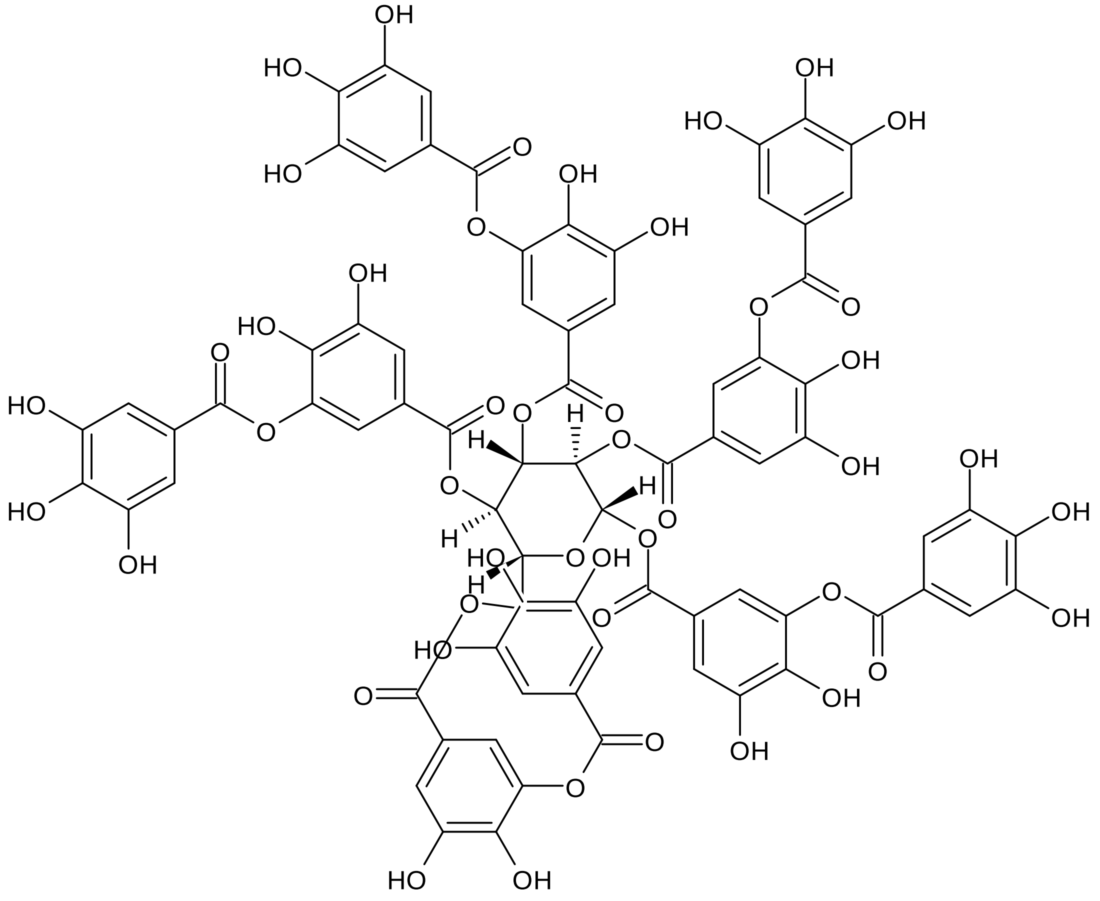

274) 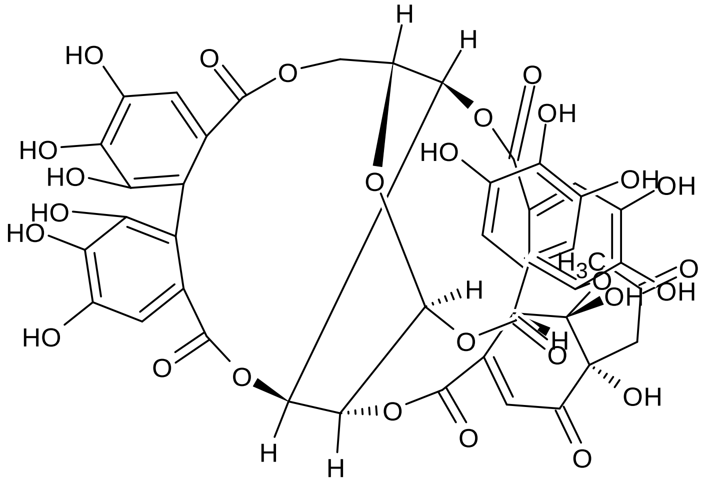

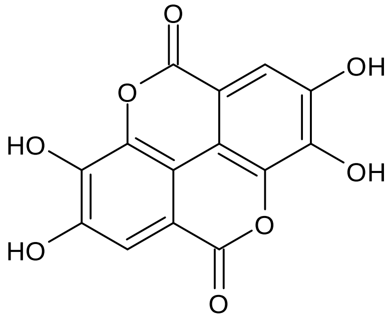
O
OH
O
HO
OH
O
HO
O

275)

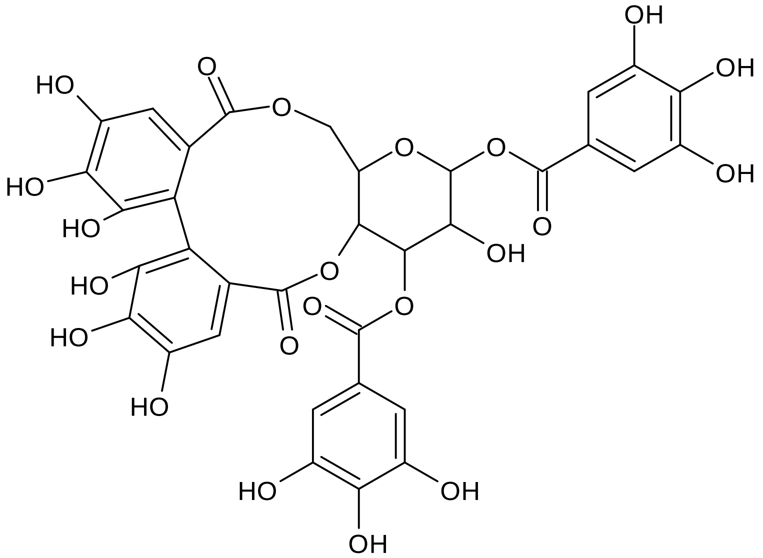
OH
OH
HO
O
O
O
O
OH
HO
HO
O
O
OH
HO
O
O
HO
O
HO
OH
OH

276)

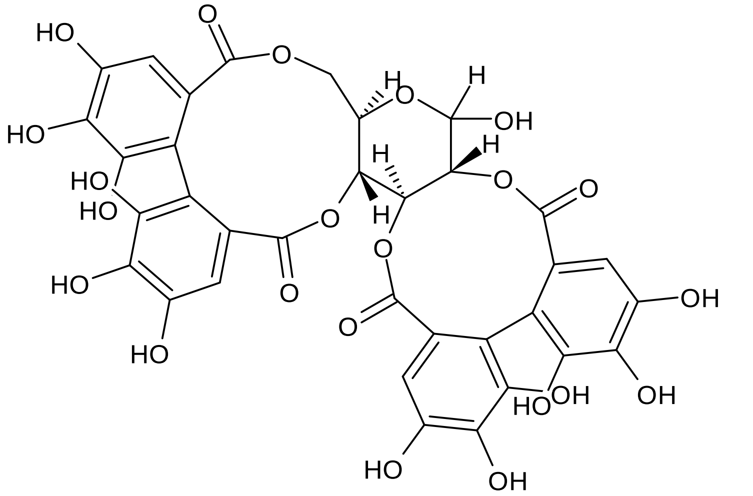
O
HO
O
H
H
O
OH
HO
H
H
HO
HO
O
O
H
O
HO
O
O
OH
HO
O
HO
OH
OH
HO
OH

277)

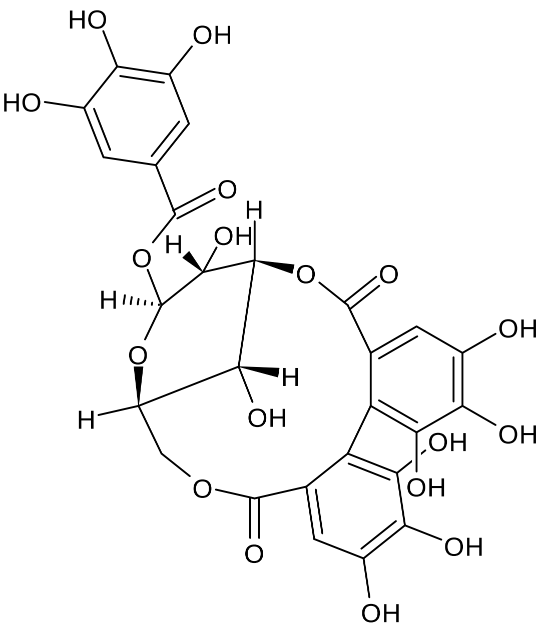

278)

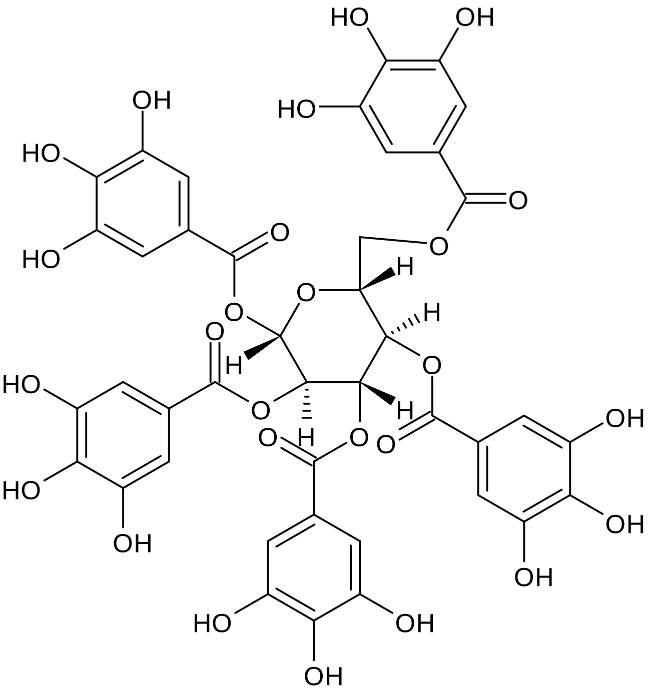

279)

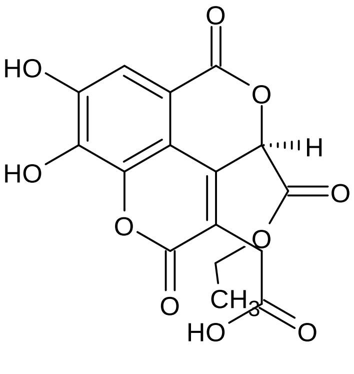

280)

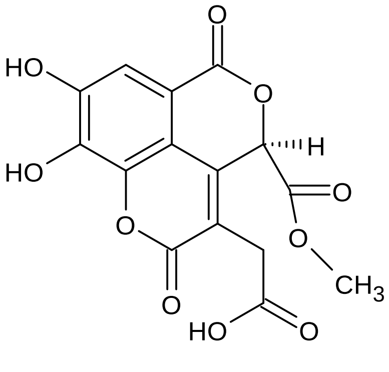

281)

282)

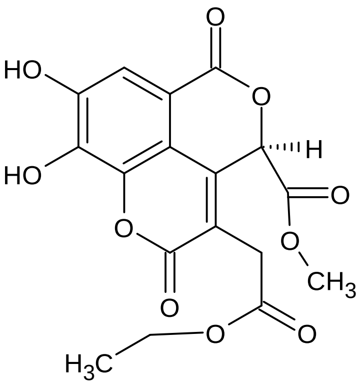

283)

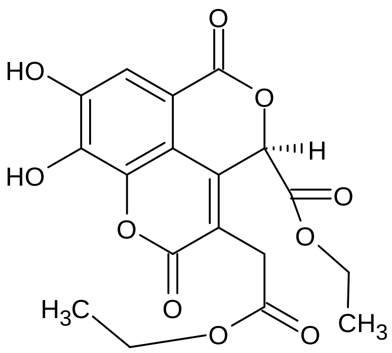

284)

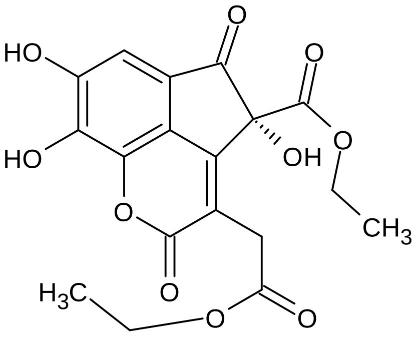

285)

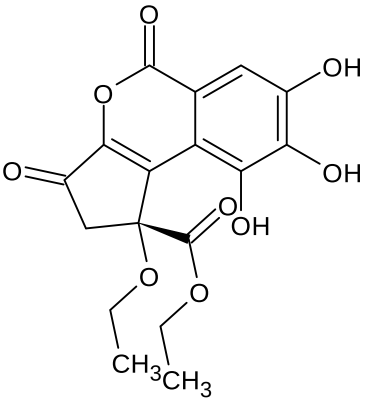

286)

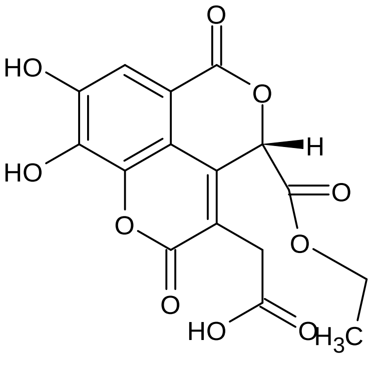

287)

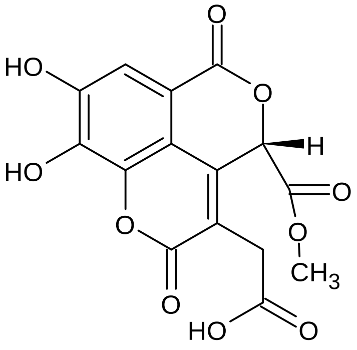

288)

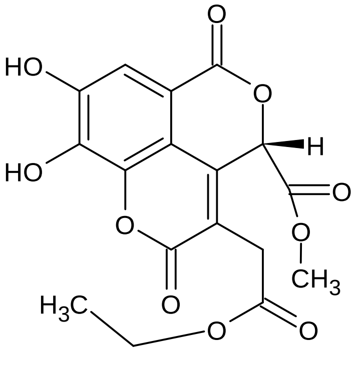

289)

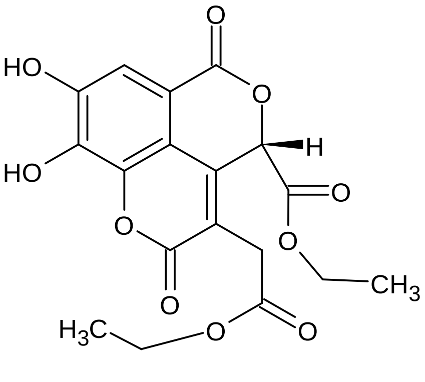

290)

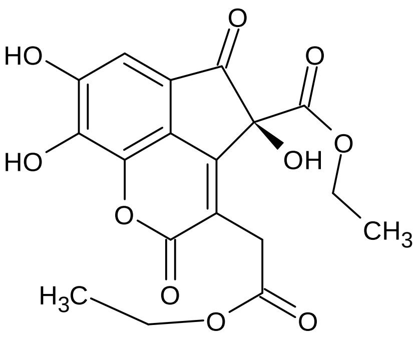

291)

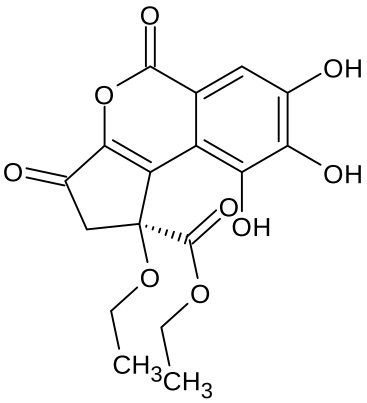

292)

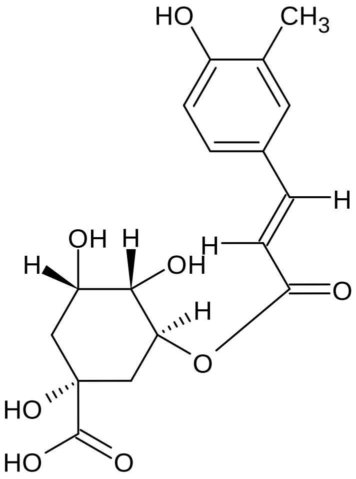

293)

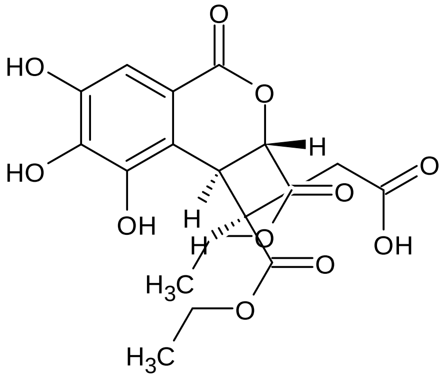

294)

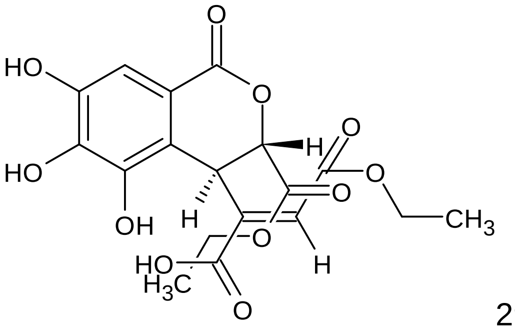

295)

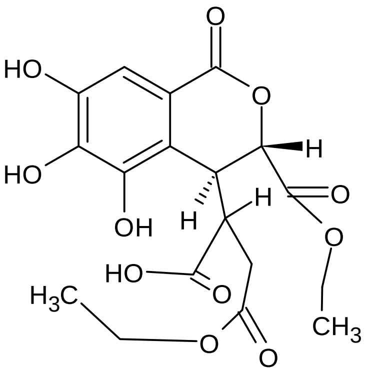

296)

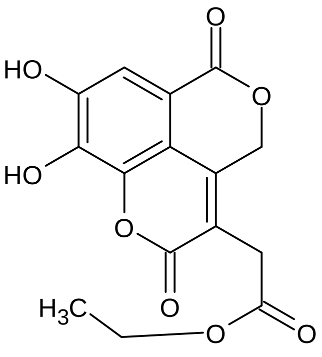

297)

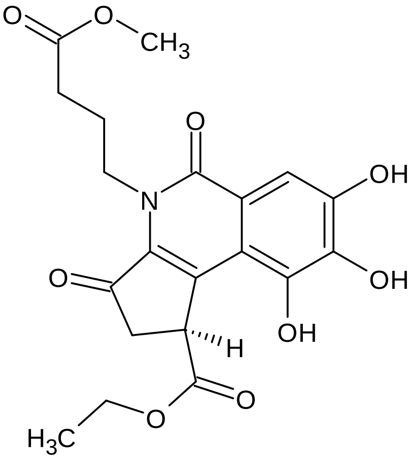

298) 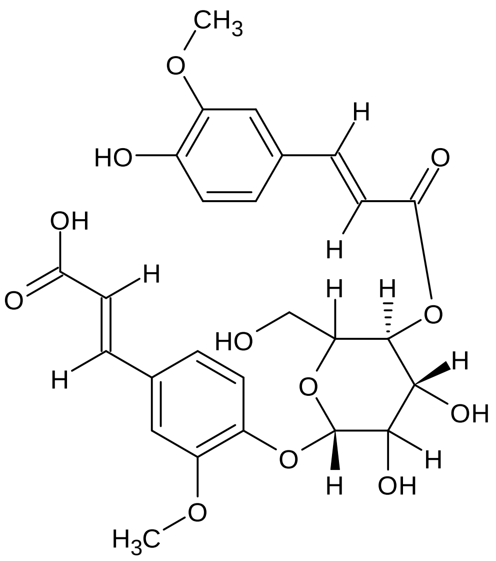

Chemical structures/ of the negative and positive controls used in molecular docking

Negative Controls

N1) 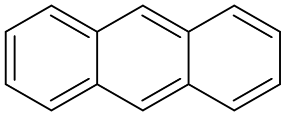 N2) 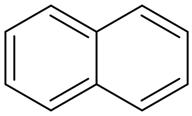 N3) 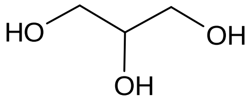

N4) 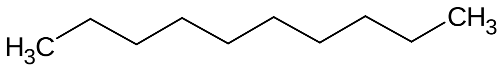 N5) 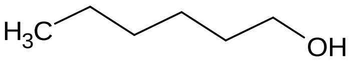

N6) 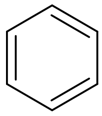 N7) 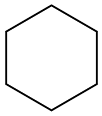 N8) 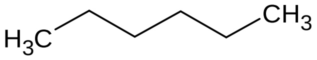

N9) 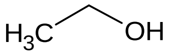 N10) 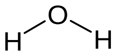

Positive Controls

P1)

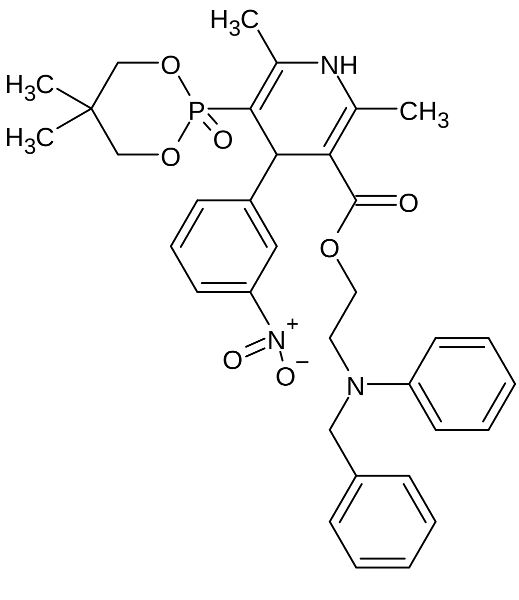

P2)

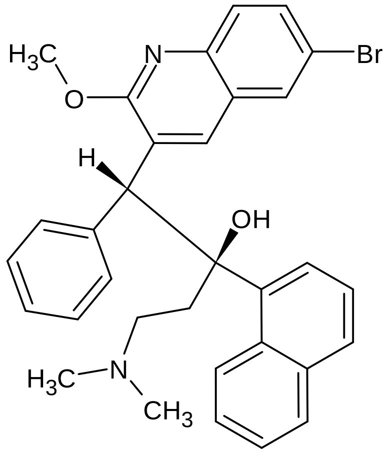

P3)

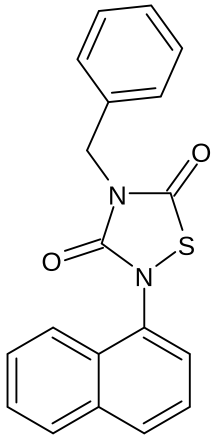

P4)

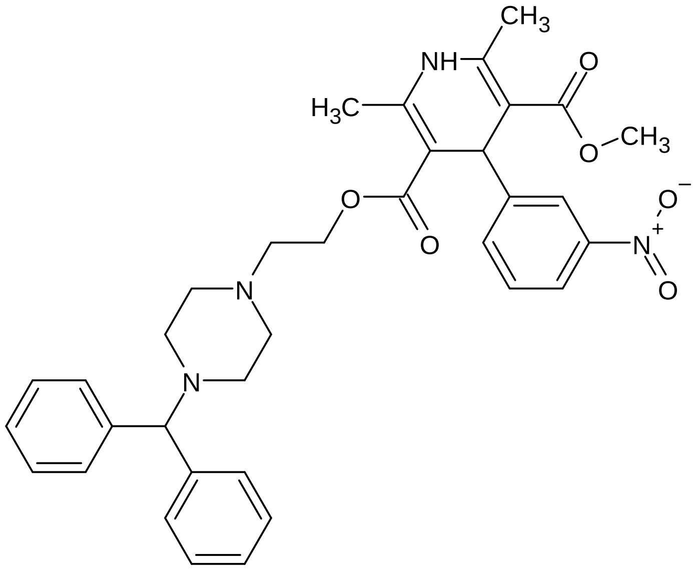

P5)

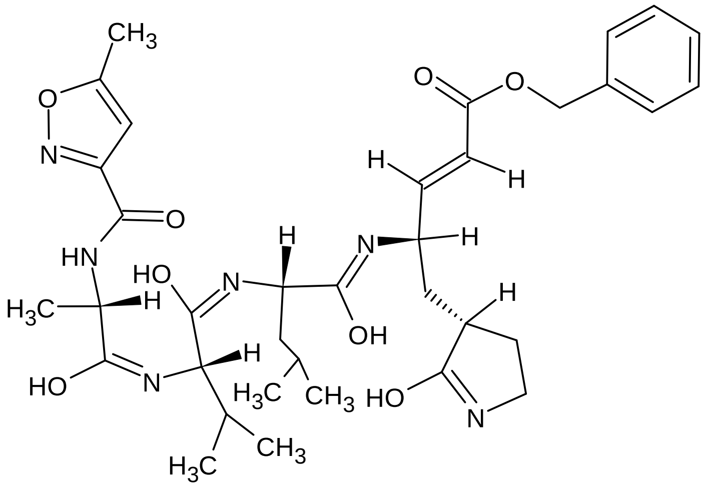

P6)

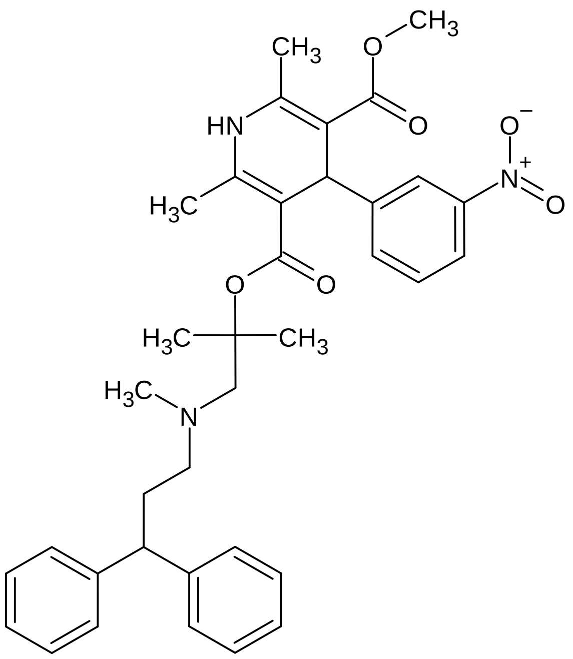

P7)

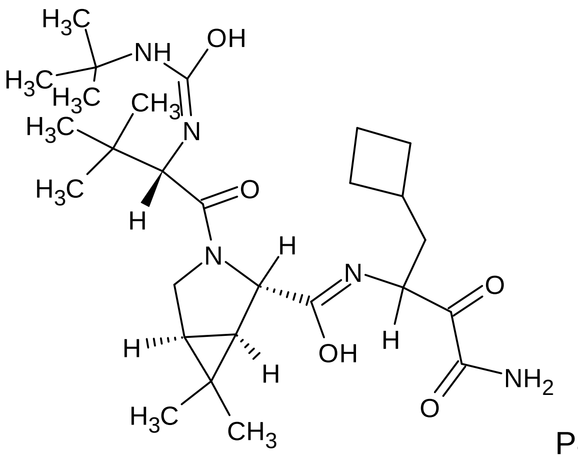

P8)

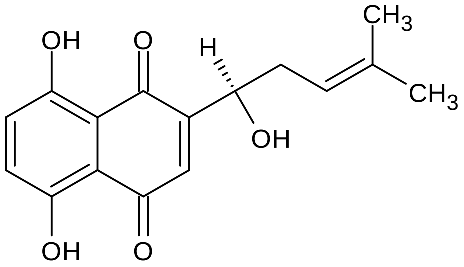

P9)

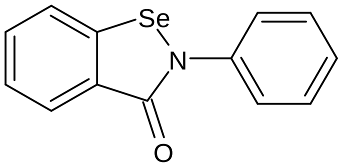

P10)

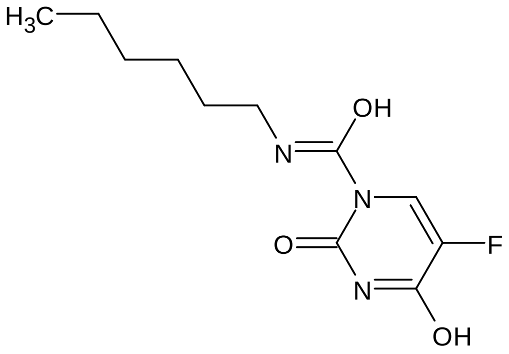

Hierarchy of ClassyFire Chemical Taxonomic Levels

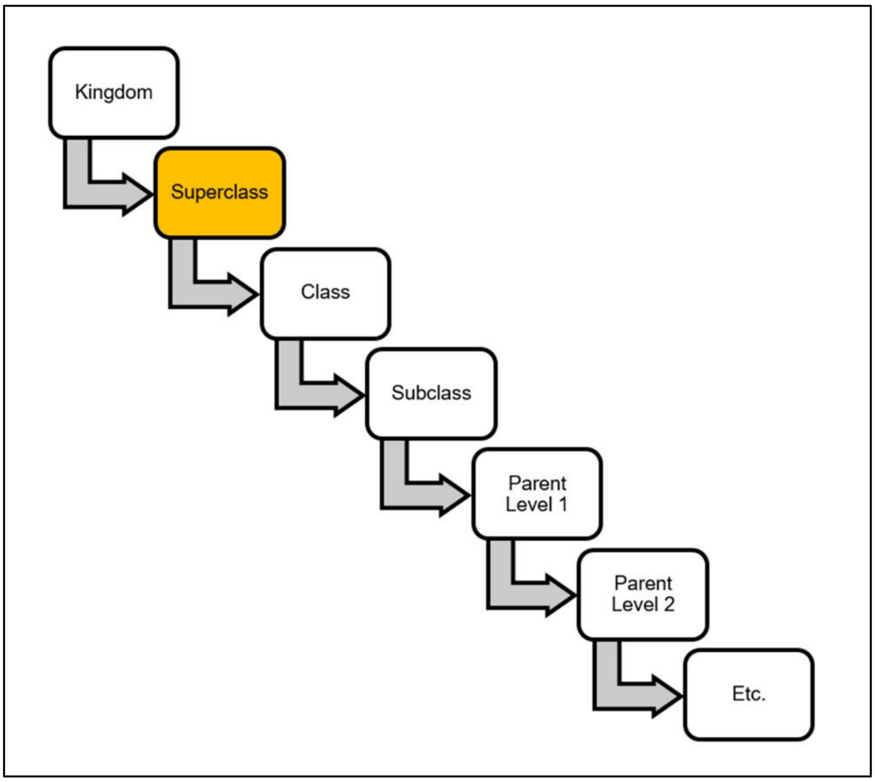

PRISMA Implementation

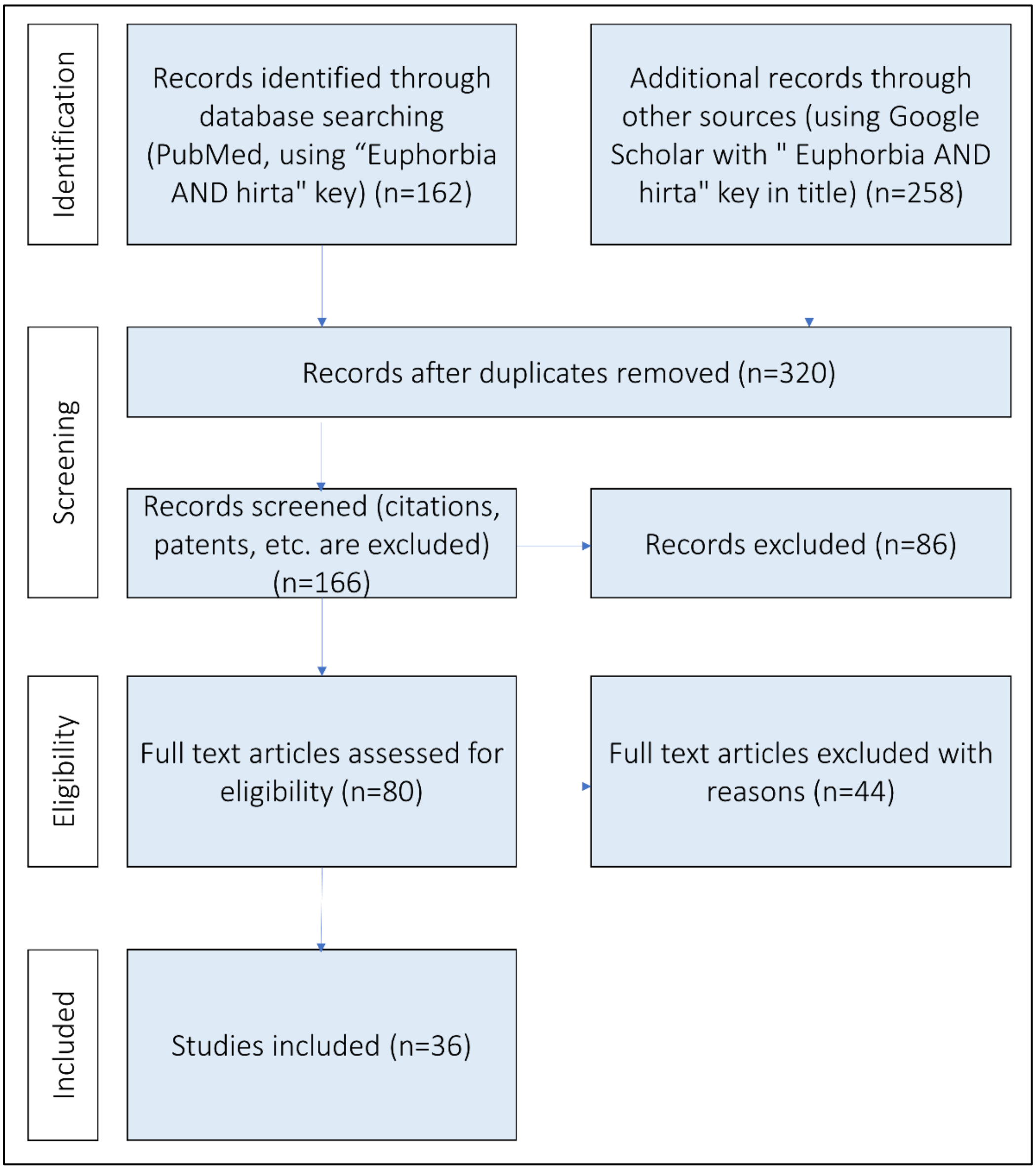

Supplement: Supplementary file 1 [file DataSheet1.PDF]
